# Relay and higher-order thalamic nuclei show an intertwined functional association with cortical-networks

**DOI:** 10.1038/s42003-022-04126-w

**Published:** 2022-11-04

**Authors:** Vinod Jangir Kumar, Christian F. Beckmann, Klaus Scheffler, Wolfgang Grodd

**Affiliations:** 1 Max Planck Institute for Biological Cybernetics, Tuebingen, Germany; 2 Donders Institute for Brain, Cognition, and Behaviour, Centre for Cognitive Neuroimaging, Radboud University, Nijmegen, The Netherlands; 3 Department for Biomedical MagneticResonance, University Hospital Tübingen, Tübingen, Germany

**Keywords:** Neuroscience, Neural circuits

## Abstract

Almost all functional processing in the cortex strongly depends on thalamic interactions. However, in terms of functional interactions with the cerebral cortex, the human thalamus nuclei still partly constitute a terra incognita. Hence, for a deeper understanding of thalamic-cortical cooperation, it is essential to know how the different thalamic nuclei are associated with cortical networks. The present work examines network-specific connectivity and task-related topical mapping of cortical areas with the thalamus. The study finds that the relay and higher-order thalamic nuclei show an intertwined functional association with different cortical networks. In addition, the study indicates that relay-specific thalamic nuclei are not only involved with relay-specific behavior but also in higher-order functions. The study enriches our understanding of interactions between large-scale cortical networks and the thalamus, which may interest a broader audience in neuroscience and clinical research.

## Abstract (web summary)

Functional connectivity and topic mapping of cortical networks reveal that an overlapping of functions exist within different thalamic nuclei, providing insight into the role of relay and higher-order nuclei with the cortex.

## Introduction

Thalamus nuclei and the cortical regions form interfused complex connectivity loops. Subsequently, these loops play a vital role in cortical processing by routing signals and temporally binding to cortical areas for sensory computations, cortical feedback^1–4^, frequency-specific subcortical and cortical arousal regulation^5^, and control recurrent cortical dynamics^6^. Continuous thalamic recurrent loops achieve predictive coding for different brain states, which work like a clock by utilizing periodic bursting and locked oscillation patterns^7^. Consequently, many studies demonstrate a thalamic involvement in motor, sensory, limbic, cognitive, and high-order functions, including consciousness, working memory, arousal, and attention^8–11^. Therefore, a refined understanding of brain functions requires detailed insights into the cortico-thalamic system, which can serve as a potential biomarker for diagnosing mental and psychiatric disorders. Such a more detailed understanding of cortico-thalamic interactions may also be beneficial for deep-brain stimulation and other interventional approaches. Until today, we are still in an initial state of understanding the details of cortico-thalamic interactions in humans.

Resting-state functional magnetic resonance imaging constitutes a powerful and widely used tool to indirectly examine spontaneous fluctuation of neuronal activity of the human brain using blood oxygen level–dependent contrast in the absence of tasks^12–15^. The spatial extent of such resting state functional networks (RSN) can be mapped by finding spatio-temporal correlations between different cortical areas^16^. Resting-state-fMRI has successfully been applied to determine an increasing number of cortical networks^17,18^. Moreover, it has been shown that most RSN are continuously and dynamically active even at rest and that a spatial correspondence exists between behavioral and resting-derived connectivity networks^18,19^. In a seminal paper, Smith et al.^12^ revealed a domain-specific behavioral correspondence of cortical areas with the spatio-temporal properties of 10 different RSN.

However, intact cortico-cortical functional connections require the thalamus. Therefore, cortically determined RSN conveys only partial information and fails to deliver a complete picture of how the functional networks act as a system in the brain. Several studies have examined thalamic participation in functional networks^20–27^, during task performance 28–31, and the rest^28,29^. However, thalamic analyses always face a particular difficulty, as their structural subdivisions cannot be satisfactorily distinguished with in vivo imaging. Consequently, the definition of nuclei and nuclei groups predominantly relies on histology and experimental studies^30–32^, and a functional attribution to different behavioral domains is still lacking.

Therefore, we investigated the functional connectivity between the thalamus and RSN from a large sample of 730 healthy subjects of the Human Connectome Project (HCP). The analysis assessed network-specific connectivity mapping and a gradual mode of percent connectivity of individual thalamic nuclei, nuclei subgroups, and the whole thalamus. Using topic mapping, the cortical RSN associated with different mental functions also uncovers nuclei-specific involvement within the thalamus.

Cytoarchitectonic characterization of resting-state functional networks shows overlapping cortical areas, indicating multiplicity in some areas while others are specific to a network. The analysis revealed that the networks communicate to distinct and partly overlapping thalamic nuclei, differing in extent and intensity, indicating the specific and synergistic effect of the overlapping cortical areas within different networks. Interestingly, most networks show an extensive involvement with the thalamic nuclei suggesting that intertwined sets of behavioral domains are shared across the different networks. Specifically, the right frontoparietal network shows the highest, and the cerebellar network the lowest connectivity with the left thalamus. The sensorimotor nuclei are also involved in the default-mode, left & right frontoparietal, and executive networks. The laterality differences exist in connectivity as well as in the topic analysis. Furthermore, correlation and topic analysis put forward the higher-order functional role of relay nuclei. In summary, the study enhances the understanding of cortico-thalamic connectivity at the level of nuclei, nuclei sub-groups, and the whole thalamus.

## Results

### Cytoarchitectonic characterization of RSN

**Fig. 1:**
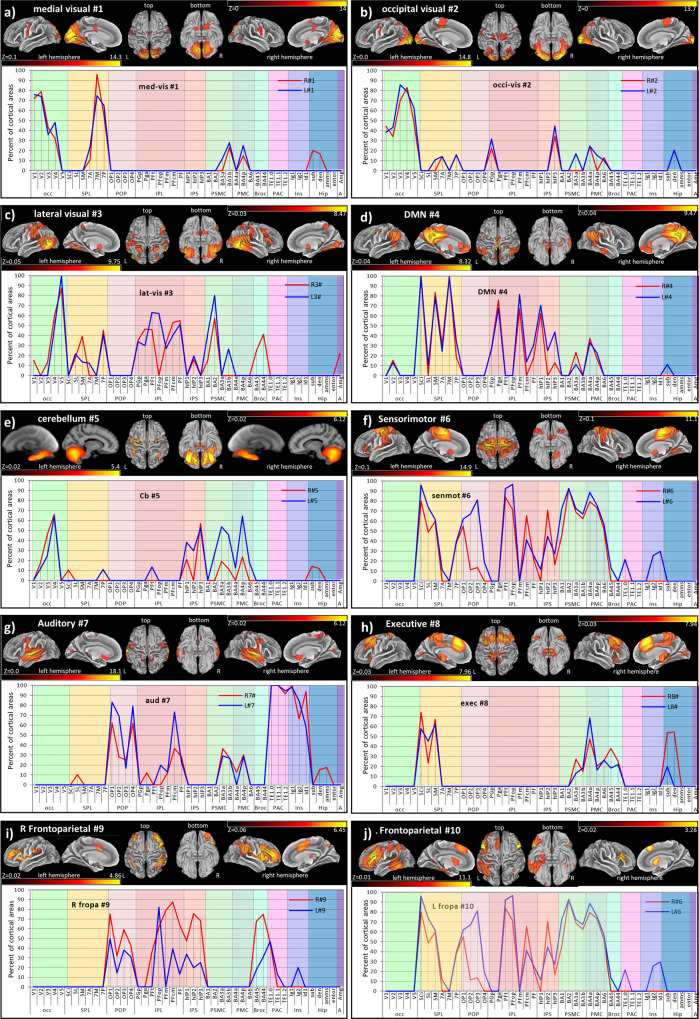
Cytoarchitectonic Characterization of Smith-10 brain maps. Surface views (top) and plotted percent overlap (bottom) of cortical areas according to the Jülich Histology atlas (bottom), which covered ≥10%. The *X*-axis depicts the cortical areas within the Jülich atlas. For Abbreviations: see Supplementary Table 1 (which includes all overlaps). The *Y*-axis indicates the percent overlap of the functional network with the atlas labels in the Jülich atlas. The color scale (*z*) of the visualized RSN aligned brain maps: **a** MV: Left, 0.1–14.3 right 0–14; **b** OV: Left, 0–14.8 right 0 13.7; **c** LV: Left, 0.05–9.75 right 0.03–8.47; **d** DMN: Left 0.04–8.32, right 0.04–9.47; **e** CB: Left 0.02–5.4, right 0.02–6.12; **f** SM: Left 0.1–14.9, right 0.1–11.1; **g** AU: Left 0–18.1, right 0–17; **h** EX: Left 0.03–7.96, right 0.03–7.94; **i** RF: Left 0.02–4.86, right 0.06–6.45; **j** LF: Left 0.1–11.1, right 0.02–3.28.

Using the Jülich and Brodmann histological atlases, our cytoarchitectonic characterization depicts all major cortical areas and subdivisions of the different RSN within the occipital parietal, temporal, and frontal lobes (Fig. 1). The cytoarchitectonic maps show specific and overlapping cortical areas associated with the different functional networks (Supplementary Tables 1–3 and Note 1), revealing that the human cortex’s intrinsic functional architecture displays many areas that house spatio-temporal functions while others associate with a single spatio-temporal network. Details of the different behavioral domains of the RSN, as listed in Supplementary Table 4, also possess overlapping functional domains (https://brainmap.org/taxonomy/behaviors/). (Highly associated: Supplementary Table 4 and all (Fig. 2 in ref. ^12^).

### Thalamus connectivity with RSN

**Fig. 2:**
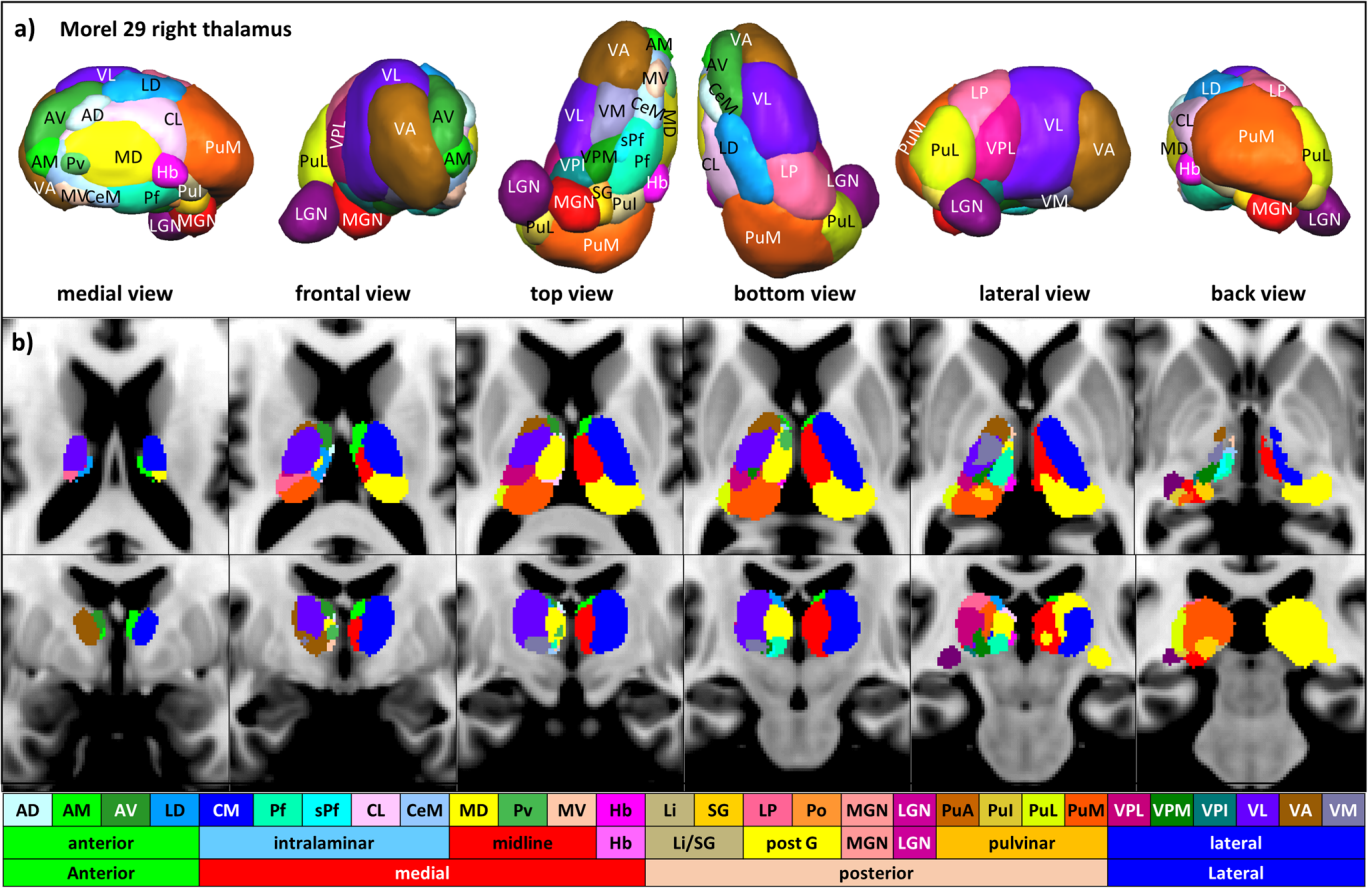
Anatomy of the thalamus. **a** 3D Rendered views of 29 thalamic nuclei of Morel’s histological atlas with abbreviations. **b** Depiction of thalamic nuclei and nuclei groups of the Morel atlas six axial and coronal views (Krauth et al.^92^). The nuclei depiction is color-coded with respect to each nucleus. The detailed color assignments in hex color code: AD (CBFFFF), AM (41FB30), AV (359430), LD (1AA0FC), MD (FFFC38), CM (002CFB), Pf (3FFDB6), sPf (3CFEFE), CL (FDCAFE), CeM (98C9FD), Pv (52B755), MV (FDC8AC), Hb (F933FC), Li (C0B47F), SG (FECE30), LP (FA6897), Po (FC963F), MGN (FA141B), LGN (711172), PuA (C56419), PuI (DCC642), PuL (DCFC36), PuM (FA571F), VPL (C2187B), VPM (1C7F13), VPI (177877), VL (612DFB), VA (965B15), VM (797AA6).

**Fig. 3:**
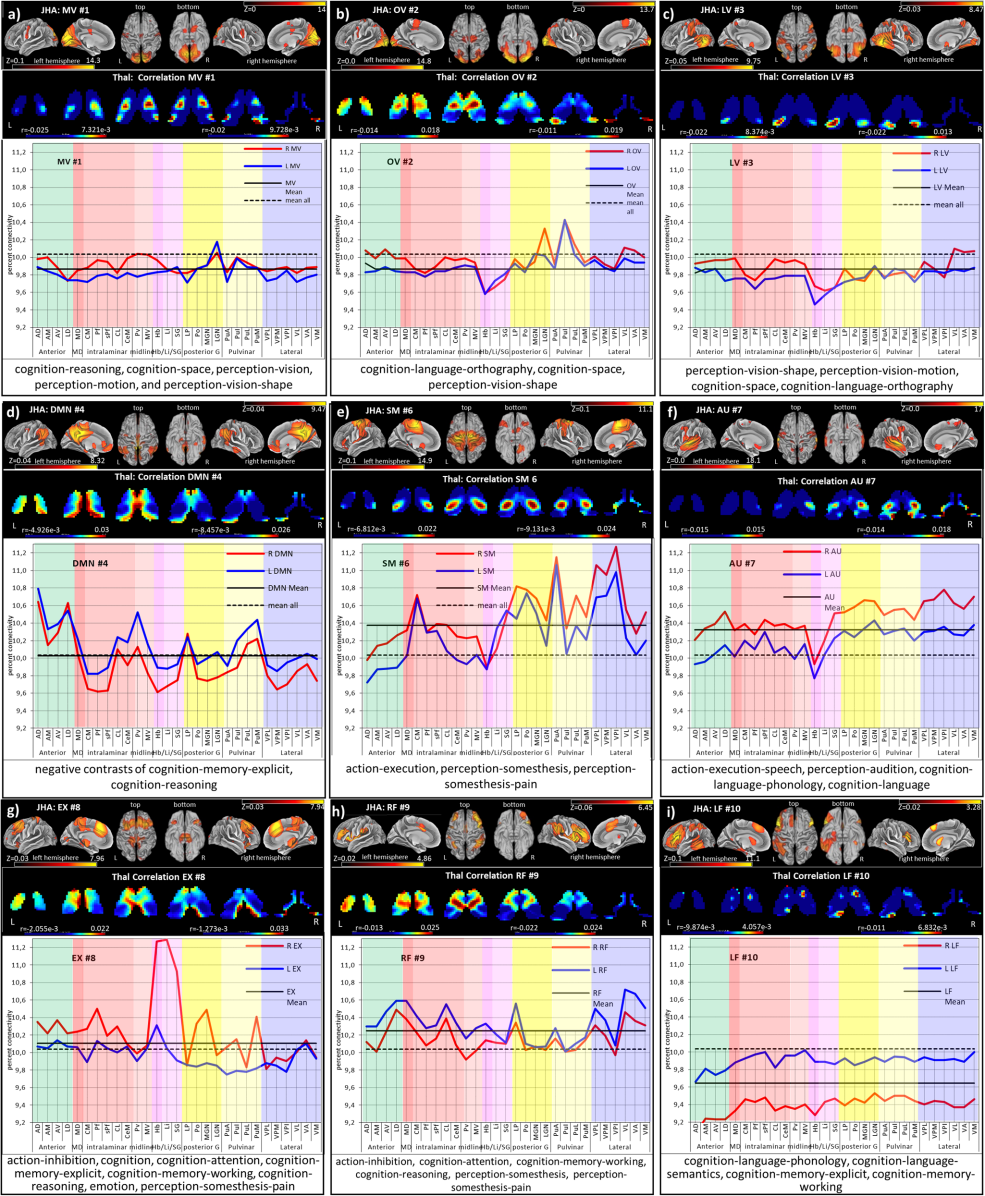
Cortico-thalamic connectivity of 9 cortical RSN. Each subplot depicts surface views of functional networks, Corresponding correlations maps of the thalamus, and percent connectivity thalamic nuclei; highly associated behavioral domains. Abbreviations are mentioned in Supplementary Table 4. See Supplementary Table 5 for the nuclei names. The color bar depicts the correlation maps within each network, i.e., the left and right color bars for the left and right thalamus. Scales according to the network specific maps are as following: **a** MV: Left −0.025 to 7.321e−3, right −0.02 to 9.728e−3; **b** OV: Left −0.014 to 0.018, right −0.011 to 0.019; **c** LV: Left −0.022 to 8.374e−3, right −0.022 to 0.013; **d** DMN: Left −4.926e−3 to 0.03, right −8.457e−3 to 0.026; **e** SM: Left −6.812e−3 to 0.022, right −9.131e−3 to 0.024; **f** AU: Left −0.015 to 0.015, right −0.014 to 0.018; **g** EX: Left −2.055e−3 to 0.022, right −1.273e−3 to 0.033; **h** RF: Left −0.013 to 0.025, right −0.022 to 0.024; **i** LF: Left −9.874e−3 to 4.057e−3, right −0.011 to 6.832e−3. The cortical maps’ color bar scales are also given in Fig. 1.

**Fig. 4:**
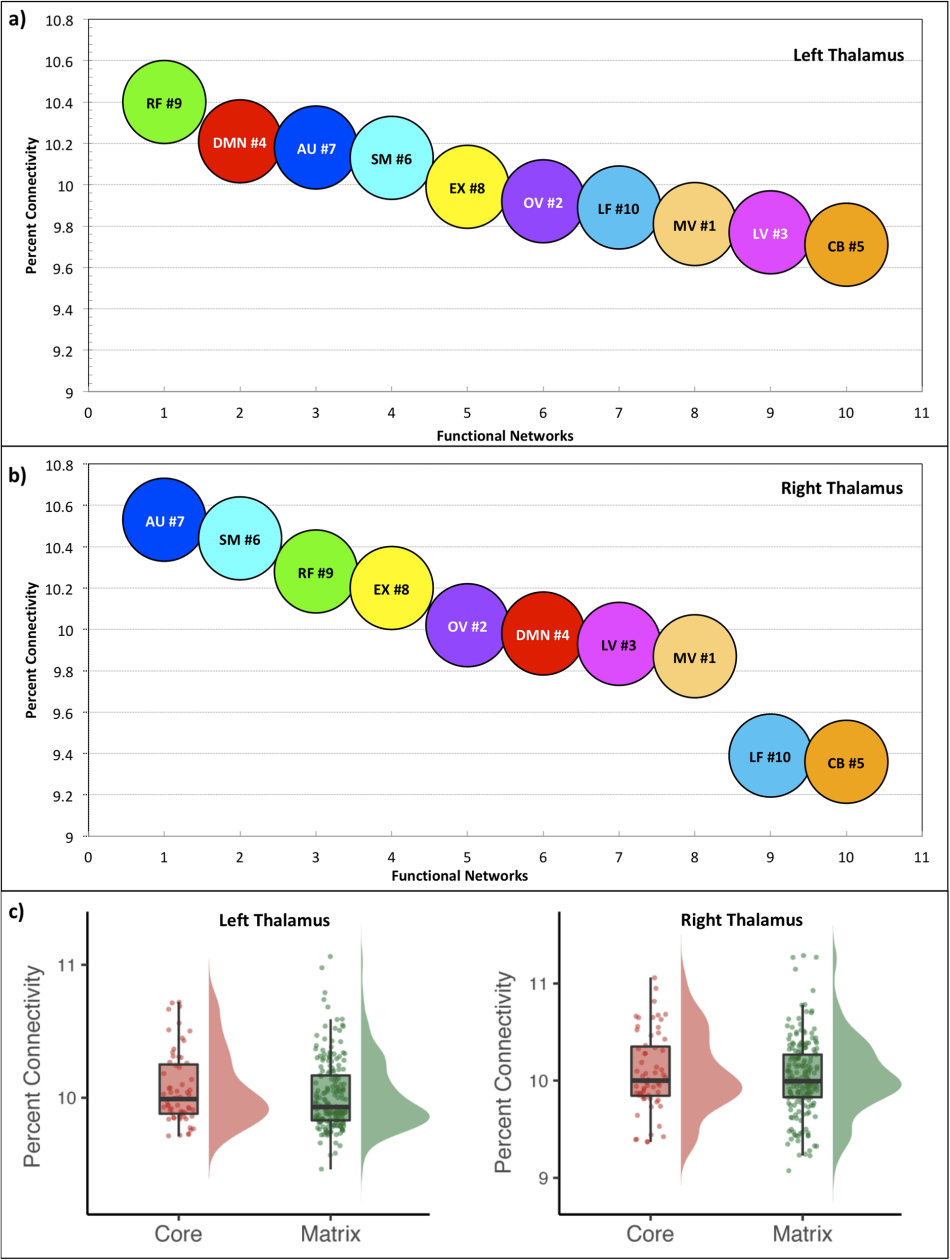
Ranking of network connectivity. **a**–**b** Depicted are the sum of percent contribution from all left and right thalamic nuclei within each cortical network sorted in descending order. Note: Right frontoparietal (RF) communicates strongest, while the cerebellum (CB) communicates least to the thalamus at rest. Interestingly, the three visual networks align almost next to each other. **c** Core and matrix nuclei percent connectivity of all functional networks: The raincloud plots show percent connectivity between the core and matrix nuclei values for all functional networks. Note: No significant differences exist between the groups. The raincloud takes input from the fixed effect maps of “*n* = 730 subjects.” The underline data are provided in the Supplementary Data 2 excel sheet. Box plot: left thalamus: quartiles (min 9.7100, lower quartile 9.8800, median 9.9900, upper quartile 10.2500, max 10.7200). right thalamus: quartiles (min 9.3700, lower quartile 9.8450, median 10.0000, upper quartile 10.3500, max 11.0600). The centerline in the box plot shows the medians, box limits indicate the 25th and 75th percentiles as determined by the R software, and whiskers extend 1.5 times the interquartile range from the 25th and 75th percentiles.

**Fig. 5:**
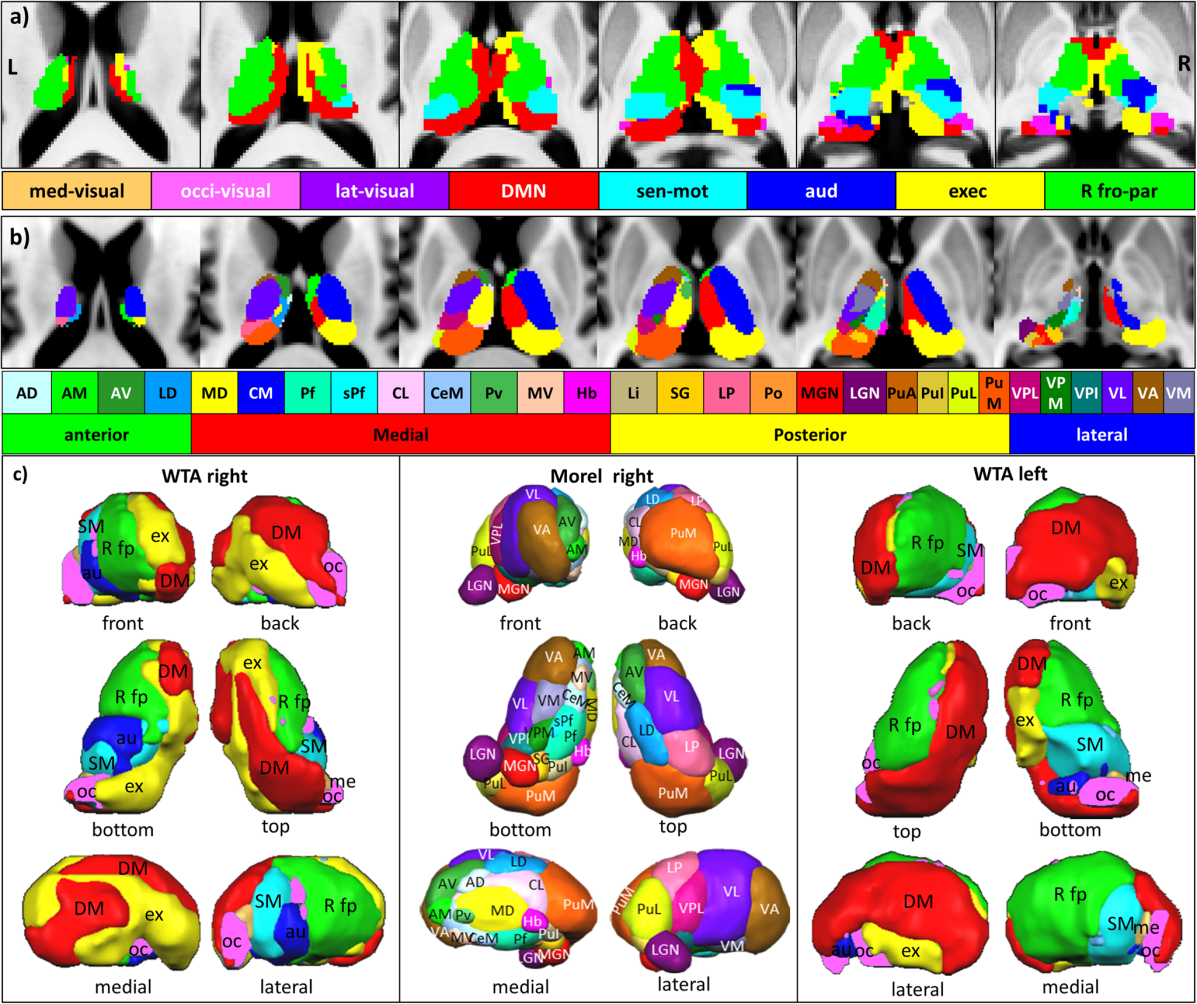
Winner-takes-it-all (WTA) maps. **a** WTA maps of the right and left thalamus on six axial slices and **b** corresponding slices from the histology atlas of the thalamus. **c** 3D rendered views of WTA maps of the left and right thalamus in comparison with right thalamic nuclei of the atlas of Morel. The networks are depicted in their different colors. The color assignments in hex color code: MV (FDCB6E), OV (FA70FC), LV (971B99), DMN (FA141B), SM (3CFEFE), AU (002CFB), EX (FFFC38), RF (41FB30), LF (6A971B). The nuclei depiction is color-coded with respect to each nucleus. The detailed color assignments in hex color code: AD (CBFFFF), AM (41FB30), AV (359430), LD (1AA0FC), MD (FFFC38), CM (002CFB), Pf (3FFDB6), sPf (3CFEFE), CL (FDCAFE), CeM (98C9FD), Pv (52B755), MV (FDC8AC), Hb (F933FC), Li (C0B47F), SG (FECE30), LP (FA6897), Po (FC963F), MGN (FA141B), LGN (711172), PuA (C56419), PuI (DCC642), PuL (DCFC36), PuM (FA571F), VPL (C2187B), VPM (1C7F13), VPI (177877), VL (612DFB), VA (965B15), VM (797AA6).

**Fig. 6:**
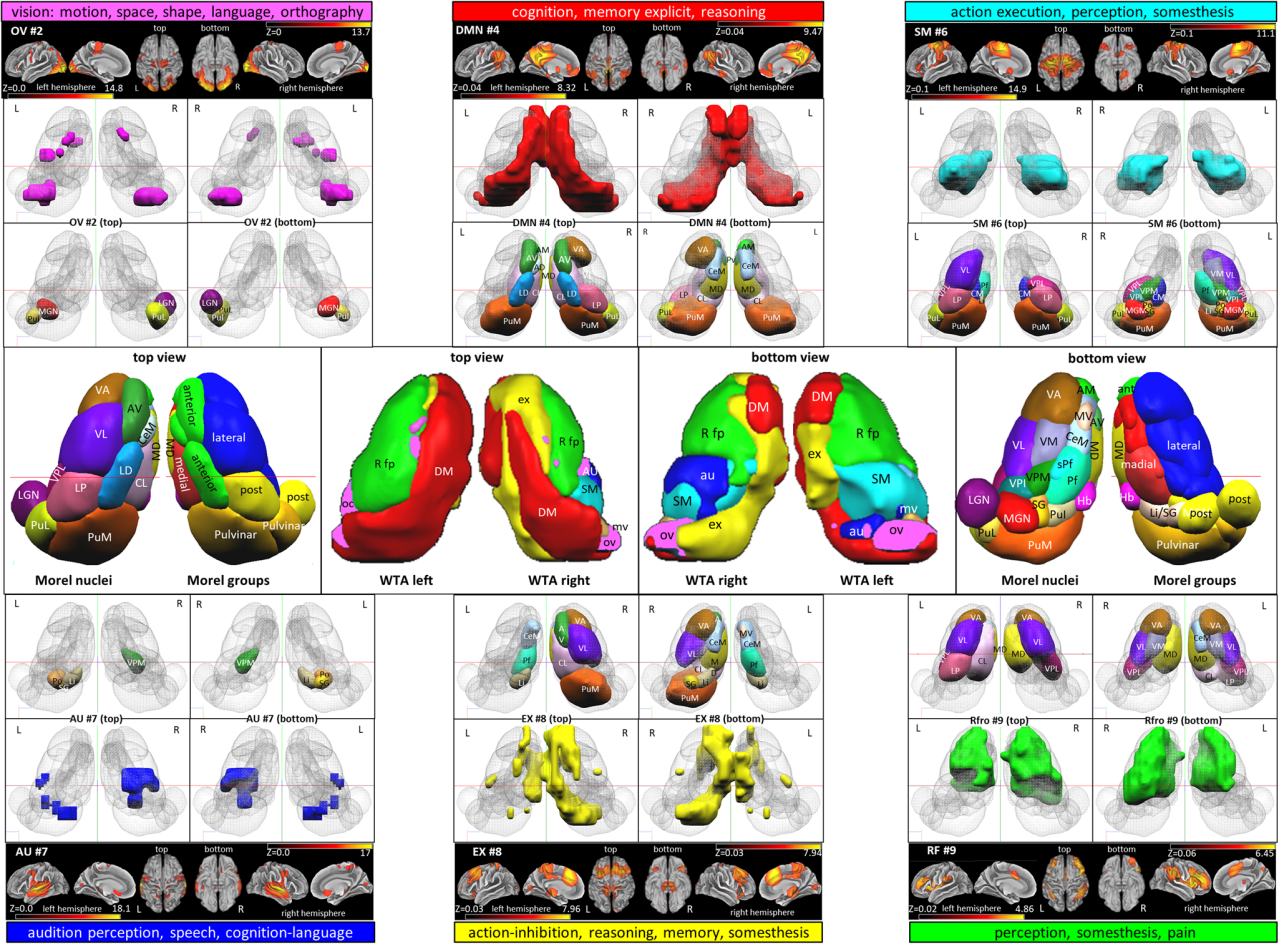
Comparison of 6 WTA maps with histologically defined thalamus nuclei. Top and bottom: the most apparent involvement of thalamic nuclei for six dominant RSN is depicted according to the Jülich Histology atlas. Surface views of cortical areas and their major functional assignments and corresponding thalamic nuclei exceeding ≥0. Two dice overlap (except for AU > 0.1) are shown. Middle: display of anatomy of thalamic nuclei according to Morel and WTA maps of the left and right hemispheres. The cortical maps’ color bar scales are also given in Fig. 1.

The 29 structurally distinct thalamic nuclei used for the connectivity analysis are given in Fig. 2 and Supplementary Table 5 with their abbreviated names. The following section describes the RSN connectivity with the different thalamic nuclei and nuclei groups (Figs. 3 and 4 and Supplementary Table 6). Furthermore, subsequently, the study depicts the strongest connectivity among all networks within the thalamus, i.e., winner-takes-all (WTA) (Figs. 5 and 6).

### Network-specific connectivity with thalamus nuclei

The visual networks comprise the medio-visual (MV), occipital-visual OV, and lateral-visual (LV) networks (Fig. 3a–c). MV reveals almost no major involvement as all anterior, intralaminar, and midline nuclei groups are below the overall mean. The only exception is a moderate involvement of the right LGN. The occipital-visual OV network shows no major participation of the anterior, medial, and midline nuclei but a remarkable bilateral suppression of Hb and the anatomically adjacent nuclei Li and SG. However, right LGN and bilaterally, the Inferior (PuI) and lateral pulvinar (PuL) nuclei are elevated. In the lateral-visual (LV) network, the anterior, intralaminar, and midline nuclei are almost similar to OV. However, the nuclei of the posterior and pulvinar group have vanished. Again, the epithalamic nuclei (Hb/Li/SG) are very low, and only a slight elevation is seen for the right premotor and motor nuclei VM, VA, and VL.

The Default mode network (DMN) overall reveals a left-sided dominance (Fig. 3d). An increased bilateral correlation exists for AD and LD in the anterior group, while the intralaminar nuclei CM, Pf, and sPf nuclei are low. The left Pv in the midline and LP in the posterior group are elevated. Like in OV and LV, the epithalamic nuclei (Hb/li/SG) are low. In the left pulvinar PuI, PuL, and PuM are above average, while no relevant involvement is found in the lateral group.

In the sensorimotor SM network (Fig. 3e), most nuclei are above average with a right-sided dominance. However, the left anterior nuclei are below average, and the left midline nuclei CeM, Pv, MV, and Hb are minimal. In contrast, especially the CM of the intralaminar nuclei is considerably elevated. In the posterior group, LP, Po, and MGN are dominant. In the pulvinar, only PuA is strongly bilaterally elevated. However, the highest connectivity arises from the lateral group’s right sensorimotor nuclei (VPL, VPM, and VPI).

The AU network (Fig. 3f) exhibits a clear right-sided dominance above average for all nuclei except for a bilateral low Hb, while the left-sided nuclei are below average.

The EX network (Fig. 3g) shows a right-sided dominance for all nuclei with a slight elevation of the intralaminar group. However, Hb and the adjacent nuclei Li and SG rise to a maximum exceeding all other nuclei. Other elevations above average are seen for Po and MGN in the posterior and PuM in the pulvinar group.

In the RF network (Fig. 3h), almost all nuclei are slightly above average, with an overall left-sided dominance. Elevations are found for LD, MD, and CL. LP and PuA are also slightly elevated in the posterior and pulvinar groups. All left nuclei except VPI are above the mean in the lateral group.

In contrast to RF, the LF network (Fig. 3i) shows no clear dominance as all nuclei are below the overall mean. The only remarkable detail is the higher correlation of all left-sided nuclei.

The CB network (Supplementary Fig. 4a/Table 6) shows the highest connectivity with the habenular nucleus.

Network-Specific Connectivity with Thalamic sub-groups- The average percent connectivity of all the nuclei within each thalamic group and subgroup shows gradual variable connectivity with functional networks (Supplementary Table 7). The right posterior, pulvinar, and lateral groups show the highest connectivity with SM and the lowest with the cerebellum. In contrast, the right midline as well as Li & SG nuclei revealed the highest connectivity to AU and lowest with the left frontoparietal network. The right LGN and MGN show the highest connectivity with the executive network and the lowest with the cerebellum network. In contrast, the intralaminar also shows the highest connectivity with the executive but the lowest to the frontoparietal-Left. Interestingly, the right DMN reveals the highest connectivity with the anterior group, while the right Hb shows the maximum connectivity with the cerebellum. Furthermore, laterality differences exist in the gradual modes of network-specific connectivity.

### Ranking of network-specific connectivity and core–matrix connectivity

A gradual connectivity analysis revealed a ranking of the thalamic functional networks (Fig. 4a, b and Supplementary Table 8). The relative difference shows only a tiny variation, i.e., approx. 10% each. The right frontoparietal network shows the highest connectivity in the left thalamus, while the cerebellum possesses the lowest. Remarkably, the three visual networks are almost aligned next to each other. Comparing both hemispheres depicts that specific connectivity differences exist, in which the auditory network dominates the right side.

#### Core and matrix connectivity

Although core and matrix nuclei of the thalamus are known to exhibit functional differences in their cortical interactions^33–35^, our comparison of these two nuclei groups could not reveal significant differences in their cortical connectivity (Fig. 4c and Supplementary Note 2).

### Winner-takes-all RSN correlation

While the percent connectivity analysis shows multiple affiliations of each voxel with all networks, the winner-take-all (WTA) analysis determines only the highest correlated network for each voxel (Fig. 5 and Supplementary Fig. 1). We excluded the cerebellum network in the WTA analysis due to its cortical overlap with the occipital lobe (Supplementary Table 1). However, even an inclusion of the cerebellum reveals an ordered representation in the WTA maps. The corresponding WTA maps show an ordered arrangement within the left and right thalamus (Supplementary Fig. 2). The most apparent nuclei involvements of six dominant networks are separately depicted in Fig. 6.

The MV area is small (1%) and confined to the LGN, PuI, and PuL (Supplementary Table 9**)**. In contrast, The OV network is more enhanced and bilaterally focused on the left MGN and right LGN in the posterior group. Especially the Inferior (PuI) and lateral pulvinar (PuL) nuclei are pronounced. However, the whole participation still comprises only 4% of the thalamic volume. The LV network reveals minimum residual connectivity (1%) for the left Li/SG and the posterior nucleus Po.

In the DMN network, the pattern is quite similar in both hemispheres except for the right VA and LP. The major connections arise from the anterior group, MD, and dominantly from the midline group’s CL, CeM, and Pv. According to the correlation maps, right LP in the posterior, PuL, and PuM in the pulvinar group survive. Other residual involvement is also found for the lateral group’s motor nuclei (VL, VA, and VM).

The SM network reflects the selective connectivity of intralaminar CM and Pf and all nuclei of the posterior group except for LGN. The pulvinar nuclei are bilaterally dominant, and in the lateral group, the three relay nuclei for somatosensory and viscerosensory afferents (VPL, VPM, and VPI) dominate on the left.

The AU network elucidates the minor thalamic involvement (≤5%), focusing on left Li and SG and bilaterally on Po and MGN. A second single involvement resides in the right sensorimotor nucleus VPM.

The EX network depicts a slight right-sided dominance for most nuclei. However, the dominant involvement in the correlation analysis of Hb, Li, and SG has vanished. The only minor surviving contributions arise from right Li/SG, Po, MGN, and PuM, accentuated from the motor nucleus VA.

The RF network shows a left-sided dominance, especially left MD, CL, CeM, and LP survived pointedly. However, the most striking is the prominent connectivity of all nuclei of the lateral group, except for VPI. Supplementary Fig. 1/Table 9 gives the detailed assignment of WTA maps.

### Hemispheric differences

**Fig. 7:**
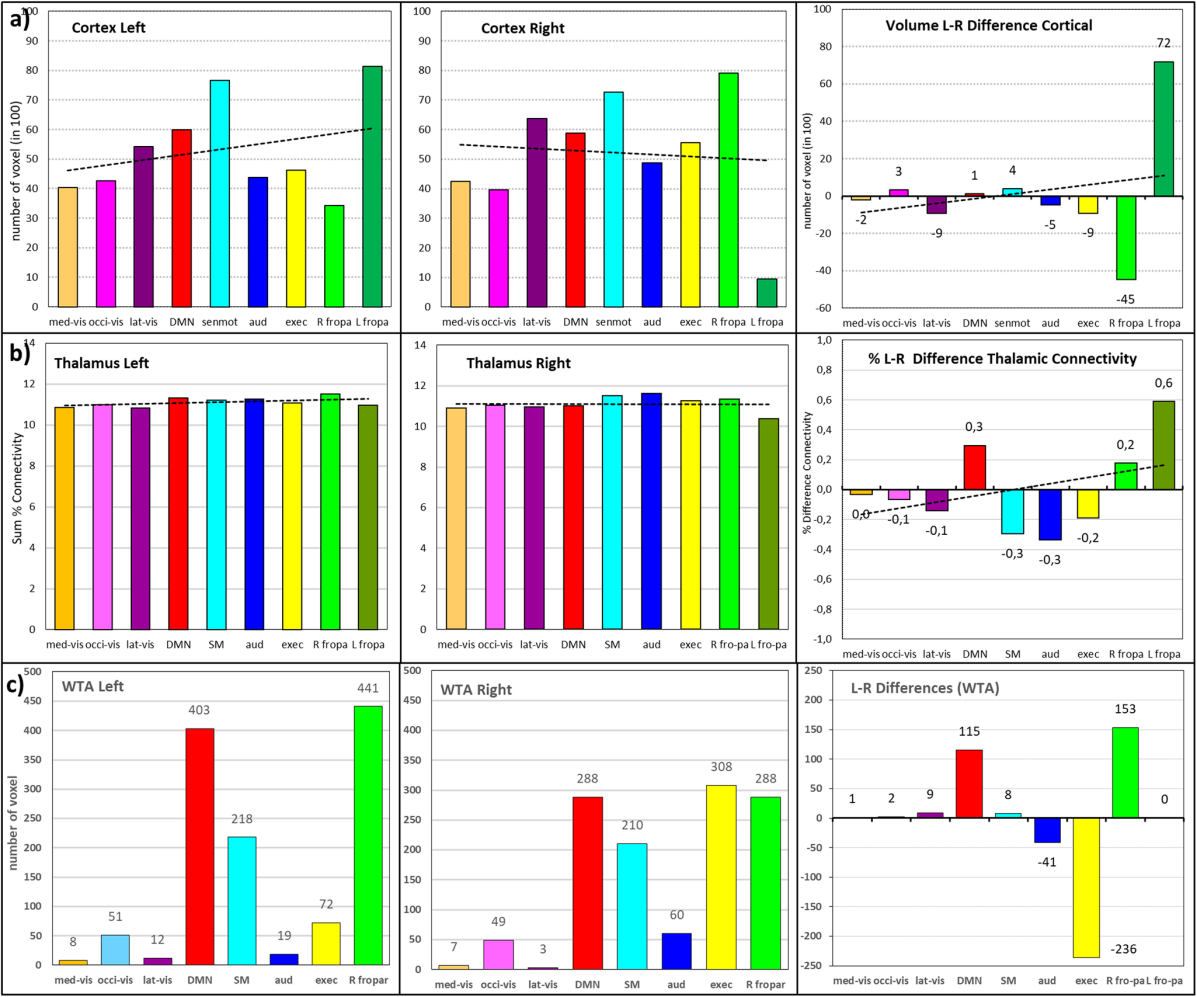
Hemispheric comparison. **a** Number of voxels and L–R difference of nine cortical RSN (number of voxels in 100); **b** Hemispheric distribution and L–R differences of thalamic connectivity in percent; **c** Max, mean and minimum of hemispheric distribution and L–R differences of connectivity of all thalamic nuclei.

The percent connectivity of the cortical RSN areas exhibits hemispheric differences, especially for the left and right frontoparietal cortical networks, compared to the rest (Fig. 7). A comparison of thalamic RSN clusters revealed that five also show pronounced hemispheric differences, especially for the DMN, the right frontoparietal network on the left, and the LVs, SM, AU, and EX network on the right (Fig. 7b). In contrast, the dice overlap differences revealed a right dominance for DMN and the frontoparietal network and a left dominance for AU and EX (Fig. 7c).

The percent connectivity and WTA map also exhibit hemispheric differences (Figs. 3a–i, 5, and 6 and Supplementary Fig. 1). The SM network reveals a pronounced engagement mainly of the right somatosensory nuclei VPL, VPM, and VPI in the lateral group and bilateral from the pulvinar, intralaminar and posterior group. In the AU network, mainly a slight right-sided engagement of the lateral nuclei is found. The RF and LF network possess slight left-sided dominance. The EX network exhibits right-sided dominance with extraordinarily high correlation values for the Hb, Li/SG nuclei. In addition, the recruitment of right-sided sensorimotor nuclei (VPL, VPM, and VPI) in the EX is remarkable. The major thalamic engagement is concentrated in the DMN, SM, AU, and EX networks, with an overall right-sided dominance. The DMN, in contrast, bilaterally engages the anterior nuclei, MD, the midline, and pulvinar group but less extended the intralaminar nuclei with a slight left-sided dominance.

### Behavioral relations using topic mapping

**Fig. 8:**
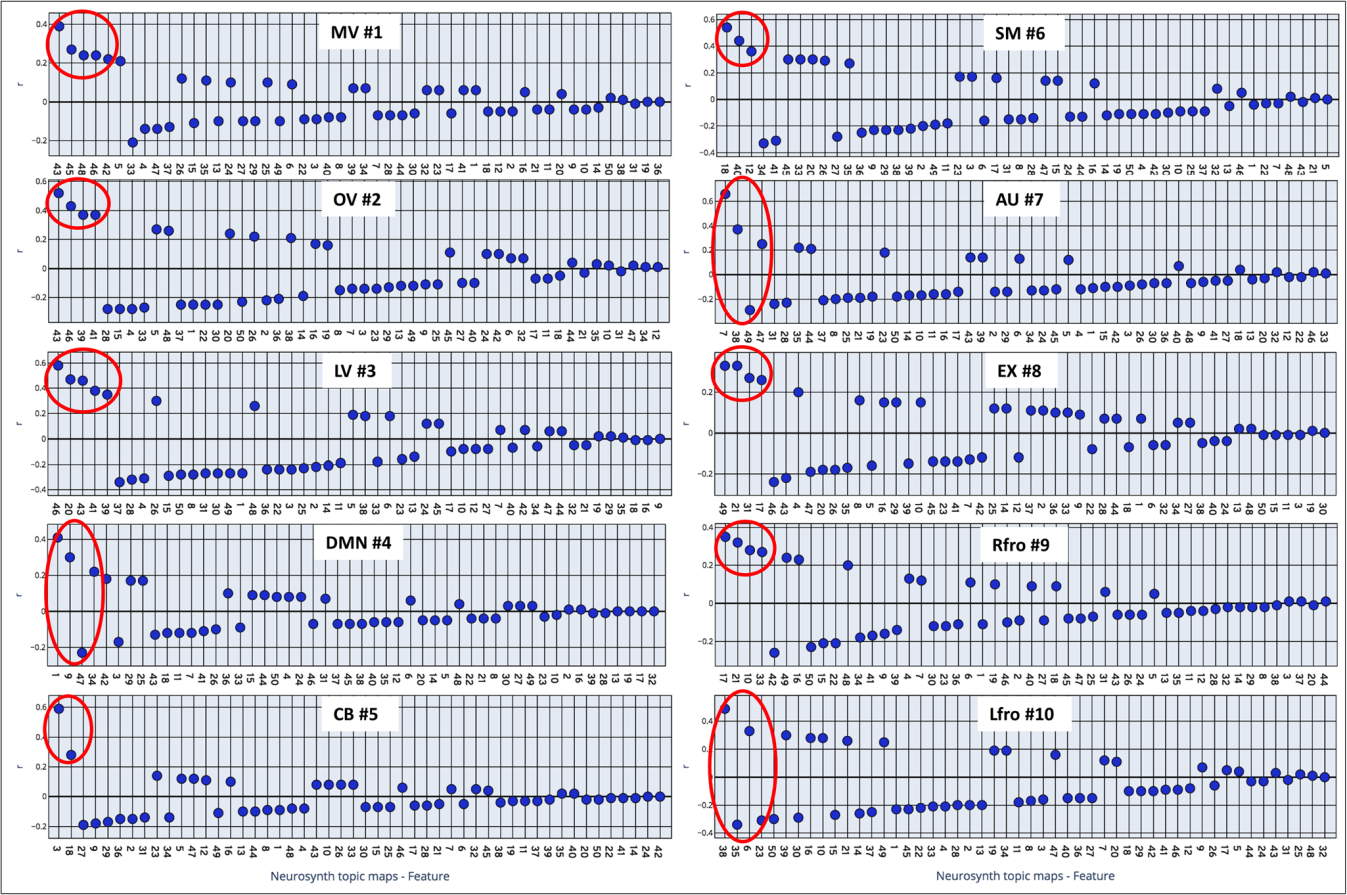
Large-scale functional networks associated with neurosynth topic maps. The neurosynth topic-based meta-analyses (https://www.neurosynth.org/analyses/topics/) using standard topic modeling approach (Latent Dirichlet allocation—train a correlation decoder) to the abstracts or text of articles in the database revealed 50 topic maps. Each subplot represents a correlation between LDA 50 topic maps and the corresponding functional network. The *X*-axis shows the numbered topic maps in each subplot. The related names are separately listed in Supplementary Table 10. The *Y*-axis in each sub-plot depicts the decoded correlation with Smith-10 large-scale functional networks. The red circles indicate the highly correlated topics above >+0.2. Figure 9 shows the topics within the red circles and their anatomical assignments within the thalamus.

**Fig. 9:**
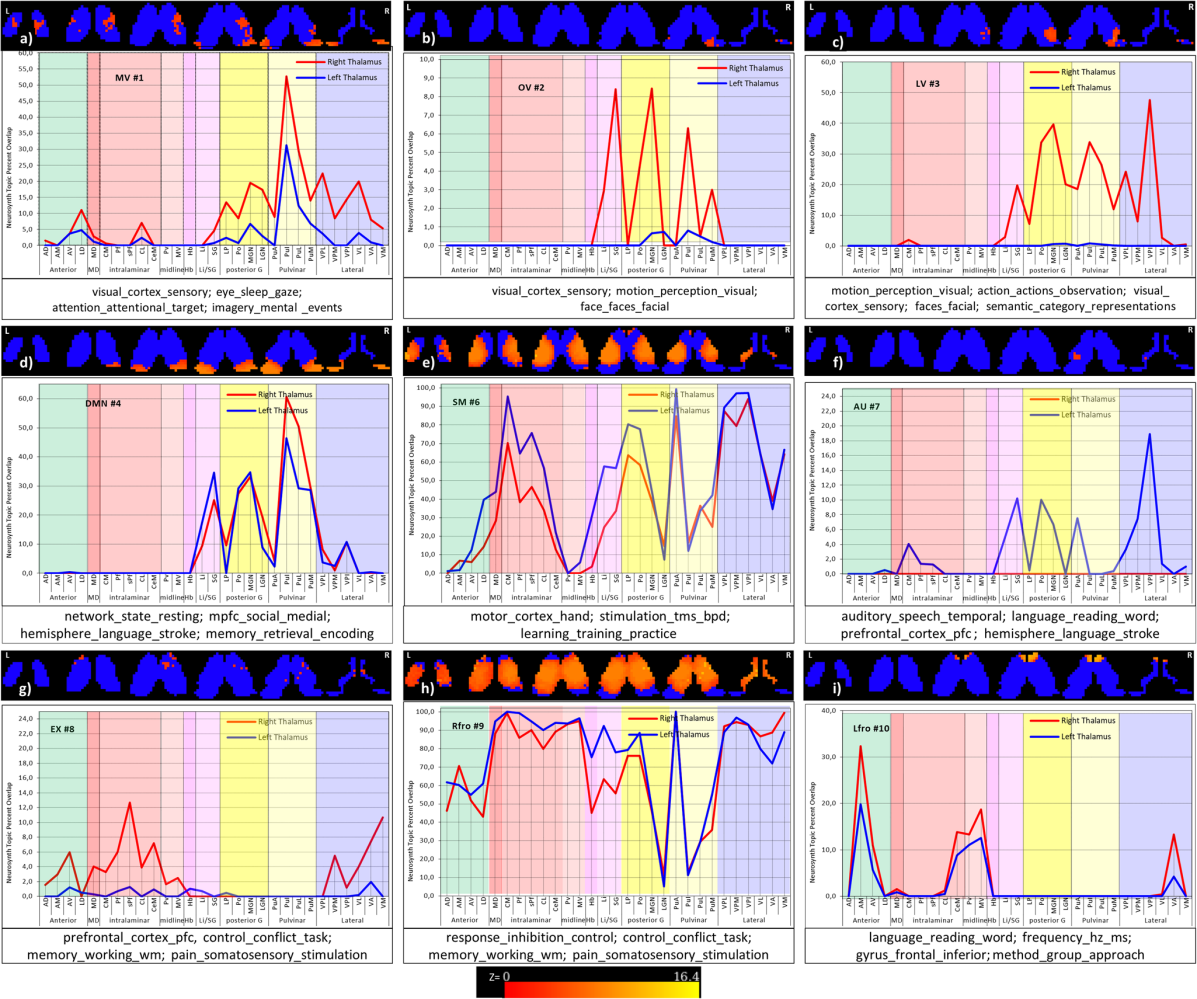
Large-scale functional network highly correlated neurosynth topic maps within thalamus: each subplot represents a functional network and its highly correlated topic maps (spatially overlaid on six different axial slices, depicting correlation maps with each network). The topic maps were thresholded at *z*-value 3.1 (*p* value 0.001). The graph within each subplot depicts the percent nuclei overlap of highly correlated topic maps (marked in the red circle in the figure) with the thalamus. Color scale: same for all the maps (0–16.4) across all the networks. The blue color depicts the thalamus mask in the background. **a** MV #1: 43 visual_cortex_sensory; 45 eye_sleep_gaze; 48 attention_attentional_target; 42 imagery_mental_events; **b** OV #2: 43 visual_cortex_sensory; 46 motion_perception_visual; 41 face_faces_facial; **c** LV #3: 46 motion_perception_visual; 20 action_actions_observation; 43 visual_cortex_sensory; 41 face_faces_facial; 39 semantic_category_representations; **d** DMN #4: 1 network_state_resting; 9 mpfc_social_medial; 47 hemisphere_language_stroke; 34 memory_retrieval_encoding; **e** SM #6: 18 motor_cortex_hand; 40 stimulation_tms_bpd; 12 learning_training_practice; **f** AU #7: 7 auditory_speech_temporal; 38 language_reading_word; 49 prefrontal_cortex_pfc; 47 hemisphere_language_stroke; **g** EX #8: 49 prefrontal_cortex_pfc, 21 control_conflict_task; 30_decision_making_risk, 16_response_inhibition_control; **h** Rfro #9: 17 response_inhibition_control; 21 control_conflict_task; 10 memory_working_wm; 33 pain_somatosensory_stimulation; **i** Lfro #10: 38 language_reading_word; 35 frequency_hz_ms; 6 gyrus_frontal_inferior; 23 method_group_approach.

The behavioral relation using topic mapping^36–38^ decoded Smith-10 functional network correlated topics (Fig. 8 and Supplementary Data 1) concerning their thalamic involvement. The topic maps are anatomically assigned to their corresponding thalamic groups and nuclei. The topic analysis also depicts hemispheric differences. The decoding reveals an intricate functional network-specific and overlapping association of topics, which could deliver an additional understanding of the reported behavioral association brain map (Supplementary Table 4 and Fig. 2 published in ref. ^12^). In addition, the topic maps are more precisely confined to specific cortical areas and can thus better depict an active thalamus-specific involvement (Fig. 9 and Supplementary Table 11).

The visual networks (MV, OV, LV) associated topic maps revealed overlap with the pulvinar and posterior group and the Li and SG nuclei. The MV and LV-associated topic maps also overlap with the lateral nuclei group. Among all three visual networks, MV topic maps overlap with the anterior nuclei (AV, LD) and slightly overlap with MD. The MV represents visual-cortex-sensory, eye-sleep-gaze, attention-attentional-target maps as well as imagery-mental-events topic maps. The OV represents visual-cortex-sensory, motion-perception-visual, and face-faces-facial topic-maps. The LV is associated with motion- perception-visual, action-actions-observation, visual-cortex-sensory, face-faces-facial, and semantic-category-representations. However, the involvement is mainly restricted to the right thalamus. All three visual networks show distinct and overlapping associations. For instance, the visual-cortex-sensory topic map represents all three visual networks. The OV and LV show also overlap with motion-perception-visual and face-faces-facial topics. The MV revealed distinct topic associations among all three visual networks with eye-sleep-gaze, attention-attentional-target, and imagery-mental-events topics. The OV appears to be a subset of the LV functional network-associated topics. In contrast to OV, LV shows distinct mapping with action-actions-observation and semantic-category-representations.

The DMN network nuclei, like the LV overlaps with Li, SG, posterior, pulvinar, and lateral nuclei groups but show completely different behavior with topic maps. DMN is now associated with mpfc-social-medial, hemisphere-language-stroke, and memory-retrieval-encoding topic maps. Moreover, the CB (Supplementary Fig. 4b) and SM functional networks (Fig. 9e) overlap with almost all thalamus nuclei. The only exception is a slight variation in the SM. However, the CB and SM overlap varies in both network-associated topic maps. The CB and SM both decoded motor-cortex-hand topic maps. However, the specific topic stimulation-tms-bpd and learning-training-practice only occur in the SM network, while within the CB network, the cerebellar-cerebellum-basal topic varies. The AU network-associated topic maps revealed thalamus overlap with the posterior group nuclei, including MGN, an auditory relay nucleus in the thalamus. In addition, lateral intralaminar, Li/SG, and PuA nuclei show an overlap. The AU highly correlated topics are auditory-speech-temporal, language-reading-word, prefrontal-cortex-pfc, and hemisphere-language-stroke.

Interestingly, EX network-associated topic maps avoiding the pulvinar and posterior groups show a slight overlap with the rest of the thalamus nuclei. The EX network’s highly correlated topics are the prefrontal-cortex-pfc, control-conflict-task, decision-making-risk, and response-inhibition-control. The Rfro and Lfro functional network correlated topic maps show a rather variable overlap with the thalamus and the topic maps. However, the Rfo displays an overlap with almost all the nuclei. In contrast, Lfro network topic-maps overlap with the anterior, intralaminar, midline as well as lateral nuclei groups. The Rfro topic maps are response-inhibition-control, control-conflict-task, memory-working-wm, and pain-somatosensory-stimulation. In contrast, Lfro topic-maps are the language-reading-word and frequency-hz-ms. In contrast to the combined topic maps, separate topic maps present a better impression of associated mental function to the corresponding thalamus nuclei (Supplementary Figs. 10–19 and Supplementary Note 3).

## Discussion

The cytoarchitectonic characterization of the RSN shows specific and overlapping cortical areas, indicating the functional multiplicity of cortical areas. The results link the functional connectivity of behaviorally defined cortical RSN with histologically defined thalamic nuclei. Moreover, all cortical networks show connectivity with the thalamus with a considerable variation (Supplementary Fig. 3). The frontoparietal network shows the highest connectivity with the thalamus, and the cerebellum shows the least. The sensorimotor, executive, and default-mode networks also show higher connectivity than the remaining RSN.

Furthermore, the analysis shows hemispheric differences in connectivity and topic analysis. The following text describes the relevance of cortico-thalamic connectivity in the first section. The subsequent sections discuss the alignment of the RSN-thalamus connectivity of nuclei, nuclei-groups, and the whole thalamus with the literature. The last section finally discusses the observed findings of the topic analysis. A comprehensive comparison of all findings is summarized in Supplementary Fig. 5.

The thalamus acts as a central core to enable ongoing cortical functioning. The cortical sheet’s recurrent cortical dynamics are driven through the thalamus also during rest^6^. In addition, the different cortical areas underlining RSN may communicate via *trans*-thalamic pathways to maintain the ongoing activity^39^.

The nuclei-specific functional connectivity with each network renders a detailed insight into the selective thalamic contributions of each network. The functional assignment of medial visual networks MV is dedicated to the perception of objects’ motion, shape, and space. However, this is not reflected in the correlation values and WTA maps, revealing that the thalamic recruitment of MV is minimal under fixed open eyes conditions.

In OV, the slightly accentuated bilateral involvement of the pulvinar nuclei (Pul and PuL) is congruent with findings that most of the input to the extrastriate areas V2, V3, and V4 comes from the pulvinar complex. Moreover, in contrast to MV, the diminished correlation of Hb may indicate that bottom-up information from midbrain areas and brainstem is suppressed in OV.

In LV, increased involvement of cortical areas in the IPL, the pericentral, and frontal regions has occurred. An underlying reason is the growth of cortico-cortical connectivity of parietal areas, as shown by several anatomical and imaging studies^40–42^, permitting complex visual processing related to the cognition of language, orthography, and sensorimotor processing^43^. Nevertheless, the pulvinar’s contribution has completely vanished in the thalamus compared to OV.

The DMN is mainly active during rest and associated with wakeful rest^44^, daydreaming, episodic memory, and other cognitive processes^44,45^; all these functions correspond with the reported behavioral domains of cognition-memory-explicit and cognition-reasoning^12^. In analogy to cortical areas, the correlation maps enclose nuclei in the anterior, midline, posterior, and pulvinar groups; however, sparing most intralaminar nuclei. Especially AD, AV, LD, MD, CL, Pv, LP, PuL, and PuM are accentuated. Functionally the correlation of the anterior nuclei group is explained as these nuclei are associated with the limbic system and mainly include the hippocampal–diencephalic and parahippocampal–retrosplenial network^46,47^. Hereby encircle AM, AV, and LD the temporo-amygdala–orbitofrontal network, which integrates visceral sensation and emotion with semantic memory and behavior.

The consistent involvement of CL—the largest intralaminar nucleus—is probably due to its projection to the superior colliculus (SC), linking to areas concerned with eye movements, visual function, and awareness^48^. The involvement of CL in the DMN has already been noted^49^, and it has been suspected that CL promotes the re-emergence of consciousness via its connection with the ventral tegmental areas (VTA).

Finally, the LP elevation and the high correlation of PuI and PuL reflect connections with visual areas in the parietal lobe (SPL, IPL, IPS)^50,51^. A similar correspondence can be suspected for PuM, reflecting reciprocal connectivity with the superior temporal gyrus, cingulate cortex, amygdala, and insula.

The SM is involved in motor action and execution, somatosensory perception, somesthesis, and pain perception. The cortico-thalamic connections involve nuclei of the intralaminar, posterior, pulvinar, and lateral groups but are less extended than the midline nuclei. Especially CM, Li/SG, the posterior nuclei, PuA, and the sensorimotor nuclei VPL, VM, and VPI are dominant. Hereby, the elevation of the intralaminar CM is remarkable as CM belongs together with the parafasciculus nucleus Pf to the truncothalamic nuclei centromedian-parafascicular nuclei complex (CM-Pf). The intralaminar thalamic nuclei are the prototypic thalamic projection system with major connections joining the cerebral cortex via the basal ganglia^52^. Specifically, CM projects the central and lateral parts of the globus pallidus externus, the globus pallidus internus, the substantia nigra, and the subthalamic nucleus^53^.

The correlation between Li/SG, Po, and MGN can be related to sensorimotor processing and visual-motor coordination. SG is often seen together with Li as a single limitans-supra-geniculate complex (Li/SG)^32,52^ as both receive spinothalamic fibers from the SC, extending them to the ventral posterior complex (VP), and projects to the caudate nucleus^54–56^. The posterior nucleus Po, located at the dorsolateral aspect of SG, seems to constitute part of the somatosensory thalamus^32,52^. The contribution of the anterior pulvinar nucleus (PuA) might be caused by the processing of somatosensory information^57^ from the postcentral somatosensory parietal lobe.

Finally, the involvement of VPL, VPM, and VPI adequately reflect their participation in the SM network, as the ventral posterolateral nucleus (VPL) receives somatosensory inputs from the trunk, extremities, and head, the ventroposterior nucleus (VPM) from ascending trigeminal afferents, and the posteriorinferior nucleus (VPI) fibers from the superior cerebellar peduncle. Similarly, it complies with the absence of the motor nuclei VA and VM^58^ with the resting state.

Although the cortical areas of the AU network encircle the major bilateral areas of the temporal lobe, the thalamic correlation maps are, on average, rather unsuspicious. Although, the slight right-sided dominance for all nuclei is decreased in the WTA map so that only remnants of Li/SG, MGN, and right sensorimotor nuclei (VPL, VPM, and VPI) remain. In addition, similar to the SM, it displays AU, remarkable suppression of the Hb.

The EX network comprising the bilateral dorsolateral prefrontal cortex (DLPFC) and posterior parietal cortex (PPC) serves as a core hub for the maintenance and manipulation of information and decision-making in goal-directed behavior^59^. It is engaged during cognitive tasks requiring external attention, such as working memory, relational integration, response inhibition, task-set switching, and several creative thought processes^60^.

Contrary to the almost bilateral involvement of cortical areas, the thalamus exhibits a slightly right-sided dominance, in which intralaminar nuclei, right Po, MGN, and PuM are elevated. However, the dominant nuclei are right Hb and LI/SG, reaching a maximum correlation, although almost absent in the WTA map, while the right motor nuclei VA and VL^58^ survive. The functional interpretation remains that the strong and selective engagement of the right Hb and the adjacent Li/SG nuclei in the correlation maps is challenging. Anatomically the Hb splits into two subregions in mammals: the medial and lateral habenula. The lateral habenula is hereby a source of negative reward-related signals in dopamine neurons^61^, as it connects the septum, hypothalamus, basal forebrain, globus pallidus, and prefrontal cortex with the dopaminergic, serotonergic, and noradrenergic systems^62^. Therefore, it is not surprising that the Hb is part of the EX network and is involved in the behavioral responses to pain, stress, anxiety, sleep, and reward, and its dysfunction is associated with depression, schizophrenia, and drug-induced psychosis^63,64^. By inhibiting dopamine-releasing neurons, Hb activation also suppresses motor behavior^65^. Thus, as a highly conserved structure in the brain, the Hb provides a fundamental mechanism for survival and decision-making. However, the right sided-dominance and the Hb disappearance in the WTA map can only partly be explained by its relatively small volume (26 voxels), which could face an overlap with other nuclei. It may also be due to the thalamic template of the Morel atlas, which uses identical bilateral templates.

The RF network includes the right parietal and frontal but also temporal areas as well as parts of the right supplementary motor cortex. It contains an active network across various tasks, including sustained attention^66^, response inhibition^67^, and arousal regulation^68^, all involving right-lateralized cortical networks, suggesting that these regions subserve more general cognitive functions^69^.

However, the thalamic participation of the RF is slightly left dominant but relatively equally distributed over all nuclei and exhibits no major outliers. While the WTA map reveals a different distribution, as predominantly left MD, CL, LP, and all lateral nuclei except for VPI are bilaterally dominant.

However, the minimal involvement of nuclei in conjunction with a slight left-sided dominance indicates that most processing is performed between different cortical areas and the hemispheres without thalamic involvement. Nevertheless, the slightly enhanced correlation of left MD and CL can be explained as both are anatomically and functionally linked to areas concerned with eye movements, visual function, and awareness. Specifically, MD recognizes memory and regulating cortical networks, particularly when ongoing activity maintenance and temporal extension in frontal lobe areas are required^70,71^. Similarly, CL has intrinsic connectivity to the anterior thalamic nuclei and receives input from the brainstem reticular activating system.

The LF network predominantly comprises parietal, temporal, and frontal areas in the left hemisphere. However, in contrast to the RF network, a parietal shift occurred from the IPL and IPS to the SPL and frontal areas. Functionally the LF network is most strongly linked to relational thinking^72^ and has been associated with language comprehension as decreased LF connectivity in stroke patients was associated with the impairment of language function in their comprehension ability^73^. Corresponding to the left-sided cortical dominance, the thalamic correlation shows a left-sided dominance without prominent outliers. The relatively homogeneous correlation indicates that the communication within the left frontoparietal network is mainly maintained by ipsilateral cortico-cortical processing without thalamic participation. Therefore, in the WTA map, no substantial contribution remains.

The major thalamus group-specific functional connectivity with each network renders a detailed insight into the thalamic group-specific contributions of each network (Supplementary Table 7). The anterior group’s highest connectivity with the DMN indicates known memory and attention-specific contributions^74,75^. The LGN and MGN are primarily the relay nuclei with the thalamus and interestingly exhibited the highest connectivity with the executive network, which aligns with the suggested higher-order functions of the LGN^76^ and MGN in human cognition^77^. The executive network also shows the highest connectivity with the intralaminar, which aligns with the known higher-order functions and anatomical connectivity studies^78–80^. The SM network exhibits the highest connectivity with the posterior, pulvinar, and lateral groups at rest^32,81^. This may be, among other reasons, due to the cortico-cortical interactions required to maintain the ongoing activity^82–84^. The recurrent cortical dynamics maintained via thalamic nuclei and trans-thalamic pathways may be the basis behind the higher connectivity between Li/SG, midline group to the auditory network at rest. However, much work is anticipated to link the suggested hypothesis and speculations in the discussion to forge a clear mechanistic and causal role of cortico-thalamic interactions.

The ranking of network connectivity shows variable thalamus engagement with the networks (Fig. 4). The right frontoparietal network shows the highest connectivity with the thalamus at rest. Both frontoparietal networks are suggested to play a role in high-level cognition and adaptive behavioral tasks, i.e., working memory, reasoning, set-shifting, response inhibition, selection attention, and problem-solving^85^. The three visual networks align almost together, suggesting similar cortico-thalamic connectivity under eyes-open conditions. The cerebellum shows the least connectivity at rest, possibly indicating its inferior role during rest. However, similar investigations are needed in task-fMRI examinations to better interpret the network-specific communication to the thalamus.

The relation of behavioral mapping using topic-analysis revealed distinct correlations, which are partly associated with identical thalamus nuclei, suggesting the thalamus’s various behavioral and functional aspects (Fig. 9 and Supplementary Figs. 10–19).

However, the CB, SM, and Rfro functional networks overlap with similar nuclei to a varying spatial extent, with almost network-specific topic maps. The three visual networks possess behavioral-specific processing but overlap with posterior and pulvinar nuclei groups. More specifically, the pulvinar nuclei group is implicated in facilitating visual processing in the cortex^1,31,86^. However, the topic mapping shows its overlap with topics of Rfro, SM, CB, DMN, and all three visual networks. This finding suggests that the pulvinar nuclei possess a more prominent behavioral role than previously noted^87,88^. However, recent evidence^89–91^ and connectivity of pulvinar with parietal and frontal areas^86^ suggest the validity of our topic analysis.

Furthermore, relay-specific thalamus nuclei are not only associated with relay-specific behavioral topic maps but also with diverse higher-order mental tasks. For instance, the Rfro network overlaps with posterior and lateral group nuclei overlap and is associated with control-conflict-task, memory-working-wm, and response-inhibition-control topic maps. These topic-based observations suggest that the thalamus works in analogy to a computer, where many software with different functional content can operate by utilizing the same processor components.

The neurosynth topic-mapping analysis relies on available cortico-functional maps published in 2009 by Smith et al.^12^. Therefore, the mental function mapping with the neurosynth topic-mapping analysis offers a supplementary understanding of the cortico-thalamic behavioral association. In summary, the study adds to the current knowledge of cortico-thalamic interactions by adding the insight that some topics share identical nuclei. Furthermore, the analysis also indicates hemispheric laterality differences.

The comparison of different cortical RSN correlated thalamus maps, percent connectivity, winner-takes-it-all, and associated topic maps revealed a rough correspondence, as well as some specific differences (Supplementary Figs. 5–9).

The observations in the study remain tentative due to limitations. The considerable overlap of most RSN, especially in the occipital parietal and frontal areas, causes a substantial overlap of thalamic activity and diminishes the specificity of the obtained correlation values. Second, the assignment of the cortical areas of the RSN using available software atlases in FSL is limited as (i) it relies on a limited sample of 36 subjects, (ii) a separate salience network does not exist, and (iii) the cerebellum RSN overlaps slightly with visual cortices, (iv) the JHA does not include major parts of the frontal and inferior temporal lobe.

Regarding anatomy, the atlas of thalamic nuclei is based on six maps derived from stacks of histologically processed brain sections by combining three different series of the right and left hemispheres to construct a unique three-dimensional surface rendered model of 29 major thalamic nuclei^92^. Therefore, the template of each nucleus is applied to both hemispheres and can therefore not be seen as a representative sample for a larger population^93–95^. Due to the major variation in size of the nuclei (min: MV: 14 and PuA: 376 voxel) and the suspected laterality differences, the proper assignment of each nucleus to the cortical networks is limited and an unresolved source of errors. In addition, it remains unclear whether the histologically defined templates reflect functional properties of cortical-thalamic connectivity as most nuclei possess intense internal connections and may be even be further segregated in different functional and/or smaller domains.

Like age and gender, a person’s motor, sensory, limbic, cognitive, and possibly many other variables may shape thalamic nuclei configuration. The use of the morel-atlas ignores this intrinsic variability concerning laterality as inter-individual differences. Therefore, this limits the quality of observations made on the level of each thalamic nuclei. Although a segmentation method like THOMAS^96^ utilizes specialized MRI sequences to segment thalamus nuclei, this refined acquisition scheme is unavailable in the HCP data set. The HCP structural images depict nuclei boundaries as more continuum than the WM nulled MPRAGE images shown at 7 or 3 T^96^. Replacing the presented analysis in the manuscript with THOMAS^96^ could better illustrate inter-individual thalamic anatomy and thus result in a refined cortico-thalamic connectivity pattern. Therefore, THOMAS^96^ remains a meaningful choice for future study.

Finally, evaluating cortico-thalamic connectivity differences at rest and their assignment to thalamic nuclei represents a simple and somewhat crude approach to infer their specific role in concert with cortical RSN. Furthermore, the study did not evaluate inter-individual differences. There are unrevealed treasures of information underlining and respecting the inter-individual variability, which should be further pursued as a research endeavor.

Ultimately, the functional correlation does not imply causation^97^. Because the rsfMRI measurements only probe the low-dimensional signals in contrast to the immensity of the communication potential in the brain^97^. Therefore, causal clarity is compromised. There may be information about causality in this functional connectivity, but not a causal reality due to the reliance on the low-dimensional rsfMRI measurements. However, investigation of ongoing activity, i.e., resting-state signals using correlation analysis, plays an essential role in determining cortical functions and thus should not be ignored.

## Methods

### Data

The data were selected from the HCP dataset^98^, and 730 datasets were chosen, which went through the full MRI acquisition pipeline of structural, 4 resting states, and 7 tasks sessions (see imaging protocol: https://protocols.humanconnectome.org/HCP/3T/imaging-protocols.html). The 730 subjects comprised 329 male and 401 female subjects aged 22–37 (693 right- and 37 left-handed).

HCP acquired informed consent from the participants. Furthermore, the participants were allowed to share their data over the Internet, i.e., ConnectomeDB, for use by other scientists or the general public with a code number^98,99^. The code number allows for maintaining their privacy.

### Data analysis

**Fig. 10:**
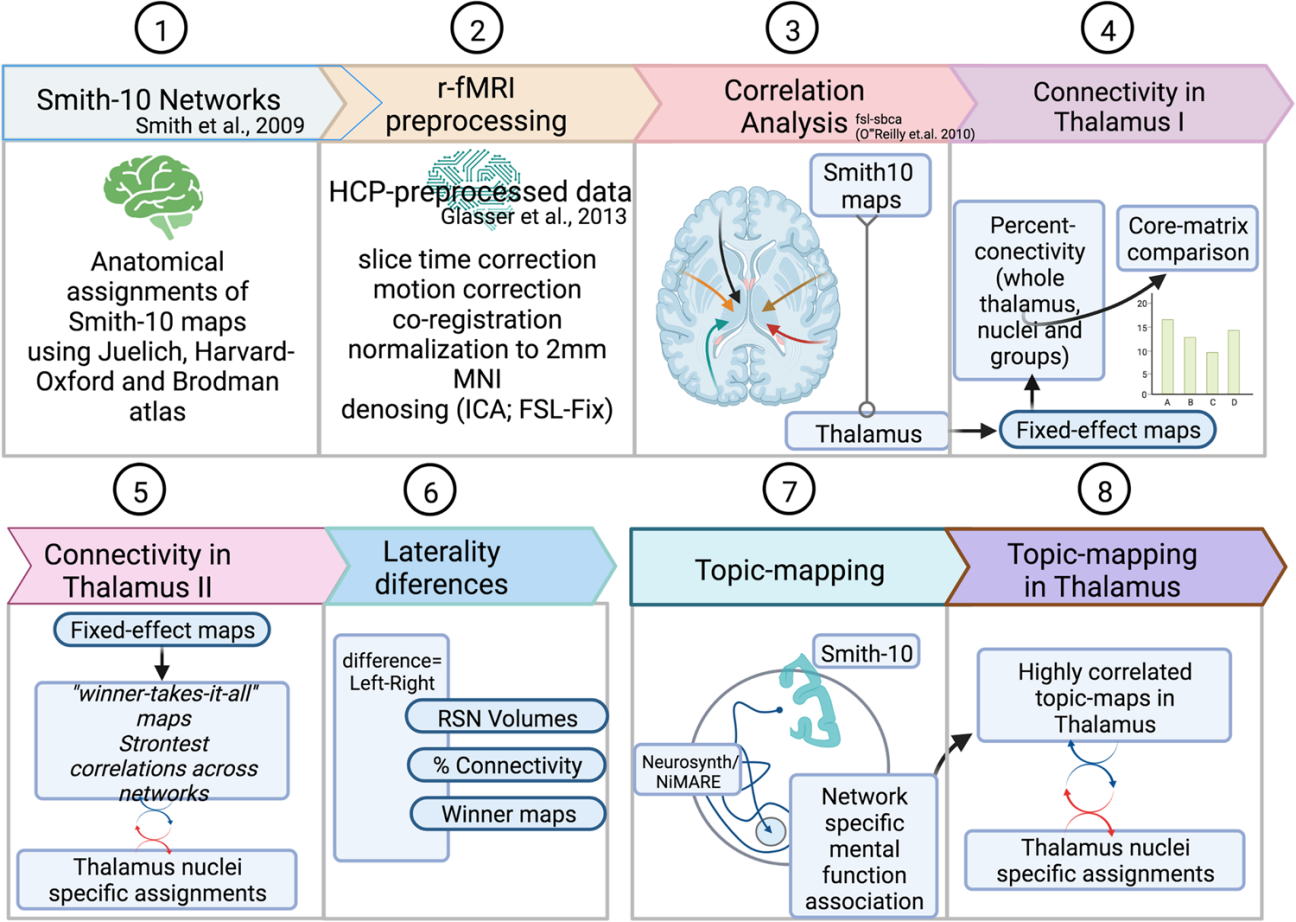
Analysis workflow. Workflow summarized according to the performed steps. (1) The first analysis relates to the anatomical assignments of the Smith-10 maps. (2–6) Correlation analysis between large-scale functional networks (Smith-10) and thalamus. 2: The mentioned preprocessing steps outline the major steps in the graphical illustration. The HCP minimal processing pipeline includes a more detailed overview of the preprocessing workflow (see Figs. 7 and 8 in Glasser et al.^110^), topic analysis of Smith-10, and visualizing highly correlated topics within the thalamus. Figures are designed using biorender.

The flow chart depicts major processing steps (Fig. 10). The data analysis first investigates the anatomical assignments of the Smith-10 maps, with three available atlases. Afterward, Smith-10 maps are used to investigate the connectivity with the thalamus. The thalamus connectivity analysis depicts fixed-effect maps and percent connectivity analysis with the whole thalamus, nuclei groups, and nuclei. In addition, the percent connectivity of the core-matrix nuclei was analyzed. Furthermore, the strongest connectivity was explored using the winner-takes-it-all approach. Finally, a separate analysis decodes network-specific association with neurosynth topic maps.

### Anatomical assignments of Smith-10-cortical brain maps

We computed the anatomical assignments of 10 brain maps by performing percent overlap analysis with Juelich histological atlas (JHA)^100–102^, Harvard-Oxford cortical atlas (HOA)^103–106^, and Brodman atlas (BAA). We took JHA and HOA labels from the FSL atlas directory^107^. The BAA was taken from the micron template directory^108^. The matching atlas templates were taken for the percent overlap analysis. The brain maps were thresholded at 0.2 using fslmaths to remove widespread spurious correlations. The JHA labels were extracted using the maximum probability atlas labels. The percent overlap analysis accounts for the percent overlap of the atlas labels with the Smith-10 brain maps. Thus, the presented overlap represents the percentage overlap of JHA, HOA, and BAA labels (Supplementary Tables 1–3) with every Smith-10 map. At first, the analysis determined the overlap between each Smith-10 map with the atlas labels. In the next step, the percent overlap was determined by dividing the number of overlapping voxels within the label by the total number of voxels in the atlas label.

### Cortical brain map visualization

The RSN cortical volume maps were thresholded (<2) and mapped to the Conte69 32 K surface atlas^109^ to the left and right hemispheres. The sum of vertices volume was computed using the connectome workbench. The connectivity analysis relies on the volume space. The surface maps were calculated only for the RSN visualization for better depiction.

### Connectivity analysis

The 10 cortical resting state maps were obtained from the Smith-10 cortical functional network work^12^ (https://www.fmrib.ox.ac.uk/datasets/brainmap+rsns).

### rsfMRI preprocessing

Preprocessed and ICA denoised data were taken from the HCP database using FSL-Fix. The preprocessing pipeline is discussed in the glasser paper^110^. The noise in the HCP data, i.e., effects of motion, non-neuronal physiology, scanner artifacts, and other nuisance sources, were removed using a machine learning-based classifier, i.e., FSL-Fix^111,112^. Data were smoothed with a 3.5 mm Gaussian kernel using fslmaths.

### Cortical–thalamus correlation analysis

The Smith-10 cortical maps were thresholded at 0.2 and separated for each brain hemisphere to determine their ipsilateral cortico-thalamic correlations. The FSL fMRI Resting State Seed-based Connectivity (FSL-SBCA)^113^ was used to compute partial correlations from ipsilateral cortical resting state networks to the ipsilateral thalamus separately for each session for each subject in the rfMRI datasets.

### Fixed effect analysis

Correlation analysis resulted in a separate map for each RSN in each of the four rsfMRI sessions in each subject. Each voxel represented the correlation value for the respective cortical network. The correlation for all the networks was summed, and then each single network map was divided by the sum of all the maps resulting in normalized maps. Furthermore, a fixed effect analysis across subjects was performed.

### Quantification of correlation maps

Each normalized 4D group map contained ten values for every voxel representing a relative contribution to each network. We summed the values within every thalamic nucleus for each cortical network. The sum of each nucleus was divided by the total sum for the same nuclei across all the networks. The resulting ratio (multiplied by 100) inferred the percent communication contribution of each thalamic nucleus to each cortical network during rest. The nuclei-specific percent connectivity values were further grouped to determine their nuclei-group-specific percent connectivity. Furthermore, the whole thalamus percent connectivity was determined for each functional network by first calculating the network-specific sum within the whole thalamus mask and dividing by the sum over all the networks, followed by multiplying by 100.

### Winner map analysis

The strongest correlations at each voxel were computed using the WTA approach on the fixed effect group maps. Hemispheric difference analysis was performed by subtracting each right winner cluster from each left winner cluster.

Histological correspondence analysis: Dice overlap The WTA cluster’s spatial assignments were determined to allocate their underline thalamic nuclei^92^.

### Comparison of core and matrix nuclei

The percent connectivity values of each nucleus (Supplementary Table 4) were clustered into their corresponding core and matrix nuclei groups. In the next step, a statistical test was performed to determine the difference between all the percent connectivity values in the core and matrix group using R, i.e., i) two-sample t-test: t.test(core, matrix), ii) Wilcoxon rank-sum test: Wilcox.test(core, matrix, paired = FALSE, alternative = “two.sided”). See detailed results and discussion in Supplementary Note 2.

### Topic-based meta-analysis and Smith-10 large-scale functional networks

The topic-based neurosynth meta-analysis^36,37^ was done using NiMARE python package^114^. The NiMARE python workflow first downloaded the LDA50 neurosynth dataset. The second step converted the downloaded dataset to the NiMARE dataset file. In the third step, the correlation decoder was trained. In the fourth step, the Smith-10 large-scale functional network was decoded (Fig. 8 and Supplementary Data 1).

The highly correlated topic maps (*p* 0.001) within each Smith-10 functional network were combined (>±0.2 correlation/*r*) to depict thalamus-specific contribution (Fig. 9). In the final step, the thalamic-specific nuclei contribution was assigned within thalamus^92^ by computing the percent overlap (Fig. 9 and Supplementary Table 11). In addition, the separate depiction of each topic map reveals detailed topic-specific associations with the thalamus (Supplementary Figs. 10–19/Table 12/Note 3).

### Statistics and reproducibility

Correlation analysis resulted in a separate map for each RSN in each of the four rsfMRI sessions in each subject. Each voxel represented the correlation value for the respective cortical network. The correlation for all the networks was summed, and then each single network map was divided by the sum of all the maps resulting in normalized maps. Furthermore, a fixed effect analysis across subjects (*n* = 730) was performed using FSL. In the core and matrix comparison, the percent connectivity values of each nucleus in each group (Supplementary Table 4) for left and right thalamus were tested using R software with a two-sample t-test and Wilcoxon rank-sum test, i.e., (i) two-sample *t* test: t.test(core, matrix), (ii) Wilcoxon rank-sum test: Wilcox Test (core, matrix, paired = FALSE, alternative = “two.sided”). Finally, the topic maps were thresholded *p* 0.001 (FSL function ptoz for that (ptoz 0.001 −2, where 0.001 is the *p* value and −2 uses 2-tailed conversion).

### Reporting summary

Further information on research design is available in the Nature Research Reporting Summary linked to this article.

## Supplementary information

Supplementary Information

Description of Additional Supplementary Files

Supplementary Data 1

Supplementary Data 2

Reporting Summary

## Data Availability

The datasets analyzed during the current study are available in the Human connectome project repository (http://www.humanconnectomeproject.org/). The study was performed in agreement with the WU-Minn HCP Consortium Open Access Data Use Terms of the Human connectome project. The study used datasets from the Human connectome project (HCP). We obtained HCP data use permission under open data use terms. Therefore, no further ethical approval was required. The HCP project (https://www.humanconnectomeproject.org/) is an open NIH initiative and has the required ethics approval for data acquisition and public distribution. The source data for the figure plots are available in Supplementary Data 2. In addition, all the source data visualized in the figures are available at: https://figshare.com/articles/journal_contribution/Thalamus_Communications_Biology_paper_data_and_code/21231875

## Code availability

The analysis was performed using the publically available software. The FSL Software library was used for correlation and statistical analysis. https://fsl.fmrib.ox.ac.uk/fsl/fslwiki. Topic-analysis: https://nimare.readthedocs.io/en/latest/index.html. Cortex and thalamus Visualization: https://www.humanconnectome.org/software/connectome-workbench. Visualization codes: Figs. 1, 3, 4a, b, 9 and Supplementary Figs. 1/2/4/5/6 were visualized using the provided data in Microsoft excel. The code for Fig. 1 (using connectome workbench), Fig. 4c (using R-Studio), and Supplementary Fig. 3 (using python-seaborn) are available at: https://figshare.com/articles/journal_contribution/Thalamus_Communications_Biology_paper_data_and_code/21231875.
