# Supplementary material for: Relay and higher-order thalamic nuclei show an intertwined functional association with cortical-networks
Source: Commun Biol. 2022 Nov 4;5:1187. doi: 10.1038/s42003-022-04126-w (PMC9636420; doi:10.1038/s42003-022-04126-w)
Supplement: Supplementary file 1 — Supplementary Information [file 42003_2022_4126_MOESM1_ESM.pdf]

Supplementary Information

JH Atlas			med-vis		occi-vis		lat-vis		DMN		Cb		senmot		aud		exec		R fropar		L fropar	
**lobe**	**sublobe**	**abbr.**	**R#1**	**L#1**	**R#2**	**L#2**	**R3#**	**L3#**	**R#4**	**L#4**	**R#5**	**L#5**	**R#6**	**L#6**	**R#7**	**L#7**	**R8#**	**L8#**	**R#9**	**L#9**	**R#10**	**L#10**
**occipital**	occ	V1	72	76	44	38	15	0	0	0	0	0	0	0	0	0	0	**0**	0	0	**0**	0
		V2	79	74	34	43	0	0	15	13	17	11	0	0	0	0	0	0	0	0	0	0
		V3	43	36	70	86	14	0	0	0	46	24	0	0	0	0	0	0	0	0	0	0
		V4	32	48	83	79	60	48	0	0	66	64	0	0	0	0	0	0	0	0	0	0
		V5	0	0	47	62	87	100	0	0	0	0	0	0	0	0	0	0	0	0	0	0
**parietal**	SPL	5Ci	0	0	**0**	0	0	0	100	100	10	0	80	96	0	0	74	58	0	0	0	0
		5L	0	0	**0**	0	18	22	0	10	0	0	49	74	10	0	23	45	0	0	0	0
		5M	0	0	**0**	11	39	14	84	79	0	0	60	61	0	0	67	62	0	0	0	0
		7A	11	24	14	14	0	12	32	25	0	0	0	11	0	0	0	0	0	0	0	14
		7M	96	75	**0**	0	0	0	100	99	0	0	0	0	0	0	0	0	0	0	0	0
		7P	55	65	16	16	45	41	19	35	0	11	38	39	0	0	0	0	0	0	0	38
	POP	OP1	0	0	**0**	0	0	0	0	0	0	0	55	62	62	83	0	0	75	49	0	0
		OP2	0	0	**0**	0	0	0	0	0	0	0	11	67	28	69	0	0	32	14	0	0
		OP3	0	0	**0**	0	0	0	0	0	0	0	13	81	25	17	0	0	59	38	0	0
		OP4	0	0	**0**	0	0	0	0	0	0	0	0	19	62	79	0	0	43	31	0	0
	IPL	PGp	0	0	22	32	36	34	27	27	0	0	0	0	0	0	0	0	0	0	0	0
		Pga	0	0	**0**	0	47	30	76	68	0	0	0	0	12	0	0	0	0	0	0	35
		PFt	0	0	**0**	0	46	63	0	0	0	14	84	92	0	0	0	0	36	PFt	0	44
		PFop	0	0	**0**	0	0	62	0	0	0	0	71	97	0	20	0	0	61	82	0	0
		PFm	0	0	**0**	0	33	27	67	82	0	0	0	0	12	13	0	0	79	0	0	44
		PFcm	0	0	**0**	0	53	41	0	14	0	0	65	41	37	73	0	0	88	39	0	19
		PF	0	0	**0**	0	55	51	18	31	0	0	22	25	28	23	0	0	64	13	0	42
	IPS	hIP1	0	0	**0**	0	0	0	62	70	21	38	0	11	0	0	0	0	47	34	0	61
		hIP2	0	0	**0**	0	16	19	0	25	0	30	71	45	0	0	0	0	75	20	0	92
		hIP3	0	0	34	45	0	0	13	44	57	52	17	27	0	0	0	0	68	25	0	75
**frontal**	PSMC	BA1	0	0	0	0	15	42	0	0	0	0	45	73	0	0	0	0	0	0	0	18
		BA2	0	0	**0**	0	57	80	0	0	0	27	93	92	0	0	0	0	0	0	0	73
		BA3a	0	12	**0**	17	0	0	23	11	19	54	69	73	36	29	27	13	0	0	0	37
		BA3b	23	28	**0**	0	0	26	0	0	12	46	62	67	27	27	15	18	0	0	0	30
	PMC	BA4a	0	0	24	24	0	0	38	33	0	12	80	89	13	0	48	69	0	0	0	0
		BA4p	15	25	**0**	15	0	0	17	23	23	64	73	76	30	29	20	16	0	0	0	29
		BA6	0	0	13	10	0	0	0	0	0	18	52	57	0	0	26	26	0	0	0	16
	Broc	BA45	0	0	**0**	0	25	0	0	0	0	0	0	13	0	0	38	18	67	18	40	89
		BA44	0	0	**0**	0	41	0	0	0	0	0	0	0	0	0	25	22	75	31	0	84
**temporal**	PAC	TE1.0	0	0	**0**	0	0	0	0	0	0	0	0	22	100	100	0	0	46	47	0	0
		TE1.1	0	0	**0**	0	0	0	0	0	0	0	0	0	100	100	0	0	0	13	0	14
		TE1.2	0	0	**0**	0	0	0	0	0	0	0	0	0	92	94	0	0	0	0	0	0
	Ins	Ig1	0	0	**0**	0	0	0	0	0	0	0	0	0	100	98	0	0	0	0	0	0
		Ig2	0	0	**0**	0	0	0	0	0	0	0	0	25	66	85	0	0	0	20	0	0
		Id1	0	0	**0**	0	0	0	0	0	0	0	0	30	94	58	0	0	0	0	0	0
	Hip	sub	20	0	**0**	0	0	0	0	12	14	0	0	0	0	0	54	20	0	0	0	0
		den	17	0	21	21	0	0	0	0	12	0	0	0	15	0	54	0	0	0	0	0
		amms	0	0	**0**	0	0	0	0	0	0	0	0	0	17	0	0	0	0	0	0	13
		entor	0	0	**0**	0	0	0	0	0	0	0	0	0	0	0	0	0	0	0	0	11
	Amg	Amg	0	0	**0**	0	22	0	0	0	0	0	0	0	0	0	0	0	0	0	0	26

Supplementary Table 1: Percent overlap of cortical areas of RSN (#1-10) with the Jülich histology atlas (JH) derived from 45 areas, which covered $\geq$ 10%. Bold numbers indicate the local maximum within each network.

Abbreviations of cortical areas of the Jülich histology atlas (JHA):

BA: Brodmann area; Broc: Broca's region: BA44: BA45; premotor cortex: BA6; ; Primary motor cortex: PMC: BA4a, BA4p; primary somatosensory cortex: PSM: BA3a, BA3b, BA1, BA2; parietal operculum: POP: OP 1, OP 2, OP 3, OP 4; ; inferior parietal lobule: IPL: PF, PFcm, PFm, PFop, PFt, Pga, PGp; intraparietal sulcus: IPS: hIP1, hIP2, hIP3; ; superior parietal lobule: SPL: 5Ci, 5L, 5M, 7AA, 7M, 7P, 7P; visual cortex: V1, V2, V3v, V4, V5; Primary auditory cortex: PAC: TE 1.0, TE 1.1, TE 1.2; ; Hippocampus formation: hipp: cornu ammonis: CA, entorhinal cortex: EC, Subiculum: sub; ; Amygdala complex: Amg: Centro-median nucleus: CM, Latero-basal nucleus: LB, Superficial nucleus: SF.

Brodman Atlas			med-vis		occi-vis		lat-vis		DMN		Cb		senmot		aud		exec		R fropar		L fropar	
**n**	**lobes**	**area**	**R#1**	**L#1**	**R#2**	**L#2**	**R3#**	**L3#**	**R#4**	**L#4**	**R#5**	**L#5**	**R#6**	**L#6**	**R7#**	**L#7**	**R8#**	**L8#**	**R#9**	**L#9**	**R#10**	**L#10**
**1**	**occi**	**17**	***89***	***83***	39	47	20	5	13	8	16	16	0	0	2	1	12	8	0	0	0	1
**2**		**18**	70	77	50	52	17	10	7	7	35	33	11	2	7	4	5	3	0	0	1	1
**3**		**19**	52	47	***57***	***57***	32	42	3	3	32	31	6	0	0	0	0	1	1	0	0	0
**4**	**par**	**5**	0	0	0	0	29	33	29	21	0	0	58	64	16	9	51	48	0	0	0	0
**5**		**7**	30	25	25	30	10	8	22	28	7	6	3	5	0	0	3	2	17	5	0	31
**6**		**23**	30	19	5	7	0	0	***100***	95	10	6	31	34	0	0	34	22	4	2	0	0
**7**		**29**	1	2	0	0	0	0	93	***98***	0	0	0	0	0	0	0	0	0	0	0	0
**8**		**30**	15	14	0	1	1	0	14	19	***48***	32	31	3	4	4	38	28	3	0	0	18
**9**		**39**	2	0	8	5	29	31	56	67	1	0	0	0	0	0	0	0	6	0	0	8
**10**	**temp**	**20**	0	0	7	5	16	3	8	0	9	1	0	0	16	13	1	0	0	0	2	25
**11**		**21**	0	0	0	0	46	27	22	13	1	0	0	0	72	58	0	0	0	0	1	44
**12**		**22**	0	0	0	0	49	23	24	20	1	0	0	1	*80*	87	0	0	16	0	0	41
**13**		**28**	3	1	0	0	4	0	0	0	0	0	0	0	0	0	0	0	0	0	0	0
**14**		**34**	9	3	0	0	22	0	0	0	0	0	0	0	0	0	0	0	0	0	0	0
**15**		**35**	3	2	0	0	0	0	0	20	4	7	0	0	0	0	40	18	9	0	4	14
**16**		**36**	0	3	0	0	0	0	0	0	0	0	0	0	0	0	0	0	0	0	0	12
**17**		**37**	13	5	32	31	68	54	4	3	37	40	11	3	4	3	0	0	0	1	3	36
**18**		**38**	3	0	0	0	0	0	0	0	0	2	5	8	14	12	28	6	5	0	0	14
**19**		**40**	0	0	1	0	7	17	35	50	13	33	20	21	0	0	0	0	70	24	2	74
**20**		**41**	0	0	0	0	58	40	43	12	0	0	4	9	52	90	0	0	39	11	0	4
**21**		**42**	0	0	0	0	***94***	73	44	24	0	0	16	37	73	***93***	0	0	***97***	21	0	64
**22**	**fron**	**1**	0	0	0	0	17	52	0	0	0	0	50	75	0	0	0	15	0	0	0	15
**23**		**2**	0	0	0	0	64	***92***	6	1	0	7	***84***	***91***	1	0	1	4	41	14	0	49
**24**		**3**	2	5	0	0	24	53	1	1	5	24	81	90	5	4	4	10	1	0	0	47
**25**		**4**	8	10	23	25	0	10	23	16	10	20	74	79	19	15	28	38	0	0	0	15
**26**		**6**	1	3	13	10	7	4	6	7	8	28	54	57	12	7	11	12	10	1	0	22
**27**		**8**	0	0	0	0	0	0	0	0	0	2	0	0	0	0	***91***	***87***	0	0	19	36
**28**		**9**	0	0	0	0	4	0	1	0	0	0	0	0	2	1	53	36	6	1	1	21
**29**		**10**	0	0	0	0	0	0	12	10	0	1	0	0	0	0	0	1	16	6	0	6
**30**		**11**	6	0	0	0	1	0	11	10	7	4	2	0	6	1	0	0	10	1	0	2
**31**		**24**	0	0	0	0	0	0	9	5	36	***57***	35	34	0	0	71	68	58	***42***	1	2
**32**		**25**	25	7	0	0	18	16	7	8	10	10	26	25	17	7	1	0	0	0	8	19
**33**		**32**	0	0	0	0	0	0	4	1	6	10	2	2	0	0	76	67	15	3	4	19
**34**		**43**	40	25	0	8	0	7	0	0	6	18	5	4	22	36	0	0	0	0	0	4
**35**		**44**	0	0	4	10	57	0	0	0	0	7	0	0	2	0	59	40	83	4	19	***96***
**36**		**45**	0	0	0	1	24	0	0	0	0	0	0	16	3	1	37	19	83	25	***43***	95
**37**		**46**	3	1	0	0	0	0	0	0	0	0	0	0	0	0	16	15	76	40	0	31
**38**		**47**	0	0	0	0	0	0	0	0	0	1	8	18	0	1	18	14	66	11	2	68
**39**	**ins**	**13**	4	1	1	1	8	4	3	2	1	3	14	27	25	30	4	1	54	36	4	27
**40**		**14**	0	0	0	0	0	0	0	0	0	0	0	0	0	0	0	0	0	0	0	0

Supplementary Table 2: Percent overlap of cortical areas of RSN (#1-10) with the Brodmann Histology Atlas (BA) derived from 40 areas, which covered ≥ 10%. Bold numbers indicate the local maximum within each network. Note: areas BA 12-16, 31, and 33 are missing!

List of Brodman Areas:

BA-1 Primary somatosensory cortex; BA-2 Primary somatosensory cortex; BA-3 Primary somatosensory cortex; BA-4 Primary motor cortex; BA-5 Cortex postcentral gyrus (Somatosensory Association); BA-6 Premotor cortex and Supplementary Motor Cortex; BA-7 Somatosensory Association Cortex; BA-8 Includes Frontal eye fields; BA-9 Dorsolateral prefrontal cortex; BA-10 Anterior prefrontal cortex (most rostral part of superior and middle frontal gyri; BA-11 Orbitofrontal area (orbital and rectus gyri, plus part of the rostral part of the superior frontal gyrus); BA-12 Orbitofrontal area (part of BA11, area between superior frontal gyrus and inferior rostral sulcus); BA-13 Insular cortex; BA-14 Insular cortex; BA-15 Anterior Temporal Lobe; BA-16 Insular cortex; BA-17 Primary visual cortex (V1); BA-18 Secondary visual cortex (V2; BA-19 Associative visual cortex (V3,V4,V5); BA-20 Inferior temporal gyrus; BA-21 Middle temporal gyrus; BA-22 Superior temporal gyrus; BA-23 Ventral posterior cingulate cortex; BA-24 Ventral anterior cingulate cortex; BA-25 Subgenual area; BA-26 Ectosplenial portion of the retrosplenial region; BA-27 Piriform cortex; BA-28 Ventral entorhinal cortex; BA-29 Retrosplenial cingulate cortex; BA-30 Part of cingulate cortex; BA-31 Dorsal Posterior cingulate cortex; BA-32 Dorsal anterior cingulate cortex; BA-33 Part of anterior cingulate cortex; BA-34 Dorsal entorhinal cortex (parahippocampal gyrus); BA-35 Perirhinal cortex (in the rhinal sulcus); BA-36 Ectorhinal area, now part of the perirhinal cortex (in the rhinal sulcus; BA-37 Fusiform gyrus; BA-38 Temporopolar area (most rostral part of the superior and middle temporal gyri); BA-39 Angular gyrus; BA-40 Supramarginal gyrus; BA-41 Auditory cortex; BA-42 Auditory cortex; BA-43 Primary gustatory cortex; BA-44 Pars opercularis, part of the inferior frontal gyrus and part of Broca's area; BA-45 Pars triangularis, part of the inferior frontal gyrus and part of Broca's area; BA-46 Dorsolateral prefrontal cortex; BA-47 Pars orbitalis, part of the inferior frontal gyrus; BA-48 Retrosubicular area (a small part of the medial surface of the temporal lobe); BA-52 Parainsular area (at the junction of the temporal lobe and the insula).

#	Harvard-Oxford		med-vis		occi-vis		lat-vis		DMN		Cb		senmot		aud		exec		R fropar		L fropar	
#	lobe	area	R#1	L#1	R#2	L#2	R3#	L3#	R#4	L#4	R#5	L#5	R#6	L#6	R7#	L#7	R8#	L8#	R#9	L#9	R#10	L#10
1		LOC s	24	20	28	28	12	15	13	14	0	2	1	0	0	0	0	0	6	1	0	11
2		LOC i	10	7	55	62	48	61	2	1	17	3	1	0	0	0	0	0	0	0	1	6
3		Cal C	***100***	***99***	16	20	29	28	7	1	3	1	0	0	1	0	17	6	0	0	0	0
4	occi	LG	87	89	11	15	29	21	1	2	62	65	32	6	6	4	6	3	0	0	0	0
5		Occ fusi G	57	62	***69***	***80***	34	40	6	11	86	***85***	1	0	6	0	0	0	0	0	7	0
6		supracal	95	89	0	1	1	1	8	9	0	0	0	0	16	11	14	15	0	0	0	0
7		Occr Pole	36	36	54	55	5	1	0	1	1	1	0	0	0	0	3	2	0	0	0	1
8		Postcen G	4	3	1	1	11	28	10	6	13	2	62	69	11	8	13	21	2	2	0	22
9		SPL	0	0	6	8	36	38	10	18	25	13	46	51	5	3	3	1	14	11	2	47
10		SMG a	0	0	0	0	36	54	8	7	9	0	65	56	0	1	0	0	59	25	0	34
11		SMG p	0	0	0	0	39	25	39	59	9	1	14	1	17	20	0	0	62	11	0	40
12	par	Ang G	0	0	0	0	33	25	71	76	2	4	0	0	1	1	0	0	37	4	1	23
13		Cin G p	9	4	3	7	0	0	***81***	***90***	4	10	25	27	0	0	30	20	0	0	0	0
14		PreCun C	45	39	0	0	9	4	80	78	1	1	13	11	1	0	17	14	4	0	0	1
15		Cun C	95	98	0	0	0	0	35	19	0	0	0	0	0	0	0	0	0	0	0	0
16		Par Op C	0	0	0	0	20	24	1	3	0	0	70	67	34	73	0	0	71	54	0	2
17		STG a	0	0	0	0	0	0	0	0	0	0	0	0	83	89	0	0	0	0	0	0
18		STG p	0	0	0	0	29	14	0	0	0	2	0	0	81	83	1	0	4	0	0	39
19		MTG a	0	0	0	0	0	0	0	0	0	0	0	0	31	35	0	0	0	0	0	0
20		MTG p	0	0	0	0	28	4	20	0	0	7	0	0	68	63	0	0	0	0	0	41
21		MTG to	0	0	0	0	***86***	***75***	31	26	0	0	0	0	39	16	0	0	2	0	1	68
22		ITG p	0	0	2	4	12	3	5	0	0	2	0	0	2	9	0	0	0	0	0	24
23	tem	ITG t	1	0	28	43	57	44	0	0	24	6	0	0	3	0	0	0	0	0	1	78
24		Parahip a	4	4	0	0	3	0	0	6	0	0	0	0	0	0	4	1	0	0	0	15
25		Parahip p	6	2	7	4	2	3	0	2	12	18	3	2	5	7	50	18	0	0	0	19
26		TFC p	0	0	16	19	12	5	9	0	18	24	0	0	1	3	0	0	0	0	8	42
27		TOC fusi	8	14	50	53	79	64	0	0	***87***	73	15	0	0	0	0	0	0	0	6	22
28		Planum P	0	0	0	0	0	0	0	0	0	0	0	10	80	76	0	0	14	3	0	0
29		Heschl	0	0	0	0	0	0	0	0	0	0	0	22	***100***	***100***	0	0	48	46	0	0
30		Planum T	0	0	0	0	26	27	0	0	0	0	6	15	96	***100***	0	0	45	12	0	21
31		FP	0	0	0	0	0	0	1	2	0	0	1	1	0	0	6	2	28	10	2	17
32		SFG	0	0	1	0	0	0	0	0	7	3	22	22	0	0	49	48	0	0	6	24
33		MFG	2	0	0	0	16	1	0	0	7	1	2	4	5	2	***65***	***60***	39	10	8	50
34		iFG trian	1	0	0	1	38	0	0	0	0	0	0	25	5	2	26	2	62	20	***48***	***97***
35		iFG oper	0	0	5	2	52	0	0	0	1	0	0	0	0	0	16	0	86	22	28	93
36		Precen G	4	7	21	24	6	3	18	19	26	9	52	57	17	14	14	15	10	2	0	23
37	fron	SMA	10	3	11	7	0	0	13	8	31	8	***83***	***87***	4	0	1	0	34	9	0	0
38		Subcal C	16	1	0	0	11	11	1	4	7	13	26	17	36	13	0	0	0	0	9	12
39		Paracin C	0	0	0	0	0	0	14	10	1	5	2	2	0	0	62	54	17	4	5	14
40		Cin G a	4	5	1	1	2	0	24	21	34	18	29	30	0	0	46	42	27	15	0	0
41		FOC	19	2	0	0	5	0	0	0	2	0	13	17	1	0	26	15	16	1	1	31
42		Fron Op C	0	0	0	0	0	0	0	0	0	0	0	1	2	3	4	0	***100***	***74***	1	92
43		Cen Op C	0	1	0	0	0	0	0	0	0	0	14	59	56	50	0	0	74	64	0	14
44		FMC	0	0	0	0	0	0	36	29	1	8	0	0	1	0	0	0	0	0	0	0
45	Ins	IC	0	0	0	0	0	0	0	0	5	2	2	30	18	18	5	2	58	41	0	14

Supplementary Table 3: Percent overlap of cortical areas of RSN (#1-10) with the Harvard-Oxford Atlas (HO) derived from 45 areas, which covered $\geq$ 10%. Bold numbers indicate the local maximum within each network.

Abbreviations for Harvard Oxford Cortical Atlas Names:

Ang G: Angular Gyrus; Cen Op C: Central Opercular Cortex; Cin G ant: Cingulate Gyrus, anterior division; Cin G post: Cingulate Gyrus, posterior division; Cun C: Cuneal Cortex; FMC: Frontal Medial Cortex; FOC: Frontal Orbital Cortex; FP: Frontal Pole; Fron Op C: Frontal Operculum Cortex; Heschl: Heschl's Gyrus (includes H1 and H2); IC: Insular Cortex; iFG oper: Inferior Frontal Gyrus, pars opercularis; iFG trian: Inferior Frontal Gyrus, pars triangularis; Intracal C: Intracalcarine Cortex; ITG ant: Inferior Temporal Gyrus, anterior division; ITG post: Inferior Temporal Gyrus, posterior division; ITG tem: Inferior Temporal Gyrus, temporooccipital part; LG: Lingual Gyrus; LOC inf: Lateral Occipital Cortex, inferior division; LOC sup: Lateral Occipital Cortex, superior division; MFG: Middle Frontal Gyrus; MTG ant: Middle Temporal Gyrus, anterior division; MTG post: Middle Temporal Gyrus, posterior division; MTG temp: Middle Temporal Gyrus, temporooccipital part; Occ Fusi G: Occipital Fusiform Gyrus; Occr Pole: Occipital Pole; Par Op C: Parietal Operculum Cortex; Paracin C: Paracingulate Gyrus; Parahip G ant: Parahippocampal Gyrus, anterior division; Parahip G post: Parahippocampal Gyrus, posterior division; Planum P: Planum Polare; Planum T: Planum Temporale; Postcen G: Postcentral Gyrus; Precen G: Precentral Gyrus; PreCun C: Precuneous Cortex; SFG: Superior Frontal Gyrus; SMA: Supplementary Motor Cortex ; SMG ant: Supramarginal Gyrus, anterior division; SMG post: Supramarginal Gyrus, posterior division; SPL: Superior Parietal Lobule; STG ant: Superior Temporal Gyrus, anterior division; STG post: Superior Temporal Gyrus, posterior division; Subcal C: Subcallosal Cortex; supracal C: Supracalcarine Cortex; Tem P: Temporal Pole; TFC ant: Temporal Fusiform Cortex, anterior division; TFC post: Temporal Fusiform Cortex, posterior division; TOC fusi: Temporal Occipital Fusiform Cortex.

Cortical Resting State Networks	Behavioral Domains (Strongest in the heatmap; Figure 2 Smith et al., 2009)
Medial visual (MV)	cognition-reasoning, cognition-space, perception-vision, perception-motion, and perception-vision-shape
Occipital visual (OV)	cognition-language-orthography, cognition-space, perception-vision-shape
Lateral visual (LV)	perception-vision-shape, perception-vision-motion, perception-vision, cognition-space, cognition-language-orthography
Default mode (DMN)	negative contrasts of cognition-memory-explicit, cognition-reasoning
Cerebellum (CB)	action-execution, action-execution-speech, perception-somesthesis, perception-somesthesis-pain
Sensorimotor (SM)	action-execution, perception-somesthesis, perception-somesthesis-pain
Auditory (AU)	action-execution-speech, perception-audition, cognition-language-phonology, cognition-language
Executive control (EX)	action-inhibition, cognition, cognition-attention, cognition-memory-explicit, cognition-memory-working, cognition-reasoning, emotion, perception-somesthesis-pain
Right Frontoparietal (RF)	action-inhibition, cognition-attention, cognition-memory-working, cognition-reasoning, perception-somesthesis, perception-somesthesis-pain
Left Frontoparietal (LF)	cognition-language-phonology, cognition-language-semantics, cognition-memory-explicit, cognition-memory-working

Supplementary Table 4: Behavioral Domains associated with functional cortical resting state networks: Enlisted are all behavioral domains (https://brainmap.org/taxonomy/behaviors/) obtained from the brain map database [1], which show a correspondence with the resting state networks [2].

29 Thalamic Nuclei					
**Anterior group**	**Abbr.**	**Voxel**	**Pulvinar**	**Abbr.**	**Voxel**
Anterior dorsal nucleus	AD	21	Anterior pulvinar	PuA	376
Anterior medial nucleus	AM	33	Inferior pulvinar	PuI	35
Anterior ventral nucleus	AV	94	Lateral pulvinar	PuL	110
Lateral dorsal nucleus	LD	64	Medial pulvinar	PuM	74
**Medial group**			**Lateral group**		
Mediodorsal nucleus	MD	246	Ventral posterior lateral nucleus	VPL	182
Magnocellular part	MDmc		Anterior part	VPLa	
Parvocellular part	MDpc		Posterior part	VPLp	
**Intralaminar Group**			Ventral posterior medial nucleus	VPM	48
Central lateral nucleus	CL	291	Ventral posterior inferior nucleus	VPI	48
Centre médian nucleus	CM	85	Ventral lateral nucleus	VL	368
Parafascicular nucleus	Pf	85	Ventral lateral anterior nucleus	VLa	
Subparafascicular nucleus	sPf	19	Ventral lateral posterior nucleus	VLp	
Central medial nucleus	CeM	82	Dorsal part	VLpd	
**Midline Group**			Ventral part	VLpv	
Medioventral nucleus	MV	24	Ventral anterior nucleus	VA	194
Paraventricular nucleus	Pv	14	Magnocellular part	VAmc	
**Hb/Li/SG**			Parvocellular part	VApc	
Habenular nucleus	Hb	26	Ventral medial nucleus	VM	72
Limitans nucleus	Li	72			
Suprageniculate nucleus	SG	40			
**Posterior Group:**					
Lateral posterior nucleus	LP	72	**Total number of Voxel**	**Sum**	**2961**
Posterior nucleus	Po	40		**AVG**	**102**
Medial geniculate nucleus	MGN	74		MAX	376
Lateral geniculate nucleus	LGN	72		MIN	14

Supplementary Table 5: List of 29 thalamic nuclei ordered in eight nuclei groups with their full name and abbreviations according to Morel [3].

		med vis		occ vis		lat vis		DMN		CB		sen-mot		aud		exec		R fro-par		L fro-par	
**group**	**nucleus**	**R#1**	**L#1**	**R#2**	**L#2**	**R3#**	**L3#**	**R#4**	**L#4**	**R#5**	**L#5**	**R#6**	**L#6**	**R7#**	**L#7**	**R exe**	**L exe**	**R#9**	**L#9**	**R#10**	**L#10**
**Anterior**	**AD**	9,98	9,89	10,08	9,83	9,93	9,88	***10,64***	***10,79***	9,63	9,92	9,98	***9,72***	10,21	9,93	10,35	10,07	10,12	10,30	***9,07***	***9,66***
	**AM**	10,00	9,84	9,99	9,84	9,95	9,83	10,15	10,33	9,96	10,16	10,14	9,87	10,34	9,96	10,22	10,05	10,01	10,30	9,24	9,81
	**AV**	9,88	9,80	10,09	9,89	9,97	9,87	10,29	10,39	9,36	9,77	10,17	9,88	10,39	10,05	10,37	10,14	10,24	10,47	9,23	9,74
	**LD**	***9,73***	9,74	9,99	9,84	9,97	9,73	10,63	10,54	***8,95***	9,67	10,26	9,89	10,53	10,15	10,22	10,07	***10,49***	10,59	9,23	9,79
**MD**	**MD**	9,85	9,74	9,99	9,83	9,99	9,76	10,05	10,22	9,53	9,86	10,32	10,03	10,32	10,02	10,10	10,07	10,38	10,59	9,33	9,88
**intralaminar**	**CM**	9,88	9,72	9,87	9,83	9,80	9,76	9,65	***9,82***	9,74	9,75	10,72	10,68	10,39	10,20	10,30	10,00	10,23	10,42	9,46	9,93
	**Pf**	9,97	9,79	9,82	9,78	9,74	9,64	9,62	9,82	10,26	10,20	10,30	10,29	10,27	10,10	10,27	9,89	10,08	10,28	9,43	9,97
	**sPf**	9,95	9,81	9,89	9,84	9,85	9,75	9,63	9,89	10,02	9,75	10,39	10,31	10,44	10,30	11,27	***10,31***	10,16	10,31	9,48	10,00
	**CL**	9,82	9,76	10,00	9,84	9,98	9,76	10,10	10,24	9,33	9,89	10,38	10,08	10,37	10,06	10,24	10,06	10,39	10,55	9,33	9,82
**midline**	**CeM**	9,99	9,82	9,97	9,88	9,94	9,79	9,92	10,18	9,97	9,87	10,25	9,98	10,39	10,13	10,08	10,05	10,09	10,34	9,38	9,96
	**Pv**	10,04	9,78	9,99	9,91	9,97	9,79	10,13	10,52	10,05	10,05	10,23	9,93	10,33	9,99	10,50	10,13	***9,92***	10,16	9,35	9,96
	**MV**	10,03	9,81	9,94	9,89	9,92	9,79	9,83	10,16	10,16	9,79	10,25	10,04	10,37	10,16	9,99	9,90	10,02	10,28	9,40	***10,02***
**Hb**	**Hb**	9,97	9,83	***9,59***	***9,58***	9,67	9,46	***9,61***	9,89	***10,63***	***11,07***	***9,90***	9,87	***9,93***	***9,77***	10,19	10,05	10,14	10,33	9,28	9,89
**Li/SG**	**Li**	9,86	9,84	9,66	9,73	***9,62***	9,57	9,68	9,88	10,04	10,45	10,11	10,34	10,20	10,04	9,97	9,85	10,11	10,21	9,43	9,89
	**SG**	9,82	9,89	9,75	9,81	9,65	9,66	9,75	9,93	9,54	10,04	10,48	10,54	10,51	10,23	10,49	9,88	10,10	10,11	9,47	9,86
**posterior G**	**LP**	9,82	***9,71***	9,98	9,93	9,87	9,72	10,28	10,24	9,14	9,28	10,82	10,45	10,52	10,31	9,85	9,86	10,34	10,56	9,39	9,93
	**Po**	9,87	9,87	9,87	9,83	9,75	9,75	9,77	9,93	9,53	9,85	10,78	10,74	10,58	10,24	10,33	9,84	10,03	10,10	9,47	9,85
	**MGN**	9,91	9,91	9,95	10,04	9,73	9,77	9,74	10,00	9,35	9,60	10,68	10,51	10,66	10,34	***11,29***	10,05	10,07	10,06	9,42	9,89
	**LGN**	***10,05***	***10,18***	10,33	10,02	9,89	***9,90***	9,78	10,07	9,34	9,39	10,43	10,14	10,65	***10,43***	10,93	9,91	10,03	10,07	***9,53***	9,94
**Pulvinar**	**PuA**	9,83	9,72	9,92	9,87	9,78	9,76	9,84	9,91	9,32	9,51	11,15	***11,06***	10,49	10,27	10,06	***9,75***	10,16	10,28	9,44	9,89
	**PuI**	10,00	9,99	***10,43***	***10,43***	9,81	9,87	9,89	10,20	9,33	9,39	10,34	10,05	10,55	10,31	10,15	9,79	10,01	***10,01***	9,50	9,95
	**PuL**	9,94	9,89	10,15	10,03	9,83	9,85	10,16	10,33	9,29	9,37	10,71	10,37	10,56	10,34	9,83	9,78	10,03	10,10	9,50	9,94
	**PuM**	9,88	9,88	9,94	9,90	9,77	9,72	10,22	10,44	9,29	9,78	10,47	10,21	10,44	10,20	10,41	9,82	10,14	10,17	9,44	9,89
**Lateral**	**VPL**	9,84	9,73	10,01	9,97	9,95	9,84	9,80	9,92	9,16	***9,24***	11,06	10,69	10,65	10,30	9,90	9,78	10,31	10,50	9,40	9,94
	**VPM**	9,87	9,76	9,92	9,88	9,87	9,84	9,64	9,85	9,52	9,52	10,95	10,71	10,67	10,31	***9,81***	9,88	10,19	10,37	9,44	9,91
	**VPI**	9,89	9,85	9,87	9,84	9,77	9,82	9,70	9,95	9,42	9,41	***11,27***	10,98	***10,78***	10,36	9,94	9,85	9,97	10,08	9,43	9,91
	**VL**	9,82	9,72	10,11	9,99	***10,10***	9,86	9,85	10,00	9,12	9,26	10,55	10,23	10,63	10,27	10,14	10,10	10,46	***10,72***	9,37	9,92
	**VA**	9,88	9,77	10,08	9,94	10,06	9,84	9,93	10,05	9,34	9,43	10,28	10,04	10,56	10,26	10,00	10,03	10,36	10,67	9,37	9,89
	**VM**	9,89	9,80	10,00	9,94	10,07	9,88	9,74	9,99	9,37	9,38	10,52	10,20	10,70	10,38	9,94	9,93	10,31	10,51	9,46	10,00

Supplementary Table 6: Percent-connectivity of RSN to 29 thalamic nuclei. Bold numbers indicate the local maximum/minimum within each network.

AD	AM	AV	LD	CM	Pf	sPf	CL	CeM	MD	Pv	MV	Hb	Li	SG	LP	Po	MGN	LGN	PuA	Pul	PuL	PuM	VPL	VPM	VPI	VL	VA	VM
anterior				intralaminar					midline			Hb	Li/SG		post G		MGN	LGN	pulvinar				lateral					
Anterior				medial									posterior										Lateral					

Right	anterior	Left	anterior
DMN #4	10,43	DMN #4	10,51
AU #7	10,37	RF #9	10,42
EX #8	10,29	EX #8	10,08
RF #9	10,22	AU #7	10,02
SM #6	10,14	CB #5	9,88
OV #2	10,04	OV #2	9,85
LV #3	9,96	SM #6	9,84
MV #1	9,9	LV #3	9,83
CB #5	9,47	MV #1	9,82
LF #10	9,19	LF #10	9,75

Right	intralam	Left	intralam
EX #8	10,52	RF #9	10,39
SM #6	10,45	SM #6	10,34
AU #7	10,37	AU #7	10,16
RF #9	10,22	EX #8	10,07
MV #1	9,9	DMN #4	9,94
OV #2	9,89	LF #10	9,93
LV #3	9,84	CB #5	9,9
CB #5	9,84	OV #2	9,82
DMN #4	9,75	MV #1	9,77
LF #10	9,43	LV #3	9,73

Right	midline	Left	midline
AU #7	10,36	RF #9	10,34
SM #6	10,26	DMN #4	10,27
EX #8	10,17	AU #7	10,07
RF #9	10,1	EX #8	10,03
DMN #4	9,98	SM #6	10
MV #1	9,98	LF #10	9,95
OV #2	9,97	CB #5	9,89
LV #3	9,96	OV #2	9,88
CB #5	9,93	MV #1	9,79
LF #10	9,36	LV #3	9,79

Right	Hb	Left	Hb
CB #5	*10,63*	CB #5	*11,07*
EX #8	10,19	RF #9	10,33
RF #9	10,14	EX #8	10,05
MV #1	9,97	DMN #4	9,89
AU #7	*9,93*	LF #10	9,89
SM #6	*9,9*	SM #6	9,87
LV #3	9,67	MV #1	9,83
DMN #4	*9,61*	AU #7	*9,77*
OV #2	*9,59*	OV #2	*9,58*
LF #10	9,28	LV #3	9,46

Right	Li/SG	Left	Li/SG
AU #7	10,36	SM #6	10,44
SM #6	10,29	CB #5	10,25
EX #8	10,23	RF #9	10,16
RF #9	10,1	AU #7	10,13
MV #1	9,84	DMN #4	9,91
CB #5	9,79	LF #10	9,87
DMN #4	9,72	MV #1	9,87
OV #2	9,71	EX #8	9,87
LV #3	9,64	OV #2	9,77
LF #10	9,45	LV #3	9,62

Right	post G	Left	post G
SM #6	10,8	SM #6	10,6
AU #7	10,55	RF #9	10,33
RF #9	10,19	AU #7	10,28
EX #8	10,09	DMN #4	10,09
DMN #4	10,03	LF #10	9,89
OV #2	9,92	OV #2	9,88
MV #1	9,84	EX #8	9,85
LV #3	9,81	MV #1	9,79
LF #10	9,43	LV #3	9,73
CB #5	9,33	CB #5	9,57

Right	MGN	Left	MGN
EX #8	11,29	SM #6	10,51
SM #6	10,68	AU #7	10,34
AU #7	10,66	RF #9	10,06
RF #9	10,07	EX #8	10,05
OV #2	9,95	OV #2	10,04
MV #1	9,91	DMN #4	10
DMN #4	9,74	MV #1	9,91
LV #3	9,73	LF #10	9,89
LF #10	9,42	LV #3	9,77
CB #5	9,35	CB #5	9,6

Right	LGN	Left	LGN
EX #8	10,93	AU #7	10,43
AU #7	10,65	MV #1	10,18
SM #6	10,43	SM #6	10,14
OV #2	10,33	DMN #4	10,07
MV #1	10,05	RF #9	10,07
RF #9	10,03	OV #2	10,02
LV #3	9,89	LF #10	9,94
DMN #4	9,78	EX #8	9,91
LF #10	9,53	LV #3	9,9
CB #5	9,34	CB #5	9,39

Right	pulvinar	Left	pulvinar
SM #6	10,67	SM #6	10,42
AU #7	10,51	AU #7	10,28
EX #8	10,11	DMN #4	10,22
OV #2	10,11	RF #9	10,14
RF #9	10,09	OV #2	10,06
DMN #4	10,03	LF #10	9,92
MV #1	9,91	MV #1	9,87
LV #3	9,8	LV #3	9,8
LF #10	9,47	EX #8	9,78
CB #5	9,31	CB #5	9,51

Right	lateral	Left	lateral
SM #6	10,77	SM #6	10,48
AU #7	10,66	RF #9	10,48
RF #9	10,27	AU #7	10,31
OV #2	10	DMN #4	9,96
LV #3	9,97	EX #8	9,93
EX #8	9,95	LF #10	9,93
MV #1	9,86	OV #2	9,93
DMN #4	9,78	LV #3	9,85
LF #10	9,41	MV #1	9,77
CB #5	9,32	CB #5	9,37

Supplementary Table 7: Nuclei and sub-group of nuclei specific sorted for average percent-connectivity (sum of all nuclei % connectivity/number of nuclei). Darker colors indicate higher and white colors with lower values. The nuclei are organized into seven sub-groups, Hb, LGN, and MGN as single nuclei.

Thalamus Left	Network	Correlation Fixed-effect Sum	Total	**Connectivity in %** Fixed-effect-Sum/Total*100
	**med-vis**	126.31	1288.09	9.81
	**occ-vis**	127.75	1288.09	9.92
	**lat-vis**	125.79	1288.09	9.77
	**DMN**	131.46	1288.09	10.21
	**Cb**	125.11	1288.09	9.71
	**sen-mot**	130.48	1288.09	10.13
	**aud**	131.12	1288.09	10.18
	**exe**	128.72	1288.09	9.99
	**fro-pa-R**	133.95	1288.09	10.40
	**fro-pa-L**	127.41	1288.09	9.89
	**Total**	1288.09		
Thalamus Right	**med-vis**	123.22	1248.00	9.87
	**occ-vis**	125.01	1248.00	10.02
	**lat-vis**	123.96	1248.00	9.93
	**DMN**	124.52	1248.00	9.98
	**Cb**	116.79	1248.00	9.36
	**sen-mot**	130.27	1248.00	10.44
	**aud**	131.36	1248.00	10.53
	**exe**	127.35	1248.00	10.20
	**fro-pa-R**	128.29	1248.00	10.28
	**fro-pa-L**	117.24	1248.00	9.39
	**Total**	1248.00		

Supplementary Table 8: Whole thalamus percent contribution: The sum and percent of normalized group fixed-effect correlation maps from the left and right thalamus. The computed percent connectivity values are displayed.

WTA		med-vis		occi-vis		lat-vis		DMN		SM		aud		exec		R fron-par		L fron-par	
Dice overlap		R #1	L #1	R # 2	L #2	R #3	L #3	R #4	L #4	R #6	L #6	R #7	L #7	R #8	L #8	R #9	L # 9	R #10	L # 10
Anterior	AD	0,00	0,00	0,00	0,00	0,00	0,00	0,10	0,22	0,00	0,00	0,00	0,00	0,04	0,00	0,00	0,00	0,00	0,00
	AM	0,00	0,00	0,00	0,00	0,00	0,00	0,15	0,22	0,00	0,00	0,00	0,00	0,17	0,06	0,00	0,10	0,00	0,03
	AV	0,00	0,00	0,00	0,01	0,00	0,00	0,27	0,32	0,00	0,00	0,00	0,00	0,23	0,08	0,01	0,16	0,00	0,05
	LD	0,00	0,00	0,00	0,00	0,00	0,00	0,27	0,25	0,00	0,01	0,00	0,00	0,06	0,02	0,04	0,13	0,01	0,04
MD	MD	0,00	0,00	0,02	0,00	0,00	0,00	0,29	0,36	0,06	0,09	0,00	0,00	0,24	0,15	0,20	0,31	0,06	0,11
intralaminar	CM	0,00	0,00	0,00	0,00	0,00	0,00	0,02	0,01	0,35	0,40	0,01	0,00	0,14	0,06	0,15	0,17	0,03	0,05
	Pf	0,00	0,00	0,00	0,00	0,00	0,00	0,06	0,05	0,15	0,20	0,01	0,00	0,16	0,23	0,12	0,14	0,03	0,04
	sPf	0,00	0,00	0,00	0,00	0,00	0,00	0,01	0,00	0,12	0,18	0,00	0,00	0,08	0,17	0,11	0,10	0,02	0,03
	CL	0,00	0,00	0,01	0,00	0,00	0,00	0,29	0,36	0,11	0,18	0,00	0,00	0,27	0,06	0,21	0,34	0,07	0,13
midline	CeM	0,00	0,00	0,02	0,00	0,00	0,00	0,22	0,24	0,01	0,02	0,00	0,00	0,24	***0,24***	0,11	0,25	0,03	0,08
	Pv	0,00	0,00	0,00	0,00	0,00	0,00	0,21	0,22	0,00	0,00	0,00	0,00	0,02	0,04	0,00	0,00	0,00	0,00
	MV	0,00	0,00	0,00	0,00	0,00	0,00	0,13	0,15	0,00	0,00	0,00	0,00	0,16	0,20	0,02	0,12	0,00	0,03
Hb	Hb	0,00	0,00	0,00	0,00	0,00	0,00	0,02	0,08	0,00	0,03	0,00	0,00	0,09	0,19	0,02	0,05	0,00	0,01
Li/SG	Li	0,00	0,00	0,00	0,00	0,00	0,05	0,01	0,07	0,10	0,26	0,00	0,16	0,23	0,10	0,02	0,04	0,00	0,01
	SG	0,00	0,00	0,00	0,04	0,03	0,09	0,00	0,03	0,21	0,34	0,07	***0,18***	0,19	0,01	0,00	0,00	0,00	0,00
posterior G	LP	0,00	0,00	0,02	0,00	0,00	0,00	***0,32***	0,16	0,34	0,25	0,00	0,00	0,00	0,00	0,12	0,22	0,03	0,07
	Po	0,00	0,00	0,03	0,04	***0,05***	***0,13***	0,02	0,01	0,41	0,47	0,08	0,11	0,14	0,00	0,00	0,00	0,00	0,00
	MGN	0,01	0,04	0,05	0,15	0,01	0,02	0,00	0,00	0,32	0,34	0,06	0,09	0,13	0,02	0,00	0,00	0,00	0,00
	LGN	0,04	0,06	0,14	0,01	0,00	0,00	0,00	0,00	0,12	0,06	0,01	0,03	0,00	0,00	0,00	0,00	0,00	0,00
Pulvinar	PuA	0,00	0,00	0,00	0,00	0,00	0,00	0,06	0,03	0,48	***0,61***	0,00	0,00	0,08	0,00	0,04	0,05	0,01	0,01
	PuI	0,05	***0,09***	***0,40***	***0,35***	0,03	0,03	0,04	0,03	0,13	0,09	0,02	0,03	0,06	0,00	0,00	0,00	0,00	0,00
	PuL	***0,07***	0,08	0,22	0,13	0,02	0,04	0,20	0,13	0,29	0,22	0,00	0,01	0,00	0,00	0,00	0,01	0,00	0,00
	PuM	0,02	0,03	0,08	0,11	0,01	0,04	0,29	***0,38***	0,24	0,31	0,01	0,04	0,22	0,01	0,01	0,05	0,00	0,02
Lateral	VPL	0,00	0,01	0,02	0,00	0,00	0,00	0,09	0,03	***0,67***	0,56	0,10	0,01	0,01	0,01	0,20	0,27	0,05	0,09
	VPM	0,00	0,00	0,00	0,00	0,00	0,00	0,00	0,00	0,44	0,56	***0,19***	0,01	0,07	0,03	0,18	0,19	0,05	0,06
	VPI	0,00	0,00	0,00	0,00	0,00	0,00	0,00	0,00	0,47	0,47	0,12	0,04	0,02	0,00	0,00	0,00	0,00	0,00
	VL	0,00	0,00	0,03	0,01	0,00	0,00	0,18	0,13	0,19	0,19	0,10	0,01	0,22	0,05	***0,49***	***0,59***	0,12	0,19
	VA	0,00	0,00	0,02	0,01	0,00	0,00	0,20	0,11	0,00	0,00	0,00	0,00	***0,41***	0,08	0,42	0,53	0,17	0,25
	VM	0,00	0,00	0,02	0,00	0,00	0,00	0,03	0,01	0,13	0,20	0,16	0,01	0,17	0,06	0,35	0,38	0,09	0,12

Supplementary Table 9: WTA Results: Dice overlap from 9 cortical RSN to 29 thalamic nuclei. Bold numbers indicate the local maximum within each network.

Names of the list topic maps of Fig. 8

1 network_state_resting;
2 anxiety_trait_personality;
3 cerebellar_cerebellum_basal;
4 cortex_anterior_cingulate;
5 stimulus_time_repetition;
6 gyrus_frontal_inferior;
7 auditory_speech_temporal;
8 reward_feedback_striatum;
9 mpfc_social_medial;
10 memory_working_wm;
11 food_taste_weight;
12 learning_training_practice;
13 women_men_sex;
14 fear_threat_smokers;
15 disease_ad_pd;
16 task_performance_cognitive;
17 response_inhibition_control;
18 motor_cortex_hand;
19 number_ips_numerical;
20 action_actions_observation;
21 control_conflict_task;
22 matter_volume_structural;
23 method_group_approach;
24 asd_autism_group;
25 age_adults_older;
26 spatial_body_human;
27 emotional_amygdala_negative;
28 disorder_adhd_group;
29 social_empathy_moral;
30 stress_ptsd_trauma;
31 decision_making_risk;
32 model_models_prediction;
33 pain_somatosensory_stimulation;
34 memory_retrieval_encoding;
35 frequency_hz_ms;
36 schizophrenia_risk_genetic;
37 placebo_treatment_dopamine;
38 language_reading_word;
39 semantic_category_representations;
40 stimulation_tms_bpd;
41 face_faces_facial;
42 imagery_mental_events;
43 visual_cortex_sensory;
44 magnetic_mechanisms_human;
45 eye_sleep_gaze;
46 motion_perception_visual;
47 hemisphere_language_stroke;
48 attention_attentional_target;
49 prefrontal_cortex_pfc;
50 depression_mdd_state

Supplementary Table 10: ranked neurosynth topic map names with their numbered IDs.

Right Thalamus nuclei percent overlap

Group	Nuclei	MV #1<br>Topic maps 43, 45, 48,42	OV #2<br>Topic maps 43, 46, 41	LV #3<br>Topic maps 46, 20, 43, 41, 39	DMN #4<br>Topic maps 1, 9, 47, 34	CB #5<br>Topic maps 3, 18	SM #6<br>Topic maps 18, 40, 12	AU #7<br>Topic maps 7, 38, 49, 47	EX #8<br>Topic maps 49, 21, 31, 17	Rfro #9<br>Topic maps 17, 21, 10, 33	Lfro #10<br>Topic maps 38, 35, 6, 23
Anterior	AD	1.54	0.00	0.00	0.00	47.69	0.00	0.00	1.54	46.15	0.00
	AM	0.00	0.00	0.00	0.00	76.47	6.86	0.00	2.94	70.59	32.35
	AV	3.67	0.00	0.00	0.00	65.14	5.96	0.00	5.96	51.83	11.01
	LD	11.05	0.00	0.00	0.00	48.07	14.36	0.00	0.00	43.09	0.00
MD	MD	2.80	0.00	0.00	0.00	88.16	28.35	0.00	4.05	88.16	1.56
intralaminar	CM	0.66	0.00	1.99	0.00	99.34	70.20	0.00	3.31	99.34	0.00
	Pf	0.00	0.00	0.00	0.00	85.91	38.26	0.00	6.04	85.91	0.00
	sPf	0.00	0.00	0.00	0.00	90.14	46.48	0.00	12.68	90.14	0.00
	CL	7.04	0.00	0.00	0.00	81.31	33.98	0.00	3.88	79.85	1.21
	CeM	0.00	0.00	0.00	0.00	89.23	12.82	0.00	7.18	89.23	13.85
midline	Pv	0.00	0.00	0.00	0.00	93.33	0.00	0.00	1.67	93.33	13.33
	MV	0.00	0.00	0.00	0.00	95.00	0.00	0.00	2.50	95.00	18.75
Hb	Hb	0.00	0.00	0.00	0.00	45.12	3.66	0.00	0.00	45.12	0.00
Li/SG	Li	0.00	2.92	2.92	9.49	69.34	24.82	0.00	0.00	63.50	0.00
	SG	4.58	8.40	19.85	25.19	72.52	33.59	0.00	0.00	55.73	0.00
posterior G	LP	13.40	0.00	7.18	9.57	88.04	63.64	0.00	0.00	76.08	0.00
	Po	8.45	4.23	33.80	27.46	90.14	58.45	0.00	0.00	76.06	0.00
	MGN	19.48	8.44	39.61	33.12	63.64	38.31	0.00	0.00	46.10	0.00
	LGN	17.36	0.00	20.14	19.44	19.44	14.58	0.00	0.00	11.11	0.00
Pulvinar	PuA	8.87	0.00	18.55	4.03	100.00	84.68	0.00	0.00	100.00	0.00
	PuI	52.76	6.30	33.86	60.63	33.86	16.54	0.00	0.00	12.60	0.00
	PuL	29.50	0.50	26.50	50.50	56.00	36.50	0.00	0.00	29.50	0.00
	PuM	14.04	3.00	11.99	29.59	57.12	24.91	0.00	0.00	35.77	0.00
Lateral	VPL	22.42	0.00	24.20	8.19	95.37	87.54	0.00	0.00	92.17	0.00
	VPM	8.50	0.00	8.00	1.00	95.00	79.50	0.00	5.50	94.50	0.00
	VPI	14.29	0.00	47.62	10.71	96.43	94.05	0.00	1.19	92.86	0.00
	VL	19.92	0.00	2.71	0.00	90.91	64.41	0.00	4.06	86.65	0.39
	VA	8.07	0.00	0.00	0.00	97.52	39.13	0.00	7.45	88.82	13.35
	VM	5.35	0.00	0.53	0.00	99.47	64.17	0.00	10.70	99.47	0.00

Left Thalamus nuclei percent overlap

Group	Nuclei	MV #1	OV #2	LV #3	DMN #4	CB #5	SM #6	AU #7	EX #8	Rfro #9	Lfro #10
Anterior	AD	0.00	0.00	0.00	0.00	70.40	1.23	0.00	0.00	61.70	0.00
	AM	0.00	0.00	0.00	0.00	80.20	1.65	0.00	0.00	60.30	19.80
	AV	3.66	0.00	0.00	0.41	72.80	12.60	0.00	1.22	54.90	5.69
	LD	4.81	0.00	0.00	0.00	67.40	39.60	0.54	0.54	61.00	0.00
MD	MD	1.14	0.00	0.00	0.00	96.00	43.90	0.00	0.29	94.90	0.86
intralaminar	CM	0.00	0.00	0.00	0.00	100.00	95.30	4.03	0.00	100.00	0.00
	Pf	0.00	0.00	0.00	0.00	99.30	64.60	1.39	0.69	99.30	0.00
	sPf	0.00	0.00	0.00	0.00	94.90	75.60	1.28	1.28	94.90	0.00
	CL	2.36	0.00	0.00	0.00	91.70	56.70	0.00	0.00	90.10	0.47
	CeM	0.00	0.00	0.00	0.00	95.30	21.90	0.00	0.93	94.00	8.84
midline	Pv	0.00	0.00	0.00	0.00	93.70	0.00	0.00	0.00	93.70	11.10
	MV	0.00	0.00	0.00	0.00	96.60	5.75	0.00	0.00	96.60	12.60
Hb	Hb	0.00	0.00	0.00	0.00	75.30	30.90	0.00	1.03	75.30	0.00
Li/SG	Li	0.00	0.00	0.00	17.60	94.40	57.70	4.93	0.70	92.30	0.00
	SG	0.79	0.00	0.00	34.60	86.60	56.70	10.20	0.00	78.00	0.00
posterior G	LP	2.34	0.00	0.00	0.00	82.70	80.40	0.47	0.47	79.40	0.00
	Po	0.77	0.00	0.00	29.20	96.20	77.70	10.00	0.00	88.50	0.00
	MGN	6.67	0.67	0.67	34.70	62.00	43.30	6.67	0.00	47.30	0.00
	LGN	2.94	0.74	0.74	8.82	11.80	7.35	0.00	0.00	5.15	0.00
Pulvinar	PuA	0.00	0.00	0.00	2.26	100.00	99.20	7.52	0.00	100.00	0.00
	PuI	31.20	0.80	0.80	46.40	31.20	12.00	0.00	0.00	11.20	0.00
	PuL	12.40	0.50	0.50	29.20	51.00	33.70	0.00	0.00	29.20	0.00
	PuM	6.86	0.19	0.19	28.60	72.80	42.10	0.38	0.00	54.90	0.00
Lateral	VPL	3.68	0.00	0.00	3.68	91.50	89.30	3.31	0.00	89.00	0.00
	VPM	0.00	0.00	0.00	2.48	97.50	97.00	7.43	0.00	97.00	0.00
	VPI	0.00	0.00	0.00	10.80	98.60	97.30	18.90	0.00	93.20	0.00
	VL	3.87	0.00	0.00	0.00	83.00	64.20	1.35	0.19	79.90	0.00
	VA	0.98	0.00	0.00	0.33	83.70	34.60	0.00	1.96	71.90	4.25
	VM	0.00	0.00	0.00	0.00	88.90	66.70	1.01	0.00	88.90	0.00

Supplementary Table 11: Percent Thalamus nuclei overlap with highly correlated (> ±0.2) combined topic-maps: The top part of the table shows nuclei overlap with the right thalamus and the bottom with the left thalamus. The first column represents thalamus major nuclei groups, and the second corresponds to group nuclei. Each column represents a functional network-specific percent overlap. The second row depicts the highly correlated topic names in number (the full names are mentioned in Figure 9's legend & Table S10c).

Right Thalamus nuclei percent overlap

Thalamus Group	nuclei	MV #1 Topic 43	MV #1 Topic 45	MV #1 Topic 48	MV #1 Topic 42	OV #2 Topic 43	OV #2 Topic 46	OV #2 Topic 41	LV #3 Topic 46	LV #3 Topic 20	LV #3 Topic 43	LV #3 Topic 41	LV #3 Topic 39	DMN #4 Topic 1	DMN #4 Topic 9	DMN #4 Topic 47	DMN #4 Topic 34	CB #5 Topic 3	CB #5 Topic 18	SM #6 Topic 18	SM #6 Topic 40	SM #6 Topic 12	AU #7 Topic 7	AU #7 Topic 38	AU #7 Topic 49	AU #7 Topic 47	EX #8 Topic 49	EX #8 Topic 21	EX #8 Topic 31	EX #8 Topic 17	RF #9 Topic 17	RF #9 Topic 21	RF #9 Topic 10	RF #9 Topic 33	LF #10 Topic 38	LF #10 Topic 35	LF #10 Topic 6	LF #10 Topic 23
Anterior	AD	0	5	0	0	0	0	0	0	0	0	0	0	0	0	0	0	100	0	0	0	0	0	0	0	0	0	0	2	0	0	0	0	86	0	0	0	0
	AM	0	0	0	0	0	0	0	0	0	0	0	0	0	0	0	0	100	0	0	10	0	0	0	0	0	0	0	3	0	0	0	0	81	0	43	0	49
	AV	0	6	0	0	0	0	0	0	0	0	0	0	0	0	0	0	97	4	4	2	0	0	0	0	0	0	0	6	0	0	0	0	65	0	12	0	18
	LD	0	18	0	0	0	0	0	0	0	0	0	0	0	0	0	0	82	14	14	0	7	0	0	0	0	0	0	0	1	1	0	3	58	0	0	0	0
MD	MD	0	4	0	0	0	0	0	0	0	0	0	0	0	0	0	0	100	28	28	0	0	0	0	0	0	0	0	3	1	1	0	0	99	0	1	0	2
intralaminar	CM	0	1	0	0	0	0	0	0	2	0	0	0	0	0	0	0	100	70	70	0	0	0	0	0	0	0	0	1	3	3	0	0	100	0	0	0	0
	Pf	0	0	0	0	0	0	0	0	0	0	0	0	0	0	0	0	99	38	38	0	0	0	0	0	0	0	1	2	9	9	1	0	99	0	0	0	0
	sPf	0	0	0	0	0	0	0	0	0	0	0	0	0	0	0	0	100	49	49	0	0	0	0	0	0	0	0	8	8	8	0	0	100	0	0	0	0
	CL	0	9	0	0	0	0	0	0	0	0	0	0	0	0	0	0	97	34	34	0	1	0	0	0	0	0	0	3	0	0	0	1	92	0	1	0	2
	CeM	0	0	0	0	0	0	0	0	0	0	0	0	0	0	0	0	100	12	12	2	0	0	0	0	0	0	1	6	5	5	1	0	98	0	16	0	16
midline	Pv	0	0	0	0	0	0	0	0	0	0	0	0	0	0	0	0	100	0	0	0	0	0	0	0	0	0	0	2	0	0	0	0	100	0	17	0	17
	MV	0	0	0	0	0	0	0	0	0	0	0	0	0	0	0	0	100	0	0	0	0	0	0	0	0	0	0	1	4	4	0	0	100	0	19	0	18
Hb	Hb	0	0	0	0	0	0	0	0	0	0	0	0	0	0	0	0	95	4	4	0	0	0	0	0	0	0	0	0	5	5	0	0	90	0	0	0	0
Li/SG	Li	0	0	0	0	0	0	4	0	0	0	4	0	0	0	0	12	97	25	25	0	0	0	0	0	0	0	0	0	2	2	0	0	88	0	0	0	0
	SG	0	4	0	2	0	0	14	0	13	0	14	0	0	0	0	39	100	34	34	0	0	0	0	0	0	0	0	0	0	0	0	0	78	0	0	0	0
posterior G	LP	0	14	0	0	0	0	0	0	7	0	0	0	0	0	0	11	98	62	62	0	7	0	0	0	0	0	0	0	0	0	0	2	79	0	0	0	0
	Po	1	6	0	3	1	0	5	0	32	1	5	0	0	0	0	30	100	60	60	0	0	0	0	0	0	0	0	0	0	0	0	0	85	0	0	0	0
	MGN	1	12	0	21	1	0	17	0	35	1	17	0	0	0	0	63	93	38	38	0	0	0	0	0	0	0	0	0	0	0	0	0	58	0	0	0	0
	LGN	1	15	0	54	1	0	12	0	36	1	12	0	0	0	0	91	57	18	18	0	0	0	0	0	0	0	0	0	0	0	0	0	14	0	0	0	0
Pulvinar	PuA	0	9	0	0	0	0	0	0	19	0	0	0	0	0	0	4	100	85	85	0	0	0	0	0	0	0	0	0	0	0	0	0	100	0	0	0	0
	Pul	1	20	0	63	1	0	7	0	41	1	7	0	0	0	0	94	50	17	17	0	1	0	0	0	0	0	0	0	0	0	0	0	13	0	0	0	0
	PuL	0	14	0	35	0	0	1	0	33	0	1	0	0	0	0	77	67	34	34	0	13	0	0	0	0	0	0	0	0	0	0	0	30	0	0	0	0
	PuM	0	5	0	16	0	0	3	0	10	0	3	0	0	0	0	50	74	23	23	0	4	0	0	0	0	0	0	0	0	0	0	1	41	0	0	0	0
Lateral	VPL	0	25	0	0	0	0	0	0	25	0	0	0	0	0	0	9	100	89	89	0	2	0	0	0	0	0	0	0	0	0	0	0	95	0	0	0	0
	VPM	0	9	0	0	0	0	0	0	8	0	0	0	0	0	0	2	100	82	82	0	0	0	0	0	0	0	0	3	4	4	0	0	100	0	0	0	0
	VPI	1	15	0	0	1	0	0	0	50	1	0	0	0	0	0	13	100	94	94	0	0	0	0	0	0	0	0	0	1	1	0	0	94	0	0	0	0
	VL	0	21	0	0	0	0	0	0	3	0	0	0	0	0	0	0	100	66	66	0	1	0	0	0	0	0	0	3	1	1	0	0	89	0	0	0	0
	VA	0	8	0	0	0	0	0	0	0	0	0	0	0	0	0	0	100	38	38	2	0	0	0	0	0	0	0	5	3	3	0	0	89	0	12	0	14
	VM	0	5	0	0	0	0	0	0	1	0	0	0	0	0	0	0	100	64	64	0	0	0	0	0	0	0	0	4	8	8	0	0	100	0	0	0	0

Left Thalamus nuclei percent overlap

Thalamus Group	nuclei	MV #1 Topic 43	MV #1 Topic 45	MV #1 Topic 48	MV #1 Topic 42	OV #2 Topic 43	OV #2 Topic 46	OV #2 Topic 41	LV #3 Topic 46	LV #3 Topic 20	LV #3 Topic 43	LV #3 Topic 41	LV #3 Topic 39	DMN #4 Topic 1	DMN #4 Topic 9	DMN #4 Topic 47	DMN #4 Topic 34	CB #5 Topic 3	CB #5 Topic 18	SM #6 Topic 18	SM #6 Topic 40	SM #6 Topic 12	AU #7 Topic 7	AU #7 Topic 38	AU #7 Topic 49	AU #7 Topic 47	EX #8 Topic 49	EX #8 Topic 21	EX #8 Topic 31	EX #8 Topic 17	RF #9 Topic 17	RF #9 Topic 21	RF #9 Topic 10	RF #9 Topic 33	LF #10 Topic 38	LF #10 Topic 35	LF #10 Topic 6	LF #10 Topic 23
Anterior	AD	0	0	0	0	0	0	0	0	0	0	0	0	0	0	0	0	100	1	1	0	0	0	0	0	0	0	0	0	0	0	0	0	77	0	0	0	0
	AM	0	0	0	0	0	0	0	0	0	0	0	0	0	0	0	0	100	0	0	2	0	0	0	0	0	0	0	0	0	0	0	0	67	0	22	0	31
	AV	0	4	0	0	0	0	0	0	0	0	0	0	0	0	0	0	99	12	12	0	0	0	0	0	0	0	0	0	2	2	0	2	59	0	5	0	10
	LD	0	5	0	0	0	0	0	0	0	0	0	0	0	0	0	0	96	42	42	0	0	0	0	1	0	1	0	0	0	0	0	1	70	0	0	0	0
MD	MD	0	2	0	0	0	0	0	0	0	0	0	0	0	0	0	0	100	44	44	0	0	0	0	0	0	0	0	0	0	0	0	0	98	0	0	0	1
intralaminar	CM	0	0	0	0	0	0	0	0	0	0	0	0	0	0	0	0	100	95	95	0	0	4	0	0	0	0	0	0	0	0	0	0	100	0	0	0	0
	Pf	0	0	0	0	0	0	0	0	0	0	0	0	0	0	0	0	100	65	65	0	0	1	0	0	0	0	0	0	1	1	0	0	100	0	0	0	0
	sPf	0	0	0	0	0	0	0	0	0	0	0	0	0	0	0	0	100	78	78	0	0	1	0	0	0	0	0	0	1	1	0	0	100	0	0	0	0
	CL	0	3	0	0	0	0	0	0	0	0	0	0	0	0	0	0	100	57	57	0	0	0	0	0	0	0	0	0	0	0	0	0	95	0	0	0	1
	CeM	0	0	0	0	0	0	0	0	0	0	0	0	0	0	0	0	100	21	21	1	0	0	0	0	0	0	0	0	1	1	0	0	98	0	10	0	11
midline	Pv	0	0	0	0	0	0	0	0	0	0	0	0	0	0	0	0	100	0	0	0	0	0	0	0	0	0	0	0	0	0	0	0	100	0	13	0	14
	MV	0	0	0	0	0	0	0	0	0	0	0	0	0	0	0	0	100	6	6	0	0	0	0	0	0	0	0	0	0	0	0	0	100	0	11	0	13
Hb	Hb	0	0	0	0	0	0	0	0	0	0	0	0	0	0	0	0	99	31	31	0	0	0	0	0	0	0	0	0	2	2	0	0	97	0	0	0	0
Li/SG	Li	0	0	0	0	0	0	0	0	0	0	0	0	0	0	0	18	100	58	58	0	0	5	0	0	0	0	0	0	1	1	0	0	98	0	0	0	0
	SG	0	0	0	1	0	0	0	0	0	0	0	0	0	0	0	46	99	59	59	0	0	11	0	0	0	0	0	0	0	0	0	0	87	0	0	0	0
posterior G	LP	0	2	0	0	0	0	0	0	0	0	0	0	0	0	0	0	100	96	96	0	7	0	0	0	0	0	0	0	0	0	0	1	90	0	0	0	0
	Po	0	0	0	1	0	0	0	0	0	0	0	0	0	0	0	32	100	79	79	0	0	10	0	0	0	0	0	0	0	0	0	0	91	0	0	0	0
	MGN	4	1	0	21	4	0	0	0	0	4	0	0	0	0	0	67	88	49	49	0	0	7	0	0	0	0	0	0	0	0	0	0	53	0	0	0	0
	LGN	4	4	0	47	4	0	0	0	0	4	0	0	0	0	0	92	59	21	21	0	0	0	0	0	0	0	0	0	0	0	0	0	10	0	0	0	0
Pulvinar	PuA	0	0	0	0	0	0	0	0	0	0	0	0	0	0	0	2	100	99	99	0	0	8	0	0	0	0	0	0	0	0	0	0	100	0	0	0	0
	Pul	5	3	0	62	5	0	0	0	0	5	0	0	0	0	0	94	58	18	18	0	0	0	0	0	0	0	0	0	0	0	0	0	12	0	0	0	0
	PuL	2	2	0	24	2	0	0	0	0	2	0	0	0	0	0	54	87	48	48	0	1	0	0	0	0	0	0	0	0	0	0	0	38	0	0	0	0
	PuM	1	1	0	10	1	0	0	0	0	1	0	0	0	0	0	39	91	44	44	0	0	0	0	0	0	0	0	0	0	0	0	0	60	0	0	0	0
Lateral	VPL	0	3	0	0	0	0	0	0	0	0	0	0	0	0	0	4	100	98	98	0	3	3	0	0	0	0	0	0	0	0	0	1	96	0	0	0	0
	VPM	0	0	0	0	0	0	0	0	0	0	0	0	0	0	0	3	100	100	100	0	0	8	0	0	0	0	0	0	0	0	0	0	100	0	0	0	0
	VPI	1	0	0	0	1	0	0	0	0	1	0	0	0	0	0	12	100	99	99	0	0	19	0	0	0	0	0	0	0	0	0	0	93	0	0	0	0
	VL	0	5	0	0	0	0	0	0	0	0	0	0	0	0	0	0	100	77	77	0	1	1	0	0	0	0	0	0	0	0	0	4	92	0	0	0	0
	VA	0	1	0	0	0	0	0	0	0	0	0	0	0	0	0	1	100	43	43	0	0	0	0	0	0	0	0	0	4	4	0	9	79	0	3	0	4
	VM	0	0	0	0	0	0	0	0	0	0	0	0	0	0	0	0	100	77	77	0	0	1	0	0	0	0	0	0	0	0	0	0	100	0	0	0	0

Supplementary Table 12: Percent Thalamus nuclei overlap with highly correlated (> ±0.2) each topic-map: The top part of the table shows nuclei overlap with the right thalamus and the bottom with the left thalamus. The first column represents thalamus major nuclei groups, and the second corresponds to group nuclei. The similar colored columns represent a functional network. The third row, below each column, depicts the highly correlated topic name in number (the full names are mentioned in Figure 9's legend & Table S10c). The percent overlap of the corresponding thalamic nuclei is down to the topic name. Figure S6a-j visualizes the given percent overlaps.

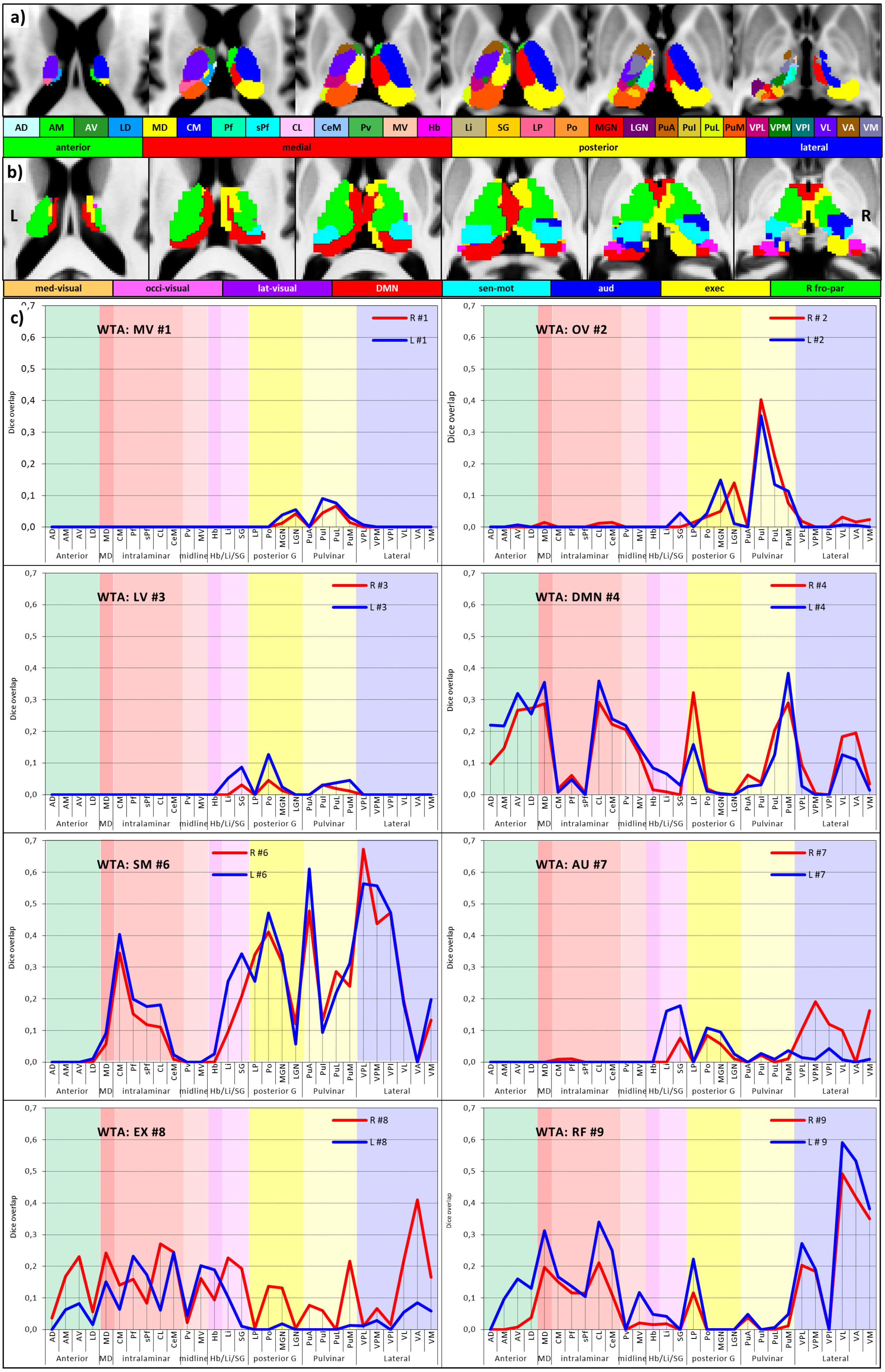

a)
AD
AM
AV
LD
MD
CM
Pf
sPf
CL
CeM
Pv
MV
Hb
Li
SG
LP
Po
MGN
LGN
PuA
Pul
PuL
PuM
VPL
VPM
VPI
VL
VA
VM
anterior
medial
posterior
lateral
b)
L
R
med-visual
occi-visual
lat-visual
DMN
sen-mot
aud
exec
R fro-par
c)
WTA: MV #1
R #1
L #1
WTA: OV #2
R # 2
L #2
WTA: LV #3
R #3
L #3
WTA: DMN #4
R #4
L #4
WTA: SM #6
R #6
L #6
WTA: AU #7
R #7
L #7
WTA: EX #8
R #8
L #8
WTA: RF #9
R #9
L # 9
Dice overlap
0,0
0,1
0,2
0,3
0,4
0,5
0,6
0,7
Anterior
MD
intralaminar
midline
Hb/Li/SG
posterior G
Pulvinar
Lateral

Supplementary Figure 1:Anatomical Overlap with Thalamic Histology - Winner-takes-all Cortico-thalamic Connectivity of RSN: a) Depiction of thalamic nuclei (left) and nuclei groups (right) of the Morel atlas six axial views. b) Corresponding WTA maps overlaid on the left and right thalamus template. c) Graphical display of WTA Dice overlap for the right (red) and left (blue) thalamic nuclei.

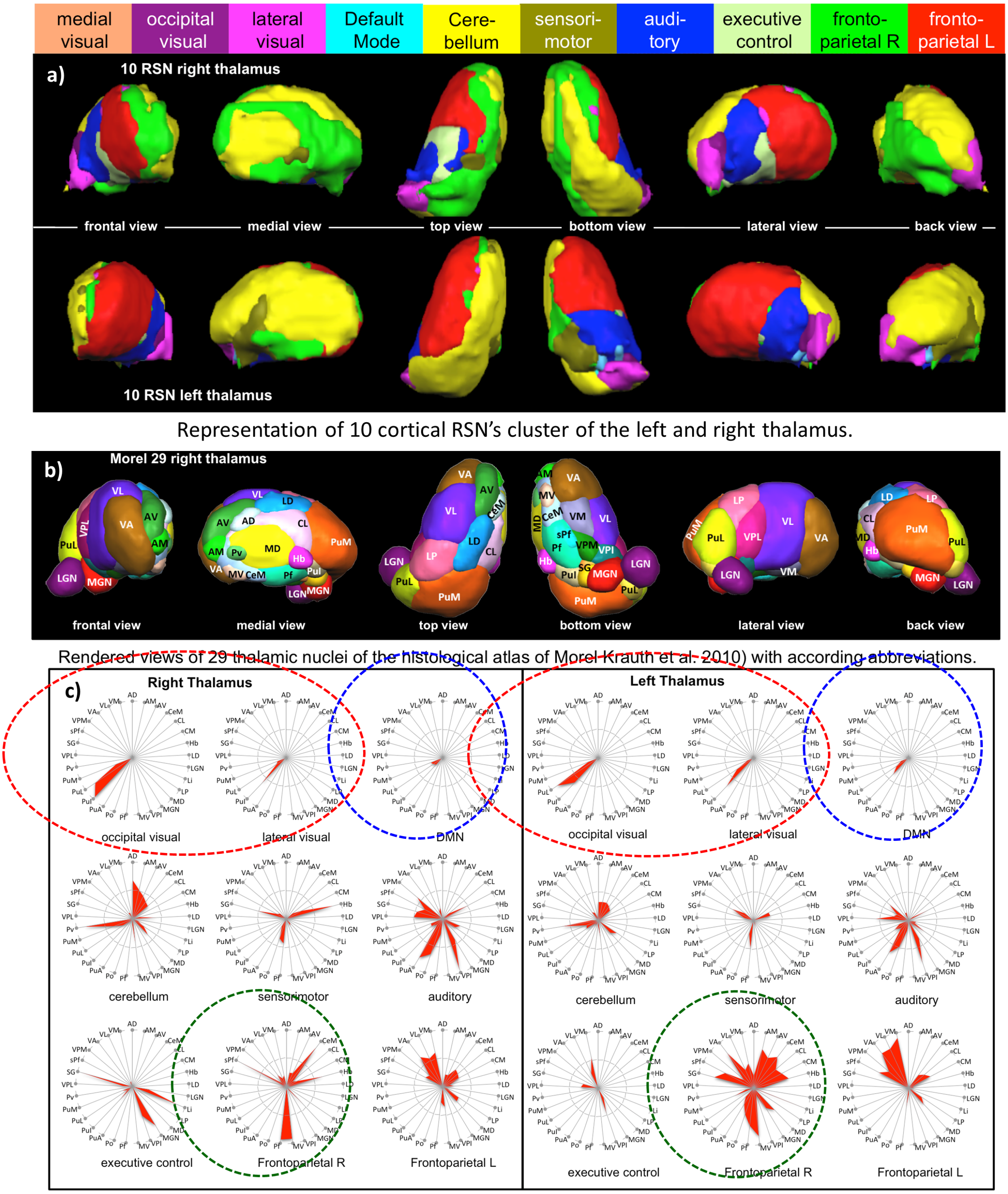

Supplementary Figure 2: Winner-takes-all cortico-thalamic RSN connectivity including the cerebellum: a) Representation of functional network-specific WTA clusters of the left and right thalamus. Note: The determined thalamic maps of 10 c-RSNs showed an ordered arrangement within the left and right thalamus – revealing a spatially variable communication with different cortical networks as well as hemispheric differences! **b)** Anatomical parcellation of Thalamic Histology; **c)** Graphical display of thalamic overlay with WTA clusters. Note: The left and right thalamus results indicate a nice coverall convergence. For example, known correspondence exists between Pulvinar and Visual networks (red dashed circles)! In contrast, frontoparietal networks employ several nuclei at rest (green dashed circles), while the Default mode networks mainly seem to be confined to cortico-cortical processing at rest (blue dashed circles).

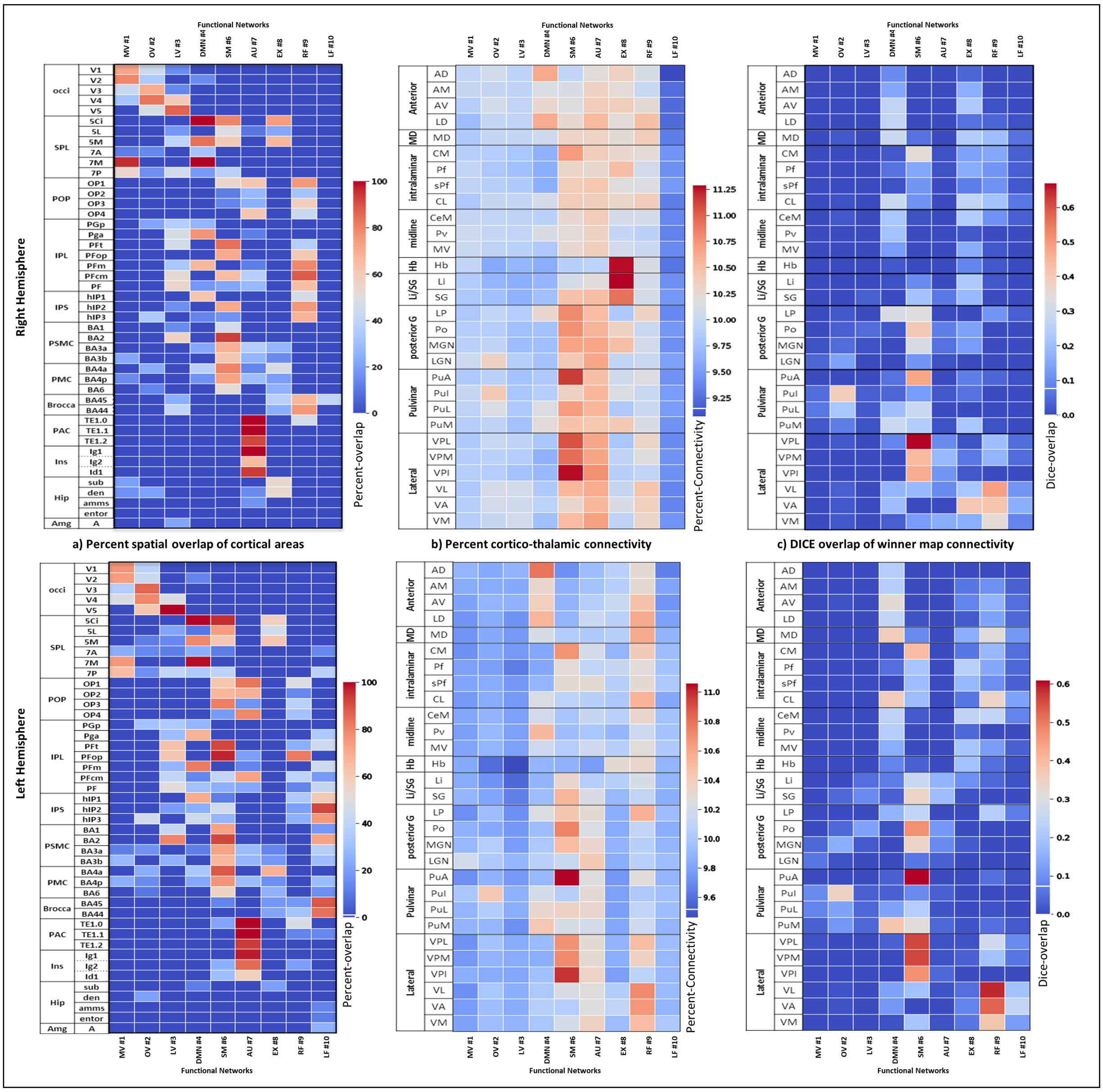

Supplementary Figure 3: Comparison of cortical areas, connectivity, and winner maps of the right and left hemisphere RSN: a) Cortical Areas in percent spatial overlap with the Jülich Histology Atlas. b) Percent connectivity with thalamic nuclei. c) DICE overlap of winner map connectivity with thalamic nuclei.

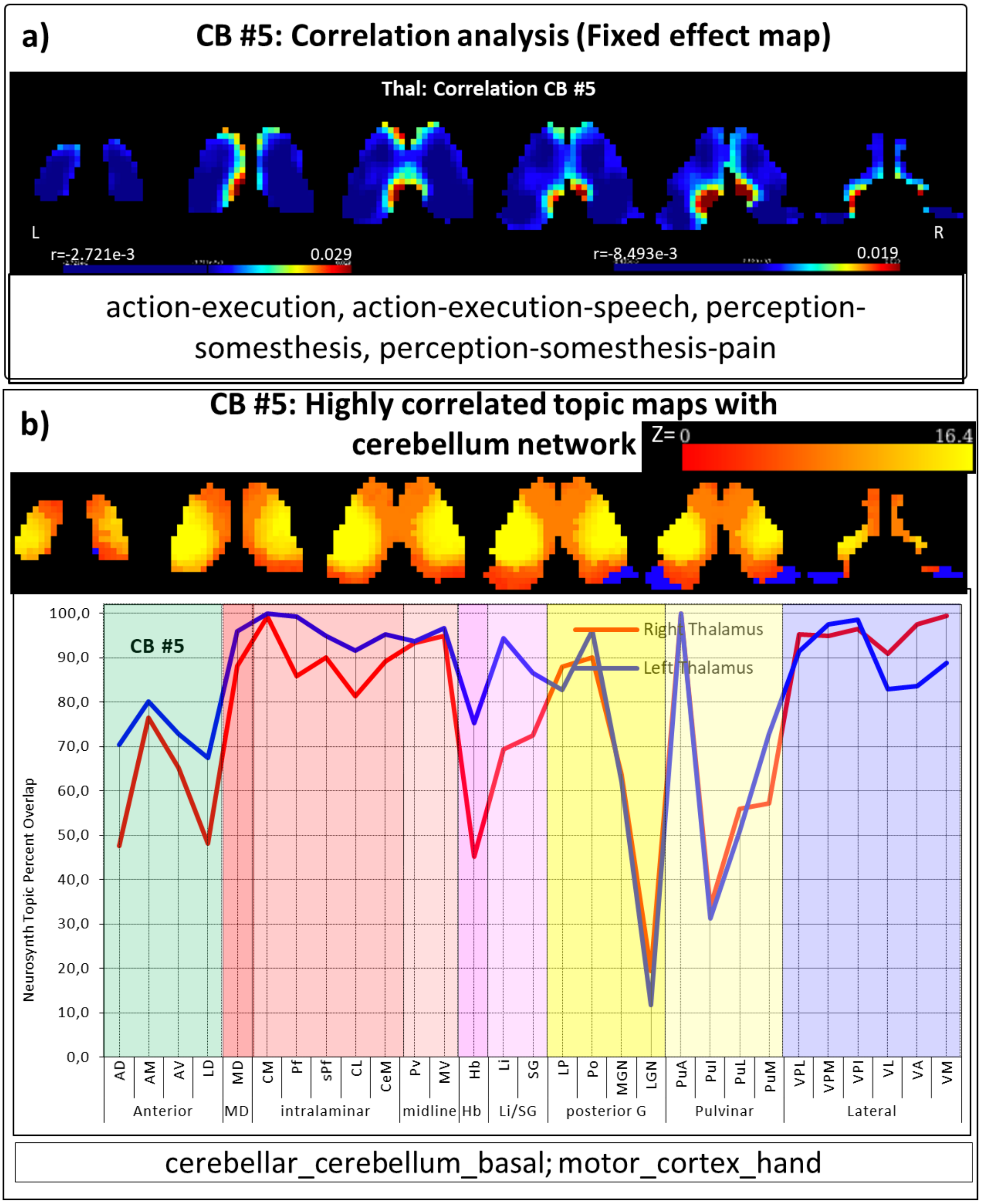

Supplementary Figure 4: a) Cortico-thalamic Connectivity of the Cerebellum CB RSN**#5:** Top: *Corresponding* correlations maps of the thalamus (top). See Table S4 in the Supplement. See Table S5 for the Nuclei names. The scales in the fixed effect are not visible due to small size of color bar. Scale: *CB: Left -2.721e-3 – 0.029, right -8.493e-3 – 0.019* **b) Highly correlated topic-maps with #CB 5 (top) and percentage overlap with thalamic** nuclei (bottom): highly correlated topic-maps (spatially overlaid on six different axial slices; same as the previous figure 9 depicting correlation maps with CB network. The topic maps were thresholded at z-value 3.1 (p-value 0.001). The graph within each subplot depicts the percentage nuclei overlap of highly correlated topic maps (marked in a red circle in Figure 8) with the thalamus. CB #5: 3 cerebellar_cerebellum_basal; 18 motor_cortex_hand. Color scale: same for all the maps (0 – 16.4) across all the networks. The blue color depicts thalamus mask in the background.

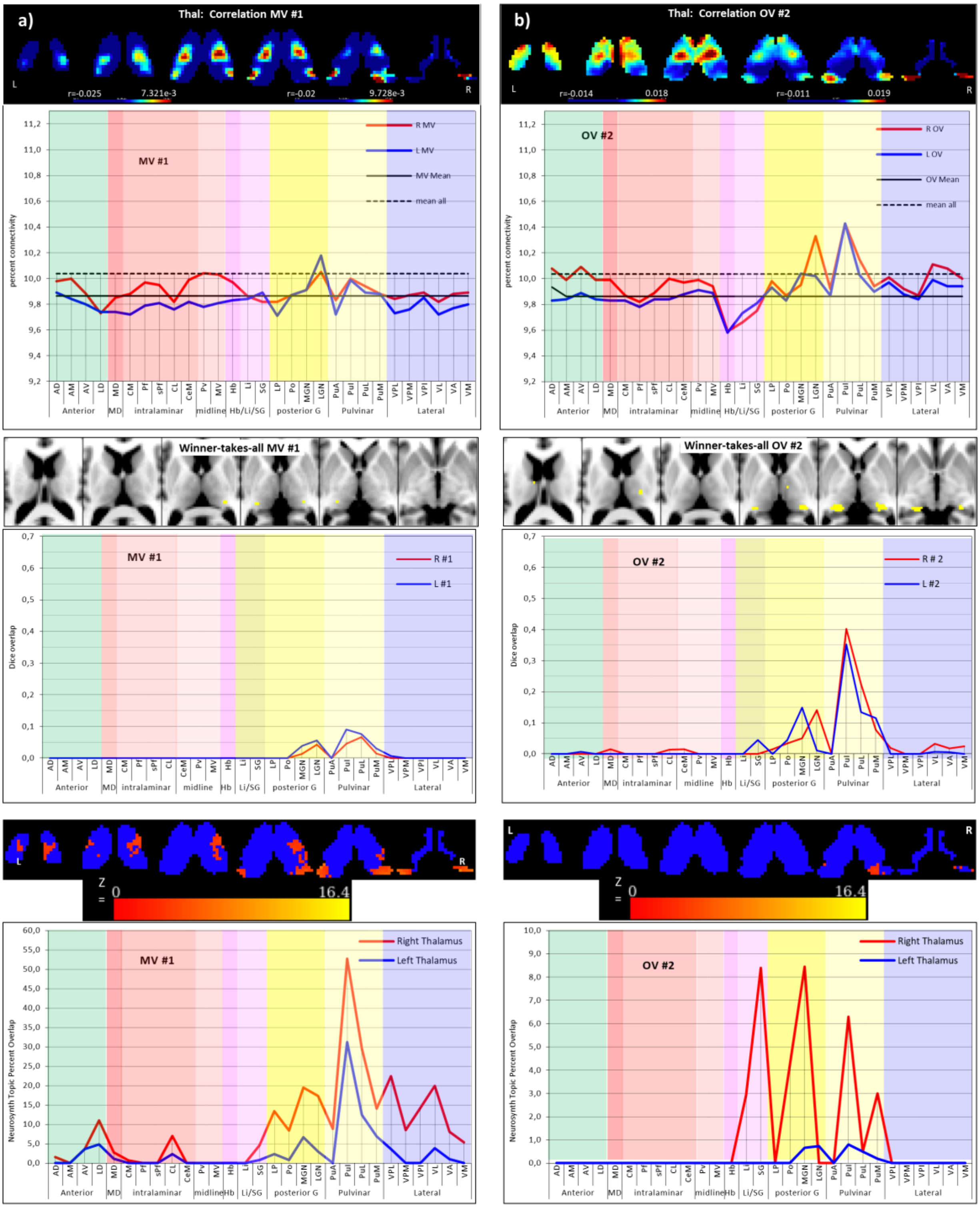

Supplementary Figure 5: Cortico-thalamic Connectivity and Topic maps of MV, and OV RSN: 1st Subplot: Correlation maps of the thalamus and mean correlation coefficients. **2nd Subplot:** WTA maps with axial views of the thalamus and corresponding dice overlap with thalamic nuclei. **3rd Subplot:** Results of Topic Mapping Abbreviations: see Table S4 in the Supplement. See Table S5 for the Nuclei names.

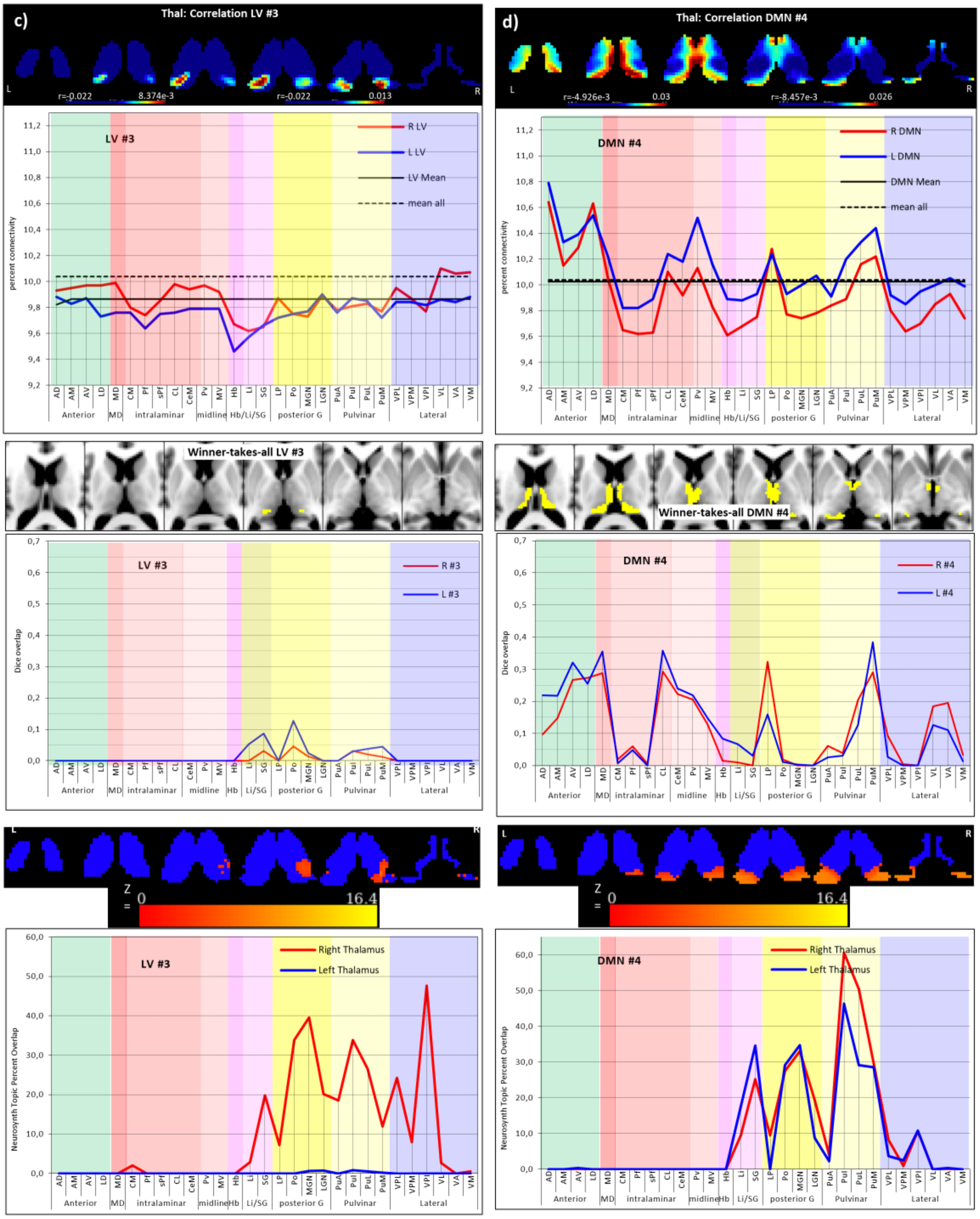

Supplementary Figure 6: Cortico-thalamic Connectivity and Topic maps of LV, and DMN: 1st Subplot: Correlation maps of the thalamus and mean correlation coefficients. **2nd Subplot:** WTA maps with axial views of the thalamus and corresponding dice overlap with thalamic nuclei. **3rd Subplot:** Results of Topic Mapping Abbreviations: see Table S4 in the Supplement. See Table S5 for the Nuclei names.

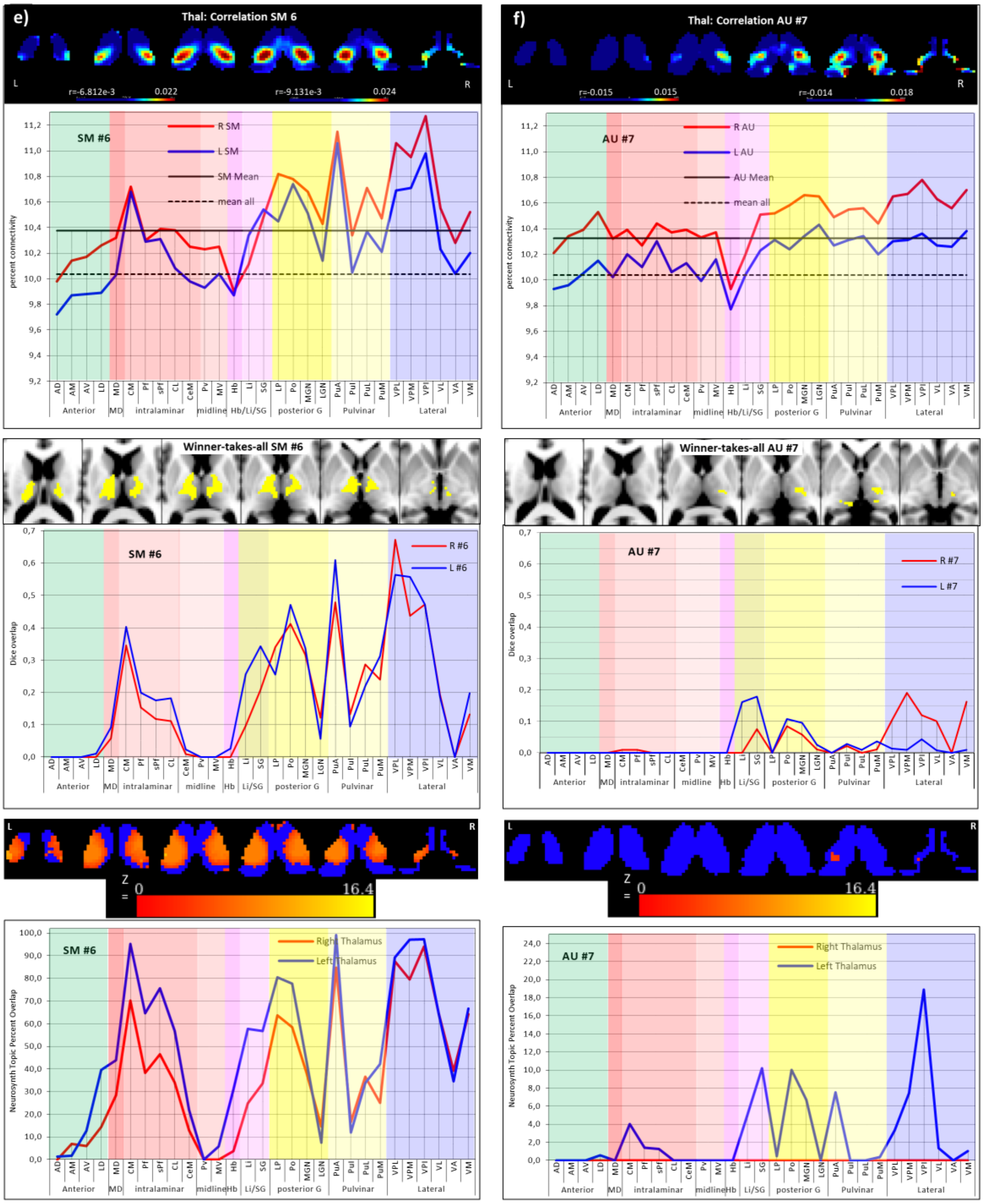

Supplementary Figure 7: Cortico-thalamic Connectivity and Topic maps of SM, and AU: 1st Subplot: Correlation maps of the thalamus and mean correlation coefficients. **2nd Subplot:** WTA maps with axial views of the thalamus and corresponding dice overlap with thalamic nuclei. **3rd Subplot:** Results of Topic Mapping Abbreviations: see Table S4 in the Supplement. See Table S5 for the Nuclei names.

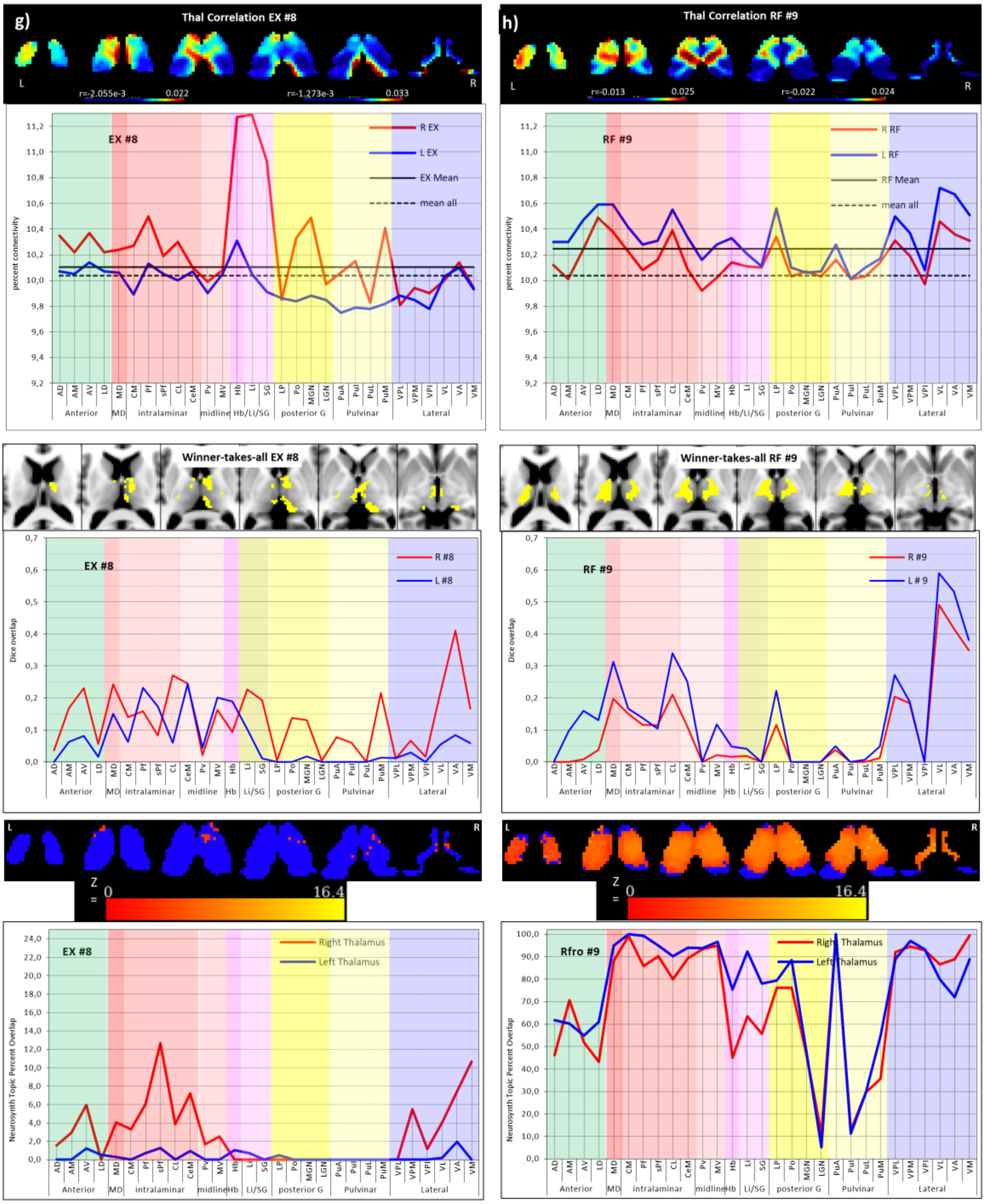

Supplementary Figure 8: Cortico-thalamic Connectivity and Topic maps of , and AU: 1st Subplot: Correlation maps of the thalamus and mean correlation coefficients. **2nd Subplot:** WTA maps with axial views of the thalamus and corresponding dice overlap with thalamic nuclei. **3rd Subplot:** Results of Topic Mapping Abbreviations: see Table S4 in the Supplement. See Table S5 for the Nuclei names.

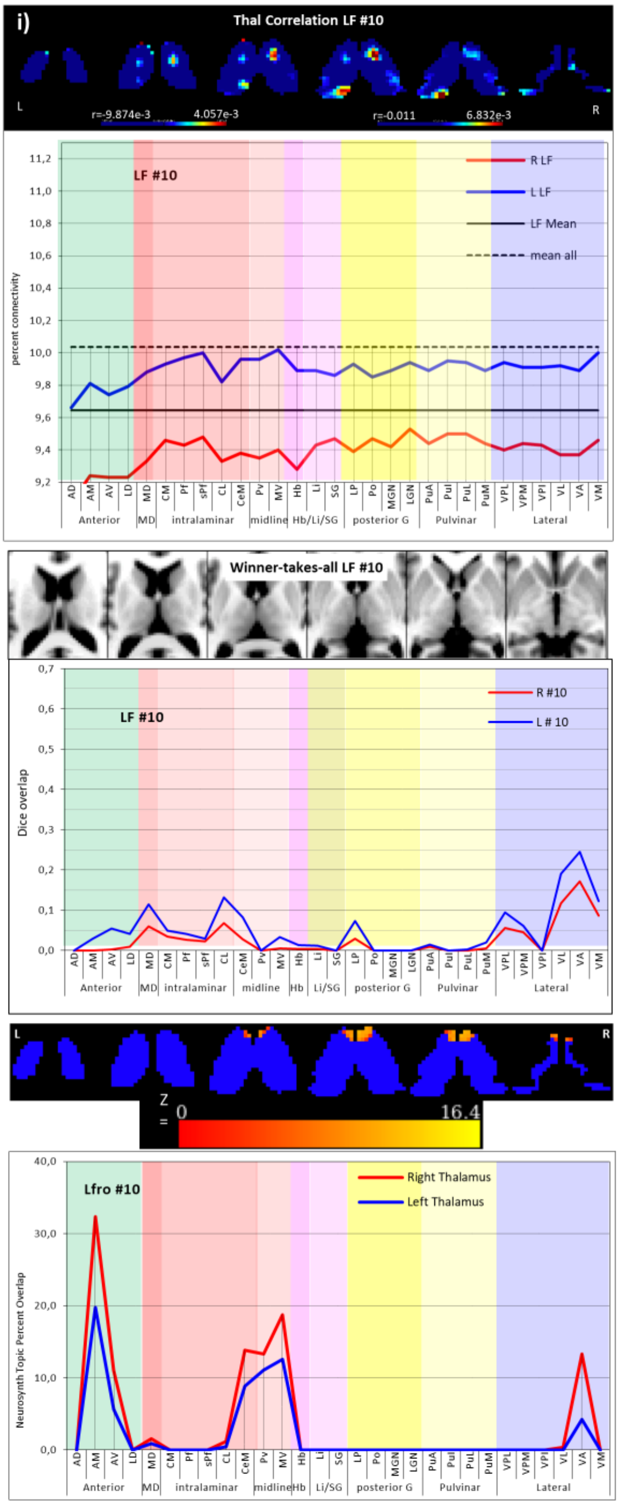

Supplementary Figure 9: Cortico-thalamic Connectivity and Topic maps of LF: 1st Subplot: Correlation maps of the thalamus and mean correlation coefficients. **2nd Subplot:** WTA maps with axial views of the thalamus and corresponding dice overlap with thalamic nuclei. **3rd Subplot:** Results of Topic Mapping Abbreviations: see Table S4 in the Supplement. See Table S5 for the Nuclei names.

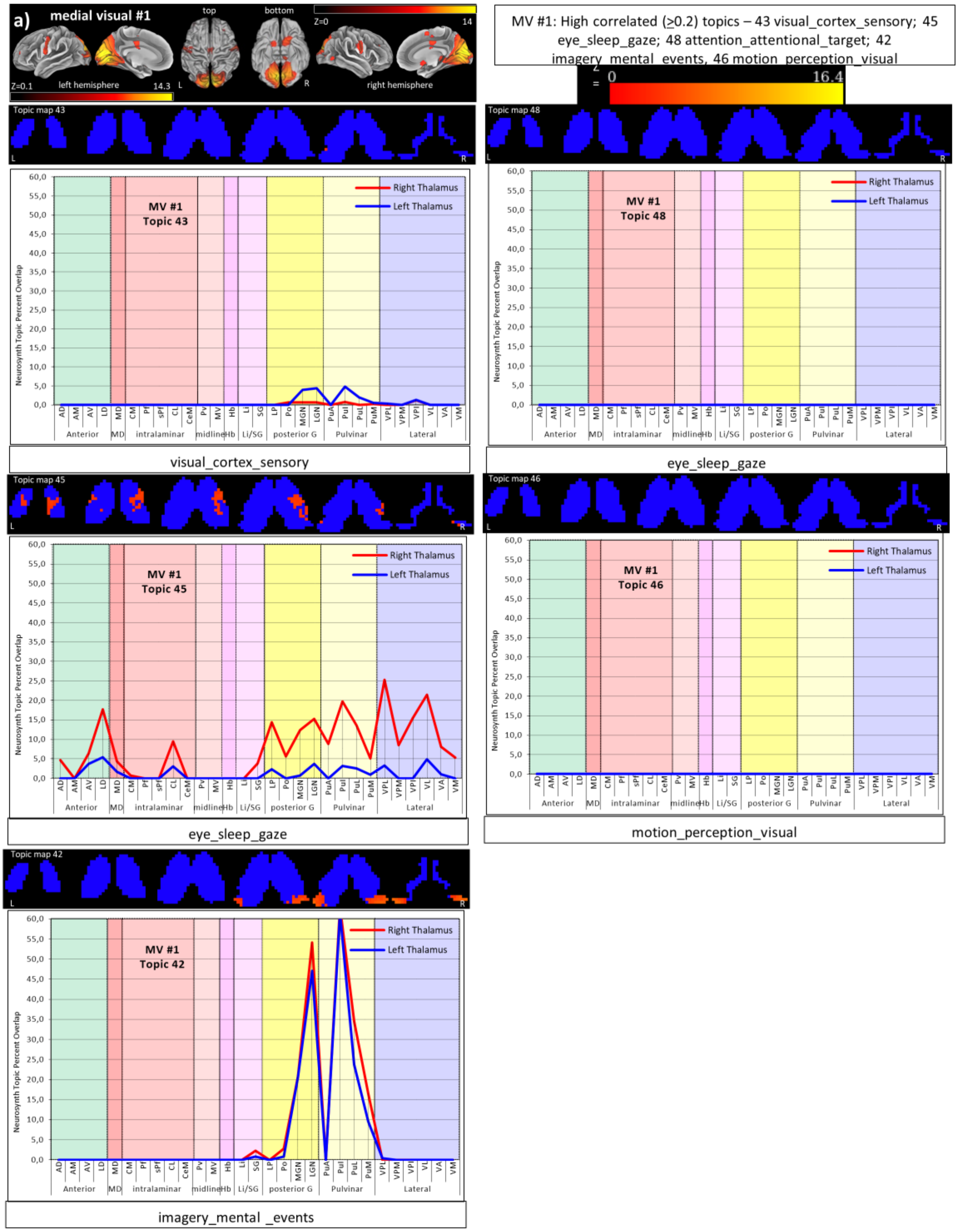

Supplementary Figure 10: MV #1 highly correlated (>0.2) **neurosynth** topic-maps within Thalamus: each subplot represents a high-correlated topic-map (spatially overlaid on six different axial slices; same as the previous figures 3-5 depicting correlation maps with each network). The topic maps were thresholded at z-value 3.1 (p-value 0.001). The graph within each subplot depicts the percent nuclei overlap of highly correlated topic maps separately (marked in the red circle in Figure 9) with Thalamus. **MV #1**: 43 visual_cortex_sensory; 45 eye_sleep_gaze; 48 attention_attentional_target; 45_motion_perception_visual, 42 imagery_mental_events. Note: no corresponding spatial map within the thalamus was found in topic 48, and 46. Top row shows the corresponding MV network brainmap, used for the neurosynth topic decoding. Color scale: same for all the maps (0 – 16.4) across all the networks. The blue color depicts thalamus mask in the background.

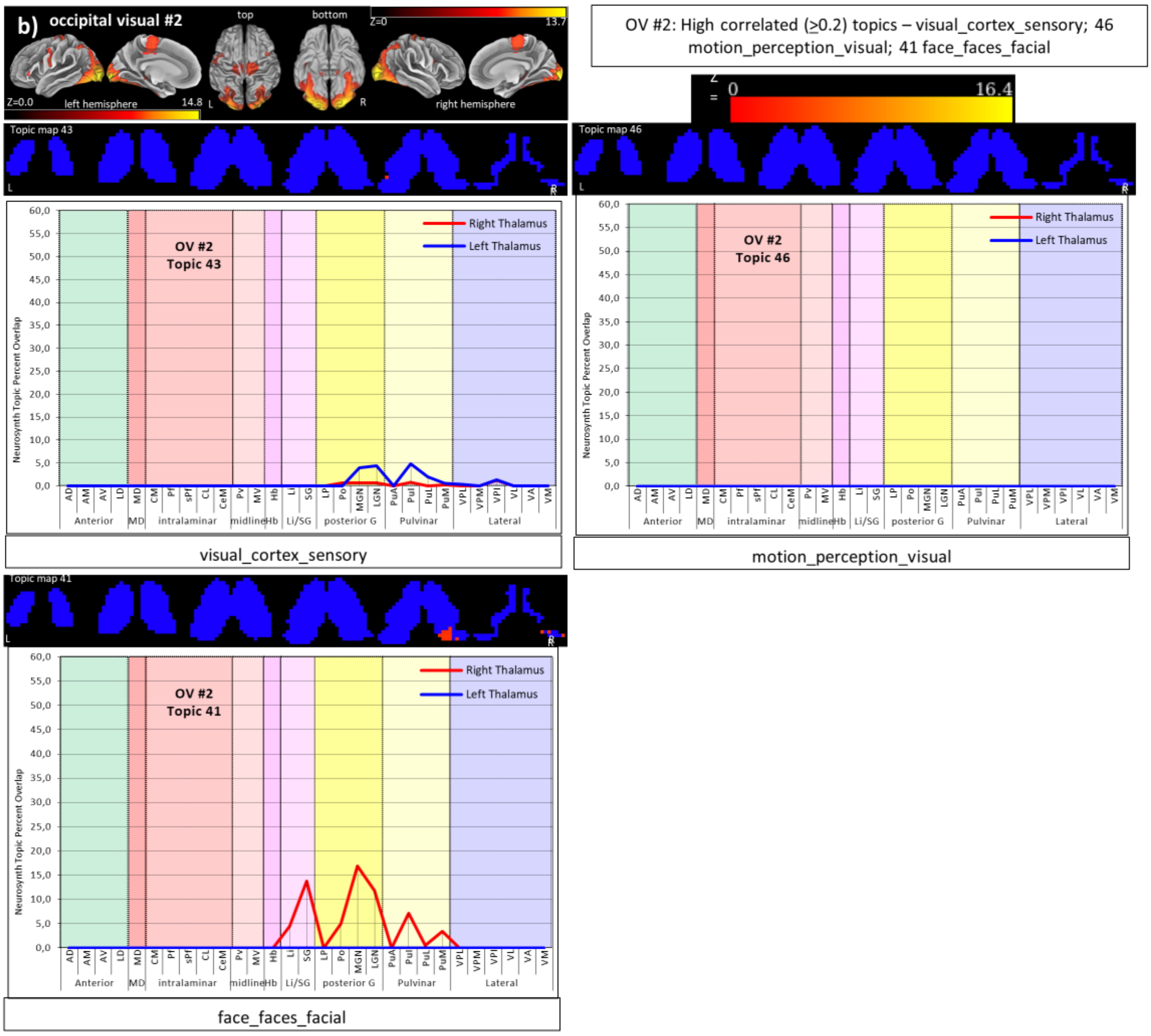

Supplementary Figure 11: OV #2 highly correlated (≥0.2) **neurosynth** topic-maps within Thalamus: each subplot represents a high-correlated topic-map (spatially overlaid on six different axial slices; same as the previous figures 3-5 depicting correlation maps with each network). The topic maps were thresholded at z-value 3.1 (p-value 0.001). The graph within each subplot depicts the percent nuclei overlap of highly correlated topic maps separately (marked in the red circle in Figure 9) with Thalamus. OV #2: 43 visual_cortex_sensory; 46 motion_perception_visual; 41 face_faces_facial. Note: no corresponding spatial map within the thalamus was found in topic 46. Top row shows the corresponding OV network brainmap, used for the neurosynth topic decoding. Color scale: same for all the maps (0 – 16.4) across all the networks. The blue color depicts thalamus mask in the background.

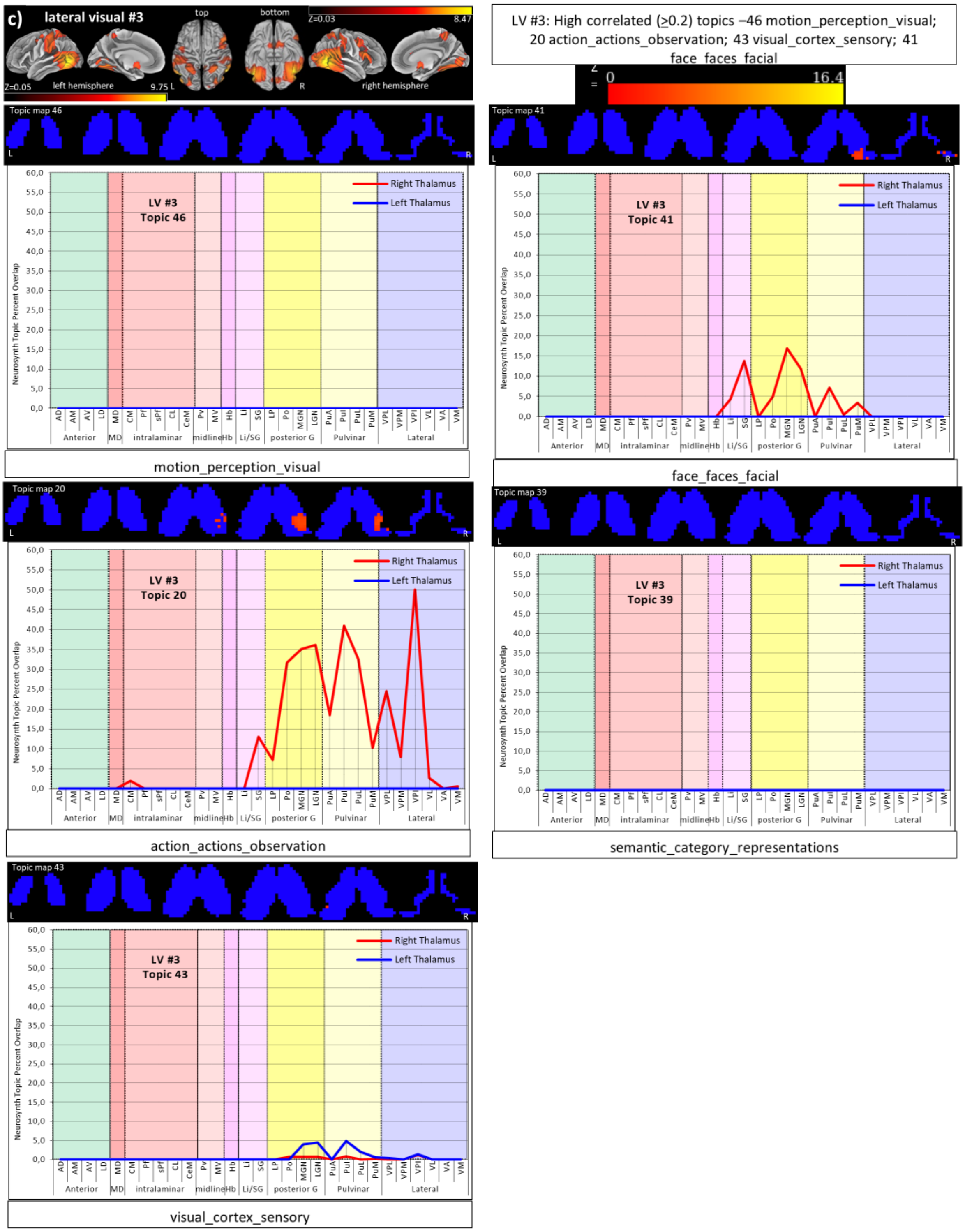

Supplementary Figure 12: LV #3 highly correlated (>0.2) **neurosynth** topic-maps within Thalamus: each subplot represents a high-correlated topic-map (spatially overlaid on six different axial slices; same as the previous figures 3-5 depicting correlation maps with each network). The topic maps were thresholded at z-value 3.1 (p-value 0.001). The graph within each subplot depicts the percent nuclei overlap of highly correlated topic maps separately (marked in the red circle in Figure 9) with Thalamus.

LV #3: 46 motion_perception_visual; 20 action_actions_observation; 43 visual_cortex_sensory; 41 face_faces_facial; 39 semantic_category_representations. Note: no corresponding spatial map within the thalamus was found in topic 46 and 39. Top row shows the LV network brainmap, used for the neurosynth topic decoding. Color scale: same for all the maps (0 – 16.4) across all the networks. The blue color depicts thalamus mask in the background.

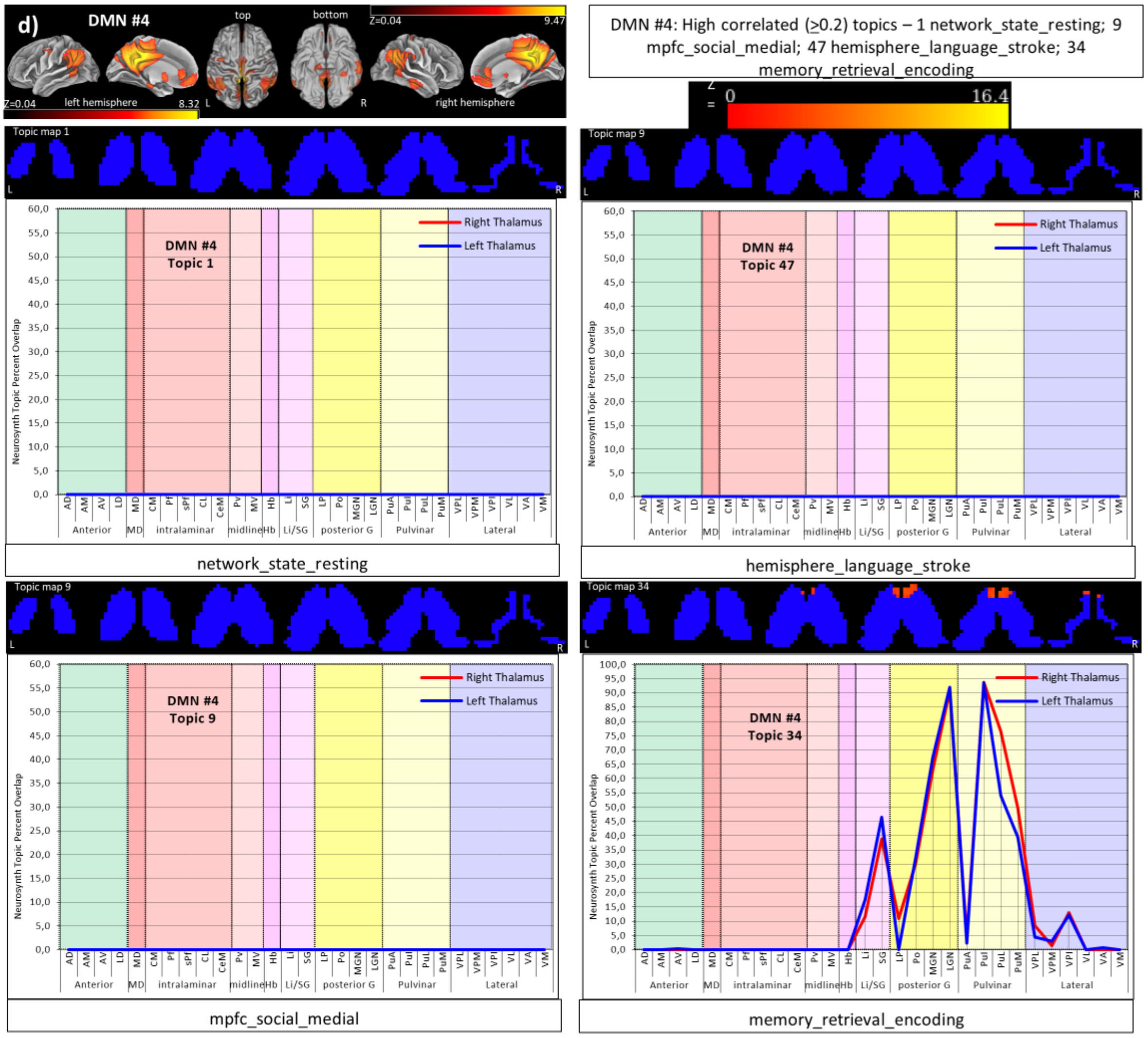

Supplementary Figure 13: DMN #4 highly correlated (≥0.2) **neurosynth** topic-maps within Thalamus: each subplot represents a high-correlated topic-map (spatially overlaid on six different axial slices; same as the previous figures 3-5 depicting correlation maps with each network). The topic maps were thresholded at z-value 3.1 (p-value 0.001). The graph within each subplot depicts the percent nuclei overlap of highly correlated topic maps separately (marked in the red circle in Figure 9) with Thalamus. DMN #4: 1 network_state_resting; 9 mpfc_social_medial; 47 hemisphere_language_stroke; 34 memory_retrieval_encoding. Note: no corresponding spatial map within the thalamus was found in topic 1, 9, and 47. Top row shows the DMN brainmap used for the neurosynth topic decoding. Color scale: same for all the maps (0 – 16.4) across all the networks. The blue color depicts thalamus mask in the background.

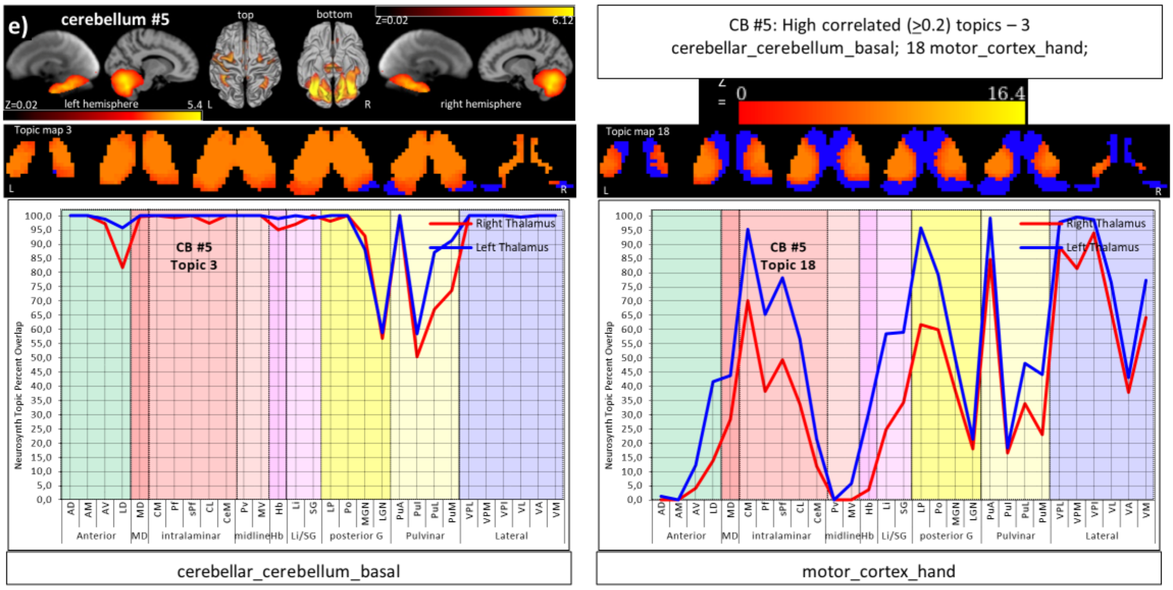

Supplementary Figure 14: CB #5 highly correlated (≥0.2) **neurosynth** topic-maps within Thalamus: each subplot represents a high-correlated topic-map (spatially overlaid on six different axial slices; same as the previous figures 3-5 depicting correlation maps with each network). The topic maps were thresholded at z-value 3.1 (p-value 0.001). The graph within each subplot depicts the percent nuclei overlap of highly correlated topic maps separately (marked in the red circle in Figure 9) with Thalamus. CB #5: 3 cerebellar_cerebellum_basal; 18 motor_cortex_hand. Top row shows the corresponding cerebellum network, used for the neurosynth topic decoding. Color scale: same for all the maps (0 – 16.4) across all the networks. The blue color depicts thalamus mask in the background.

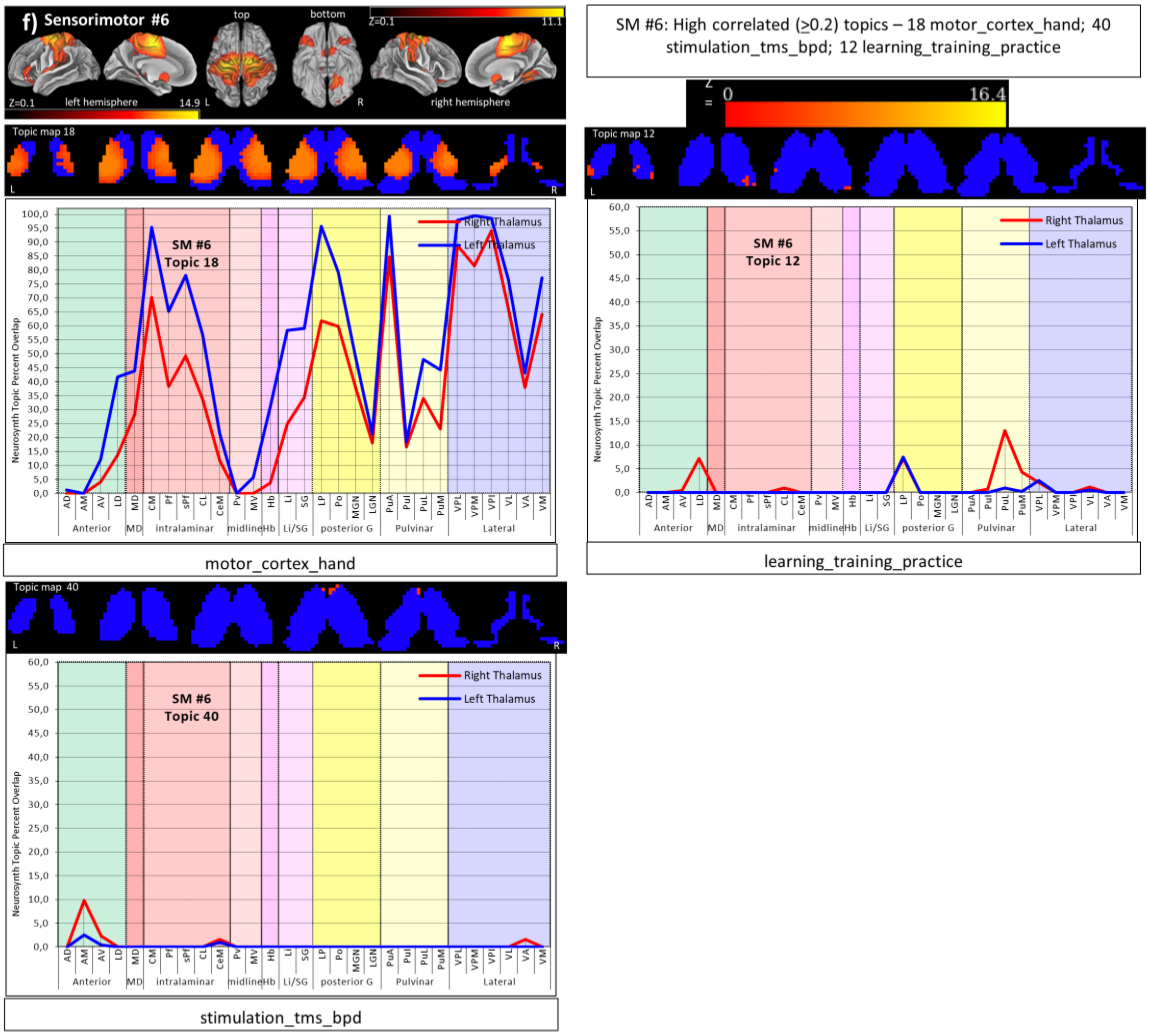

Supplementary Figure 15: SM #6 highly correlated (≥0.2) **neurosynth** topic-maps within Thalamus: each subplot represents a high-correlated topic-map (spatially overlaid on six different axial slices; same as the previous figures 3-5 depicting correlation maps with each network). The topic maps were thresholded at z-value 3.1 (p-value 0.001). The graph within each subplot depicts the percent nuclei overlap of highly correlated topic maps separately (marked in the red circle in Figure 9) with Thalamus.

SM #6: 18 motor_cortex_hand; 40 stimulation_tms_bpd; 12 learning_training_practice. Top row shows the corresponding SM network brainmap, used for the neurosynth topic decoding. Color scale: same for all the maps (0 – 16.4) across all the networks. The blue color depicts thalamus mask in the background.

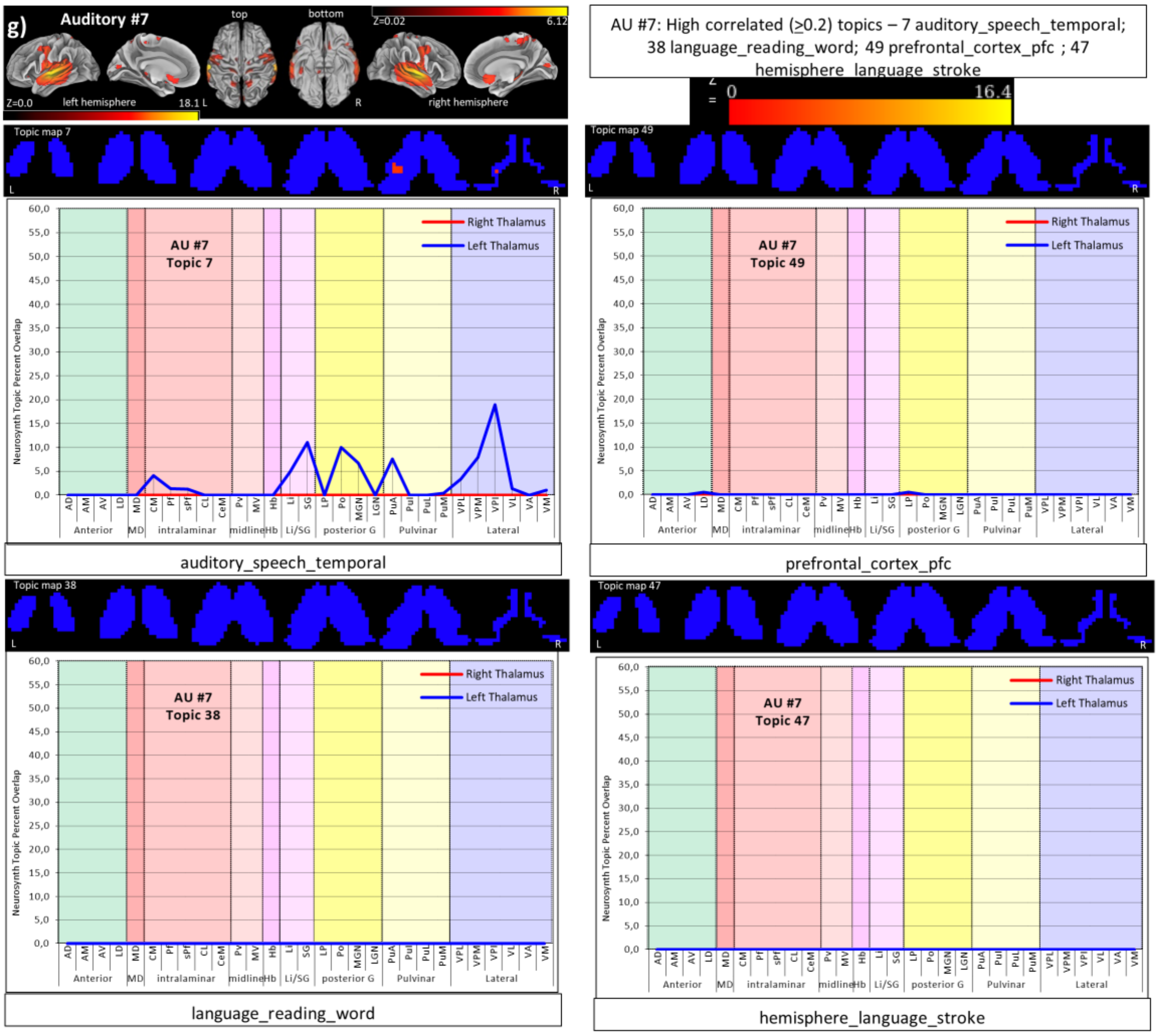

Supplementary Figure 16: AU #7 highly correlated (≥0.2) **neurosynth** topic-maps within Thalamus: each subplot represents a high-correlated topic-map (spatially overlaid on six different axial slices; same as the previous figures 3-5 depicting correlation maps with each network). The topic maps were thresholded at z-value 3.1 (p-value 0.001). The graph within each subplot depicts the percent nuclei overlap of highly correlated topic maps separately (marked in the red circle in Figure 9) with Thalamus. AU #7: 7 auditory_speech_temporal; 38 language_reading_word; 49 prefrontal_cortex_pfc ; 47 hemisphere_language_stroke. Note: no corresponding spatial map within the thalamus was found in topic 38, 49, and 47. Top row shows the corresponding AU network brainmap, used for the neurosynth topic decoding. Color scale: same for all the maps (0 – 16.4) across all the networks. The blue color depicts thalamus mask in the background.

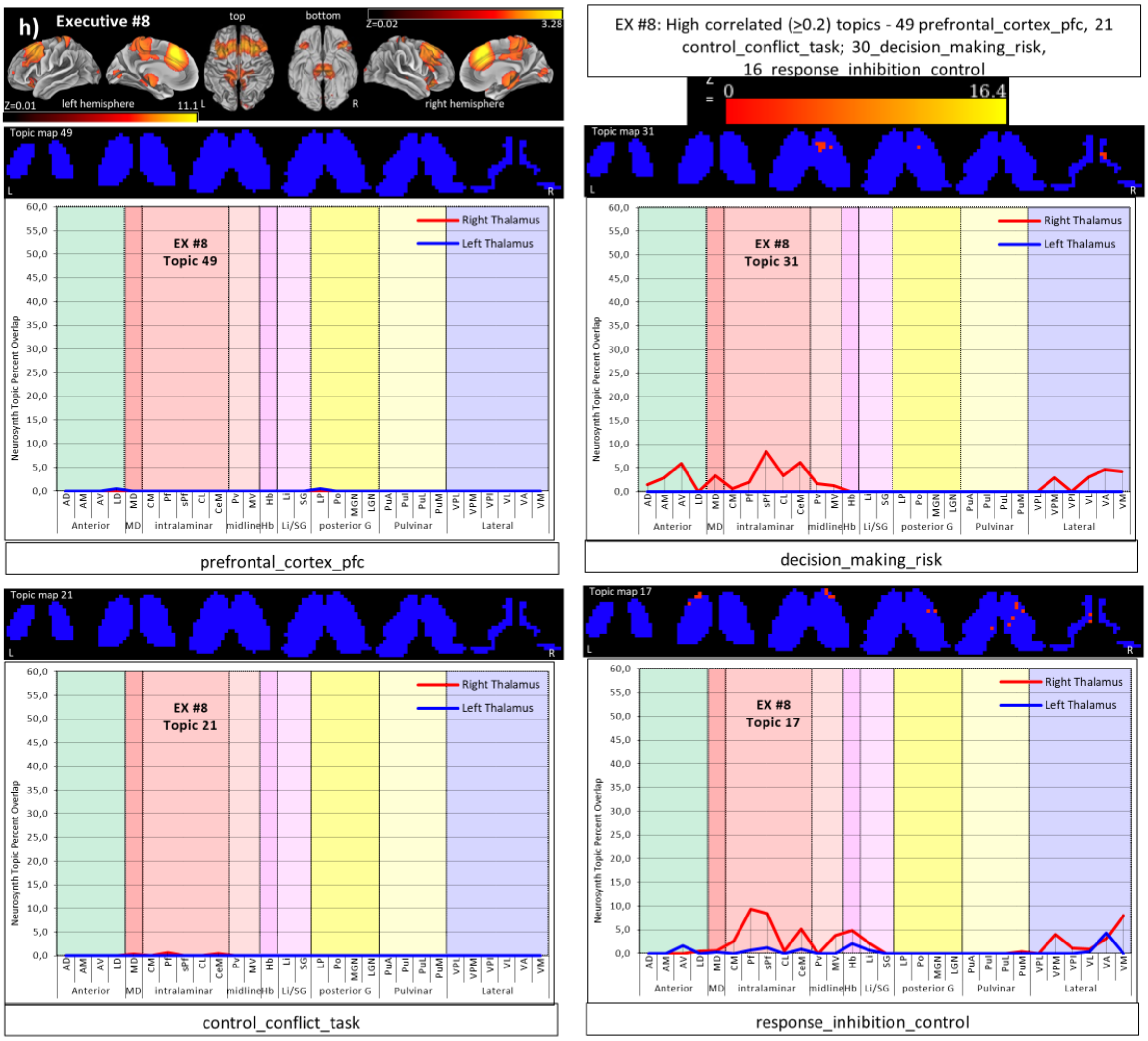

Supplementary Figure 17: EX #8 highly correlated (≥0.2) **neurosynth** topic-maps within Thalamus: each subplot represents a high-correlated topic-map (spatially overlaid on six different axial slices; same as the previous figures 3-5 depicting correlation maps with each network). The topic maps were thresholded at z-value 3.1 (p-value 0.001). The graph within each subplot depicts the percent nuclei overlap of highly correlated topic maps separately (marked in the red circle in Figure 9) with Thalamus. EX #8: 49 prefrontal_cortex_pfc, 21 control_conflict_task; 30_decision_making_risk, 16_response_inhibition_control. Note: no corresponding spatial map within the thalamus was found in topic 49, and 21. Top row shows the corresponding EX network brainmap, used for the neurosynth topic decoding. Color scale: same for all the maps (0 – 16.4) across all the networks. The blue color depicts thalamus mask in the background.

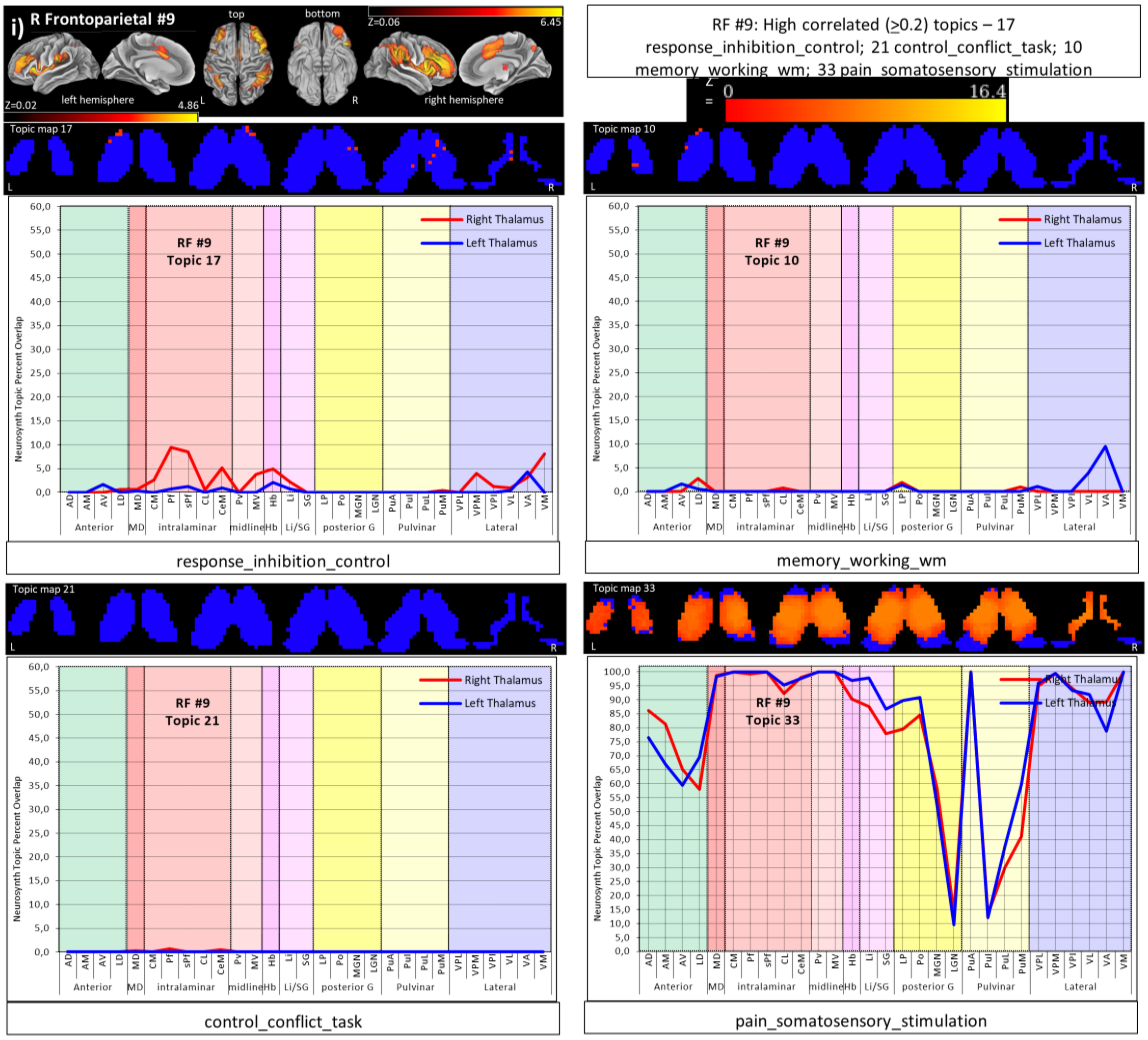

Supplementary Figure 18: RF #9 highly correlated (≥0.2) **neurosynth** topic-maps within Thalamus: each subplot represents a high-correlated topic-map (spatially overlaid on six different axial slices; same as the previous figures 3-5 depicting correlation maps with each network). The topic maps were thresholded at z-value 3.1 (p-value 0.001). The graph within each subplot depicts the percent nuclei overlap of highly correlated topic maps separately (marked in the red circle in Figure 9) with Thalamus. Rfro #9: 17 response_inhibition_control; 21 control_conflict_task; 10 memory_working_wm; 33 pain_somatosensory_stimulation. Note: no corresponding spatial map within the thalamus was found in topic 21. Top row shows the corresponding RF network brainmap, used for the neurosynth topic decoding. Color scale: same for all the maps (0 – 16.4) across all the networks. The blue color depicts thalamus mask in the background.

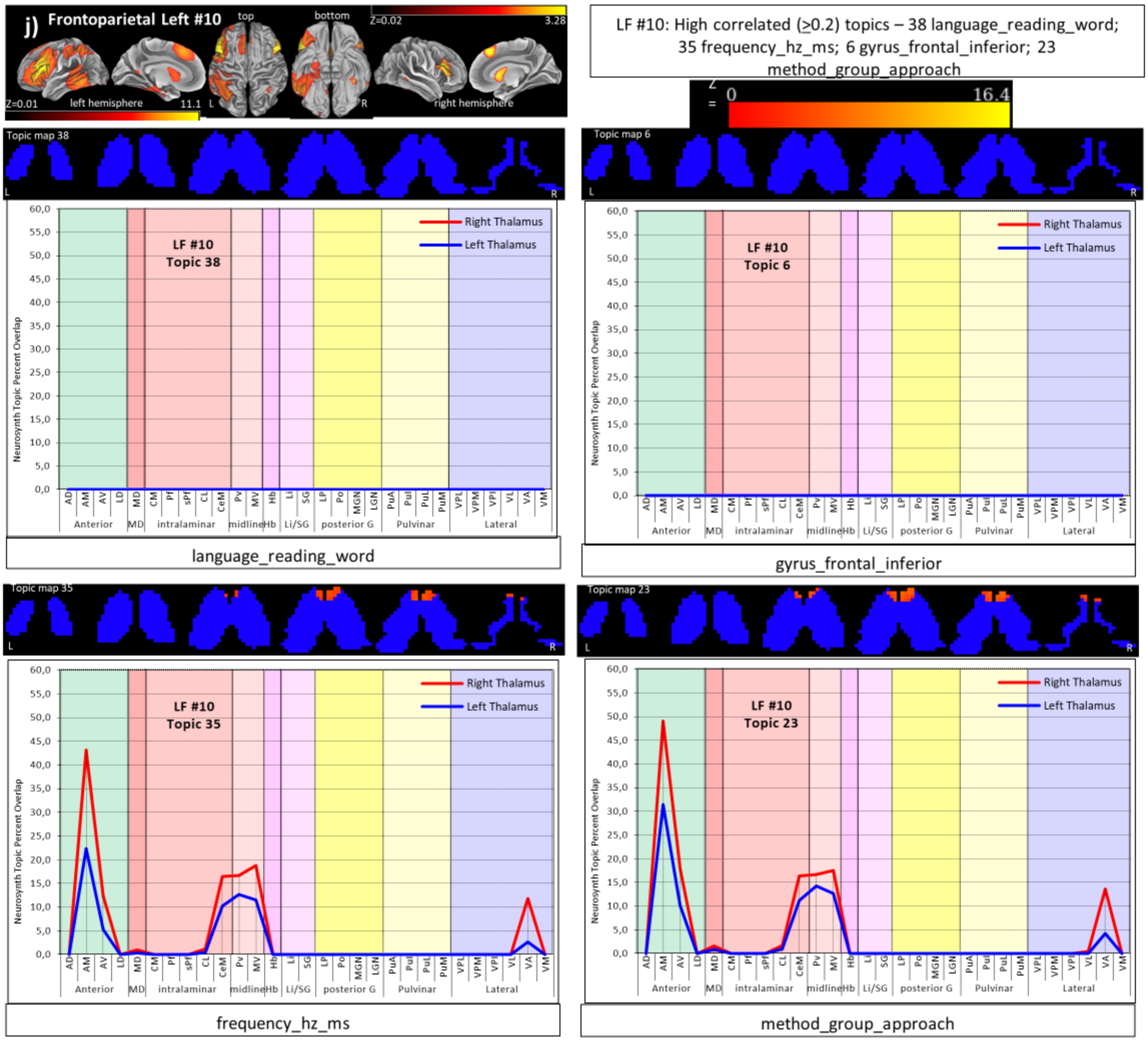

Supplementary Figure 19: LF #10 highly correlated (≥0.2) **neurosynth** topic-maps within Thalamus: each subplot represents a high-correlated topic-map (spatially overlaid on six different axial slices; same as the previous figures 3-5 depicting correlation maps with each network). The topic maps were thresholded at z-value 3.1 (p-value 0.001). The graph within each subplot depicts the percent nuclei overlap of highly correlated topic maps separately (marked in the red circle in Figure 9) with Thalamus. Lfro #10: 38 language_reading_word; 35 frequency_hz_ms; 6 gyrus_frontal_inferior; 23 method_group_approach. Note: no corresponding spatial map within the thalamus was found in topic 38, and 6. Top row shows the corresponding LF network brainmap, used for the neurosynth topic decoding. Color scale: same for all the maps (0 – 16.4) across all the networks. The blue color depicts thalamus mask in the background.

Supplementary Note 1

A) Cytoarchitectonic Characterization of RSN:

Medial, Occipital, and Lateral Visual Network

The medial visual network (MV) includes the primary and secondary visual cortices V1 and V2 as well as bilaterally, the less extended visual areas V3 and V4 . In addition, the areas 7M and 7P in the superior parietal lobe (SPL). The occipital visual Network (OV) is shifted compared to MV to the extrastriate visual cortices V3-V5 but still includes large parts of V1 and V2. An additional involvement of area hlP3 of the intraparietal sulcus (IPS) is now seen, while the SPL areas have almost vanished. The lateral visual network (LV) dominantly includes the visual areas V4-5, while V1-V3 have almost vanished. Within the parietal lobe, areas 5M and 7P of the SPL show right dominance. However, in the adjacent intraparietal lobe (IPL), all seven areas (PGp, Pga, PFt, Pfop, PFm, PFcm, and PF) in both hemispheres are now covered to a varying extent, as well as the right amygdala, BA1-2 of the primary somatosensory cortex (PSMC), and the right Brocca area.

Default, Sensorimotor, and Auditory Network

The default mode network (DMN) encircles the ventral medial prefrontal cortex, the dorsal medial prefrontal cortex, the posterior cingulate cortex, and adjacent precuneus plus the lateral parietal cortex [1–4]. However, in our analysis, the DMN is mainly confined to the SPL, IPL, and IPS of the parietal lobe but does not cover several medio-frontal areas as the JHA is still incomplete [5]; but a comprehensive list according to HOA and BAA is given in the Supplement (Table S2-S3). Nevertheless, our correlation calculation remains unaffected as all corresponding cortical areas were taken into account.

The sensorimotor network (SM) focuses on the pericentral region (BA1-4 and BA6). In the parietal lobe, mainly the postcentral gyrus, major parts of the superior parietal lobe (SPL), the parietal operculum (POP), and hp2 in the intraparietal sulcus (IPS) are bilaterally involved. Other contributions come bilaterally from the supplementary motor cortex (SMA) and the precentral gyrus in the frontal lobe, corresponding to BA1-4 and BA6, with an overall left-sided dominance.

The auditory network (AU) encircles bilaterally all areas of the parietal operculum and all major cortical areas of the primary auditory cortex (PAC) as well as the insula corresponding to BA 13-14, BA 21+22, and BA 41+42. The areas enclose the Heschl gyrus (BA 41), the planum polare, parts of the superior and middle temporal gyrus, and the parietal operculum (POP).

Executive, Right and Left Frontoparietal Network

The major areas in the executive network (EX) are found in three parts of the SPL. Two other contributions arise from the subiculum and dendate gyrus of the right hippocampus, the primary motor cortex (PMC), and the Brocca areas in the frontal lobe.

Corresponding to its name, the right frontoparietal network (RF) mainly comprises the right parietal, frontal, and temporal areas. In the parietal lobe, the right parietal operculum (POP), all areas of the IPL and IPS, including the right supramarginal and the angular gyrus (BA 39+40), are involved. In the temporal lobe, the areas are confined to the right Heschl gyrus and planum temporale. Other relevant contributions come from the right inferior frontal gyrus and central opercular cortex in the frontal lobe.

The Left frontoparietal network (LF) comprises parietal, temporal, and frontal areas, predominantly in the left hemisphere, partly mirroring the RF. The SPL, POP, supramarginal, angular, and postcentral gyrus are involved in the parietal lobe. The areas encircle the left superior, middle, and inferior temporal gyrus in the temporal lobe. Finally, in the frontal lobe, major areas are found in the inferior frontal gyrus and the frontal operculum.

Limitations:

Overlaps between Smith-10:

The spatio-temporal similarities overlap, as there are brain areas shared in more than one large-scale functional network, probably because of the nature of functional architecture and communication demands within the brain.

The Smith-10 maps rely on the ICA approach, an overlap exists in their ICA-based decomposition. If similar ICA employs a higher dimensionality, such overlap breaks down to the least overlapping components. For instance, Stephan Smith and colleagues performed high-dimensional ICA of the human connectome project r-fMRI data up to 300 components (15, 25, 50, 100, 200, 300 dimensions; https://www.humanconnectome.org/storage/app/media/documentation/s900/HCP900_GroupICA+NodeTS+Netmats_Summary_15dec2015.pdf).

In this project, we did not investigate the increasing number of dimensions. However, such investigation will be part of future studies by picking the ICA components representing non-overlapping spatio-temporal similarities with the overlapping spatio-temporal similarities with the increasing number of dimensions.

Supplementary Note 2

Core and Matrix Connectivity:

Results: The percent connectivity values of the core and matrix nuclei are shown (s. Figure S5). The analysis reveals no difference between the both groups i.e., Right cortical functional networks to right thalamus nuclei connectivity (i: two-sample t-test: p-value = 0.418, t=0.81321, ii: Wilcoxon rank-sum test: p-value = 0.5842, and iii: core mean 10.08435, matrix mean 10.03680); and left cortical functional networks to left thalamus nuclei connectivity (i: two-sample t-test: p-value = 0.1438, t=1.4732 ,ii: Wilcoxon rank-sum test: p-value = 0.08354, iii: core mean 10.07339, matrix mean 10.01604).

Discussion: Jones [6] first introduced the core and matrix concept, which offers an interesting functional differentiation between thalamocortical interactions [6–8]. The core nuclei mainly facilitate input to the cortex via layer 4, and matrix nuclei connect with superficial and deeper cortical layers. However, our analysis found no significant difference between the core and matrix groups (Figure 4c). One reason may be that the core and matrix organization is based on cortical layer-specific communication. However, the employed cortical functional network masks cover the whole gray matter ribbon and do not account for layer-specific spatial activation [9]. Secondly, if we overlook the layer-specificity, it may be that core and matrix nuclei's similar connectivity are required during rest, which may suggest that correlative observations in the topic analysis indicate that relay (core) nuclei are involved in different mental functions (Figure 9).

Supplementary Note 3

Correspondence of topic maps with the thalamus (>±0.2) (see Supplementary Figures 10-19):

MV #1: The visual_cortex_sensory and imaginary_mental_events mental functions map to pulvinar and posterior nuclei. In contrast, eye_sleep_gaze mental functions map in a less spatial overlap to different nuclei groups, except for the midline nuclei.

OV #2: The OV also overlaps with visual_cortex_sensory like the MV network. OV shows a specific association with face_faces_facial mental function, covering the posterior, pulvinar, and Li/SG nuclei groups, which may indicate communication to dorsal, ventral, and lateral visual streams to facilitate the functional architecture of face processing.

LV #3: Interestingly, the visual_cortex_sensory appears similar to MV and OV networks. The face_faces_facial mental function also depicts like in the OV network. However, among three visual networks, action_actions_observation is uniquely associated with the LV network. The action_actions_observation function maps to SG, posterior, pulvinar, lateral group, and slightly in the CM intralaminar nuclei.

DMN #4: The memory_retrieval_encoding mental function is associated with Li/SG, posterior, pulvinar, and lateral nuclei groups. Interestingly, the memory_retrieval_encoding function is associated with a default mode network, according to the existing literature.

CB #5: The cerebellar, cereblum_basal, and motor_cortex_hand function show association with all thalamus nuclei.

SM #6: The motor_cortex_hand function show association with almost all thalamus nuclei. In contrast, the stimularion_tms_bpd and learning_traing_practice show minimal overlap with the anterior and pulvinar nuclei.

AU #7: The auditory_speech_temporal mental function maps slightly involve the intralaminar, Li/SG, posterior, and lateral nuclei groups.

EX #8: The decision_making_risk and response_inhibition_control map weekly to similar nuclei groups, i.e., anterior, intralaminar, and lateral groups.

RF #9: The response_inhibition_control slightly overlaps with intralaminar, midline, and lateral nuclei groups. Interestingly, the pain_somatosensory_stimulation mental function is associated with almost all thalamus nuclei except for LGN and PuI.

LF #10: The frequency_hz_ms and method_group_approch resemble each other, showing overlap with the anterior, midline, and lateral nuclei groups.

In summary, examining the diverse mental-function associations in the thalamus (Supplementary Figure 6) reveals that several functions render similar sets of nuclei, indicating the functional multiplicity of thalamic nuclei.

Supplementary References:

Figures:

1. Fox, P. T. et al. BrainMap taxonomy of experimental design: description and evaluation. Hum Brain Mapp 25, 185–198 (2005).
2. Smith, S. M. et al. Correspondence of the brain's functional architecture during activation and rest. PNAS 106, 13040–13045 (2009).
3. Morel, A. Stereotactic Atlas of the Human Thalamus and Basal Ganglia. (CRC Press, 2007).

Supplementary Note:

1. Raichle, M. E. The Brain's Default Mode Network. *Annual Review of Neuroscience* **38**, 433–447 (2015).
2. Shulman, G. L. *et al.* Common Blood Flow Changes across Visual Tasks: II. Decreases in Cerebral Cortex. *Journal of Cognitive Neuroscience* **9**, 648–663 (1997).
3. Buckner, R. L., Andrews-Hanna, J. R. & Schacter, D. L. The brain's default network: anatomy, function, and relevance to disease. *Ann. N. Y. Acad. Sci.* **1124**, 1–38 (2008).
4. Buckner, R. L. & DiNicola, L. M. The brain's default network: updated anatomy, physiology and evolving insights. *Nat Rev Neurosci* **20**, 593–608 (2019).
5. Amunts, K., Mohlberg, H., Bludau, S. & Zilles, K. Julich-Brain: A 3D probabilistic atlas of the human brain's cytoarchitecture. *Science* (2020) doi:10.1126/science.abb4588.
6. Jones, E. G. Viewpoint: the core and matrix of thalamic organization. *Neuroscience* **85**, 331–345 (1998).
7. Müller, E. J. *et al.* Core and matrix thalamic sub-populations relate to spatio-temporal cortical connectivity gradients. *Neuroimage* **222**, 117224 (2020).
8. Piantoni, G., Halgren, E. & Cash, S. S. The Contribution of Thalamocortical Core and Matrix Pathways to Sleep Spindles. *Neural Plasticity* vol. 2016 e3024342 https://www.hindawi.com/journals/np/2016/3024342/ (2016).
9. Sotero, R. C. *et al.* Anatomically-constrained effective connectivity among layers in a cortical column modeled and estimated from local field potentials. *J Integr Neurosci* **9**, 355–379 (2010).
