# Supplementary material for: Relay and higher-order thalamic nuclei show an intertwined functional association with cortical-networks
Source: Commun Biol. 2022 Nov 4;5:1187. doi: 10.1038/s42003-022-04126-w (PMC9636420; doi:10.1038/s42003-022-04126-w)
Supplement: Supplementary file 2 — Description of Additional Supplementary Files [file 42003_2022_4126_MOESM2_ESM.pdf]

Description of Additional Supplementary Files

File name: Supplementary Data 1
Description: The data files contain Large-scale functional networks (MV, OV, LV, DMN, CB, SM, AU, EX, Rfro, Lfro) associated with the decoded Neurosynth topic maps. It represents the smith-10 functional network-specific decoding with the neurosynth LDA-50 topic map. Each network represents decoding in two columns. The first column represents each functional network's feature, and the second correlation (r). The decoding of each functional network relies on the trained correlation decoder, i.e., LDA 50 neurosynth topics. The features and r for each functional network are also graphically illustrated in Figures 8-9.

File name: Supplementary Data 2
Description: The data file contains underlined data for the main and supplementary figures. The excel file contains data for main Figures 1, 3, 4, 7, 8, and 9. The main Figures 2, 5, and 6 don't require underlining quantitative data. Supplementary Figure 3 relies on the data sources from Figures 1, 3, and Supplementary Figure 1. Supplementary Figure 4, visualized maps are available in the figshare.
