# Supplementary material for: Relay and higher-order thalamic nuclei show an intertwined functional association with cortical-networks
Source: Commun Biol. 2022 Nov 4;5:1187. doi: 10.1038/s42003-022-04126-w (PMC9636420; doi:10.1038/s42003-022-04126-w)
Supplement: Supplementary file 5 — Reporting Summary [file 42003_2022_4126_MOESM5_ESM.pdf]

nature research

Corresponding author(s): vinod kumar

Last updated by author(s): Oct 16, 2022

Reporting Summary

Nature Research wishes to improve the reproducibility of the work that we publish. This form provides structure for consistency and transparency in reporting. For further information on Nature Research policies, see our Editorial Policies and the Editorial Policy Checklist.

Statistics

For all statistical analyses, confirm that the following items are present in the figure legend, table legend, main text, or Methods section.

n/a	Confirmed	
☐	☒	The exact sample size (n) for each experimental group/condition, given as a discrete number and unit of measurement
☐	☒	A statement on whether measurements were taken from distinct samples or whether the same sample was measured repeatedly
☒	☐	The statistical test(s) used AND whether they are one- or two-sided<br>*Only common tests should be described solely by name; describe more complex techniques in the Methods section.*
☒	☐	A description of all covariates tested
☒	☐	A description of any assumptions or corrections, such as tests of normality and adjustment for multiple comparisons
☐	☒	A full description of the statistical parameters including central tendency (e.g. means) or other basic estimates (e.g. regression coefficient) AND variation (e.g. standard deviation) or associated estimates of uncertainty (e.g. confidence intervals)
☒	☐	For null hypothesis testing, the test statistic (e.g. F, t, r) with confidence intervals, effect sizes, degrees of freedom and P value noted<br>*Give P values as exact values whenever suitable.*
☒	☐	For Bayesian analysis, information on the choice of priors and Markov chain Monte Carlo settings
☒	☐	For hierarchical and complex designs, identification of the appropriate level for tests and full reporting of outcomes
☒	☐	Estimates of effect sizes (e.g. Cohen's d, Pearson's r), indicating how they were calculated

Our web collection on statistics for biologists contains articles on many of the points above.

Software and code

Policy information about availability of computer code

Data collection	Connectome DB was used to download the Human connectome project datasets: https://db.humanconnectome.org/app/template/Login.vm
Data analysis	All the used tools are freely available.<br>The FSL Software library was used for correlation and statistical analysis.<br>https://fsl.fmrib.ox.ac.uk/fsl/fslwiki (correlation analysis: FSL SBCA)<br>Topic-analysis: https://nimare.readthedocs.io/en/latest/index.html<br>Visulization: https://www.humanconnectome.org/software/connectome-workbench

For manuscripts utilizing custom algorithms or software that are central to the research but not yet described in published literature, software must be made available to editors and reviewers. We strongly encourage code deposition in a community repository (e.g. GitHub). See the Nature Research guidelines for submitting code & software for further information.

Data

Policy information about availability of data

All manuscripts must include a data availability statement. This statement should provide the following information, where applicable:

- Accession codes, unique identifiers, or web links for publicly available datasets
- A list of figures that have associated raw data
- A description of any restrictions on data availability

The datasets analysed during the current study are freely available in the Human connectome project repository (http://www.humanconnectomeproject.org/)

Field-specific reporting

Please select the one below that is the best fit for your research. If you are not sure, read the appropriate sections before making your selection.

☒ Life sciences ☐ Behavioural & social sciences ☐ Ecological, evolutionary & environmental sciences

For a reference copy of the document with all sections, see nature.com/documents/nr-reporting-summary-flat.pdf

Life sciences study design

All studies must disclose on these points even when the disclosure is negative.

Sample size	No sample size calculation, since no effect size to be investigated
Data exclusions	No data excluded.
Replication	No additional replication studies performed in addition to the results presented in the paper.
Randomization	No randomization, since there was no intervention
Blinding	No blinding, since not relevant in the context of this study (correlation between large-scale functional networks and thalamus)

Reporting for specific materials, systems and methods

We require information from authors about some types of materials, experimental systems and methods used in many studies. Here, indicate whether each material, system or method listed is relevant to your study. If you are not sure if a list item applies to your research, read the appropriate section before selecting a response.

Materials & experimental systems

n/a	Involved in the study
☒	☐ Antibodies
☒	☐ Eukaryotic cell lines
☒	☐ Palaeontology and archaeology
☒	☐ Animals and other organisms
☒	☐ Human research participants
☒	☐ Clinical data
☒	☐ Dual use research of concern

Methods

n/a	Involved in the study
☒	☐ ChIP-seq
☒	☐ Flow cytometry
☐	☒ MRI-based neuroimaging

Magnetic resonance imaging

Experimental design

Design type	resting state fMRI
Design specifications	The subjects are asked to lie with eyes open, with “relaxed” fixation on a white cross (on a dark background), think of nothing in particular, and not to fall asleep. The scanner room is darkened.<br>As mentioned in Smith et.al. 2014 (https://www.ncbi.nlm.nih.gov/pmc/articles/PMC3720828/)
Behavioral performance measures	The resting state fMRI measurement in the HCP project, didn't require an extensive measure of behavoural performance.<br>"We chose eyes-open in order to minimise the risk of subjects falling asleep during two successive runs."<br>As mentioned in Smith et.al. 2014 (https://www.ncbi.nlm.nih.gov/pmc/articles/PMC3720828/)

Acquisition

Imaging type(s)	function magnetic resonance imaging (fMRI)
Field strength	Human connectome data was acquired at 3T MRI scanner.
Sequence & imaging parameters	"from: https://www.humanconnectome.org/hcp-protocols-ya-3t-imaging"<br>rfMRI data were acquired in four runs of approximately 15 minutes each, two runs in one session and two in another session, with eyes open with relaxed fixation on a projected bright cross-hair on a dark background (and presented in a darkened room). Within each session, oblique axial acquisitions alternated between phase encoding in a right-to-left (RL) direction in one run and phase encoding in a left-to-right (LR) direction in the other run.

Resting state images were collected with the following parameters:

Sequence: Gradient-echo EPI, TR: 720 ms, TE: 33.1 ms, flip angle: 52 deg, FOV: 208x180 mm (RO x PE), Matrix: 104x90 (RO x PE), Slice thickness: 2.0 mm; 72 slices; 2.0 mm isotropic voxels, Multiband factor: 8, Echo spacing: 0.58 ms, BW: 2290 Hz/Px

Area of acquisition: Whole brain scans

Diffusion MRI: ☐ Used ☒ Not used

Preprocessing

Preprocessing software

We didn't preprocess the data. We downloaded preprocessed, normalized, denoised r-fMRI datasets from the Human connectome project. The analysis pipeline is documented in an amazing paper by Glasser, et. al. 2013. :https://pubmed.ncbi.nlm.nih.gov/23668970/)

Human connectome project used the following pipeline can be found here:
https://github.com/Washington-University/HCPpipelines/releases

Normalization

MNI nonlinear registration. Every frame from the raw timeseries is transformed, according to its unique nonlinear transformation, directly to MNI space with a single spline interpolation, minimizing interpolation-induced blurring.
from: Glasser, et. al 2013. :https://pubmed.ncbi.nlm.nih.gov/23668970/

Normalization template

MNI 2mm

Noise and artifact removal

Human connectome project used FSL-Fix based automatic denoising of the motion and other relevant artefacts. https://fsl.fmrib.ox.ac.uk/fsl/fslwiki/FIX

Volume censoring

It seems the HCP minimal preprocessing didn't included the volume censoring.
from: Glasser, et. al 2013. :https://pubmed.ncbi.nlm.nih.gov/23668970/
"Further preprocessing, such as, significant spatial smoothing, temporal filtering, nuisance regression, or motion censoring (scrubbing), all remove significant amounts of information. Moreover, the preferred strategies for using these techniques remain subjects of debate (Carp, 2011; Fox et al., 2009; Murphy et al., 2009; Power et al., 2012, 2013; Saad et al., 2012; Turner, 2012). Thus, these forms of preprocessing fall outside the scope of “minimal preprocessing,”"

Statistical modeling & inference

Model type and settings

Fixed effect analysis

Effect(s) tested

Fixed-effect of connectivity between large-scale functional networks and thalamus (seed based connectivity)

Specify type of analysis: ☐ Whole brain ☒ ROI-based ☐ Both

Anatomical location(s)

The ROI were provided by Smith et.al. (2009) study. Which are available at : (https://www.fmrib.ox.ac.uk/datasets/brainmap+rsns).

The thalamus ROI was taken from the average mask of thalamus provided within Human Connectome project.

Statistic type for inference
(See Eklund et al. 2016)

The analysis reported in the paper didn't required the Eklund et.al. 2016 purposed method for correction.

Correction

1. Topic analysis: topic maps were thresholded at p 0.001 (FSL function ptoz for that (ptoz 0.001 -2, where 0.001 is the p-value and -2 uses 2-tailed conversion).
2. Comparison of core and matrix nuclei: i) two-sample t-test: t.test(core, matrix), ii) Wilcoxon rank-sum test: Wilcox.test(core, matrix, paired = FALSE, alternative = "two.sided").

Models & analysis

n/a	Involved in the study
☐	☒ Functional and/or effective connectivity
☒	☐ Graph analysis
☒	☐ Multivariate modeling or predictive analysis

Functional and/or effective connectivity

FSL SBCA with 10 networks to thalamus (partial correlation)
